# A Commercial Extract of *Cyanotis arachnoidea* Roots as a Source of Unusual Ecdysteroid Derivatives with Insect Hormone Receptor Binding Activity

**DOI:** 10.1021/acs.jnatprod.0c01274

**Published:** 2021-06-18

**Authors:** Gábor Tóth, Ibolya Herke, Tamás Gáti, Máté Vágvölgyi, Róbert Berkecz, Lyudmila V. Parfenova, Minori Ueno, Taiyo Yokoi, Yoshiaki Nakagawa, Attila Hunyadi

**Affiliations:** † Department of Inorganic and Analytical Chemistry, NMR Group, Budapest University of Technology and Economics, H-1111 Budapest, Hungary; ^‡^Institute of Pharmacognosy, Interdisciplinary Excellence Centre, ^§^Institute of Pharmaceutical Analysis, and ^¶^Interdisciplinary Centre of Natural Products, University of Szeged, H-6720 Szeged, Hungary; ⊥ Servier Research Institute of Medicinal Chemistry (SRIMC), H-1031 Budapest, Hungary; ∥ Institute of Petrochemistry and Catalysis of Russian Academy of Sciences, 450075 Ufa, Russia; □ Graduate School of Agriculture, Kyoto University, Kyoto 606-8502, Japan

## Abstract

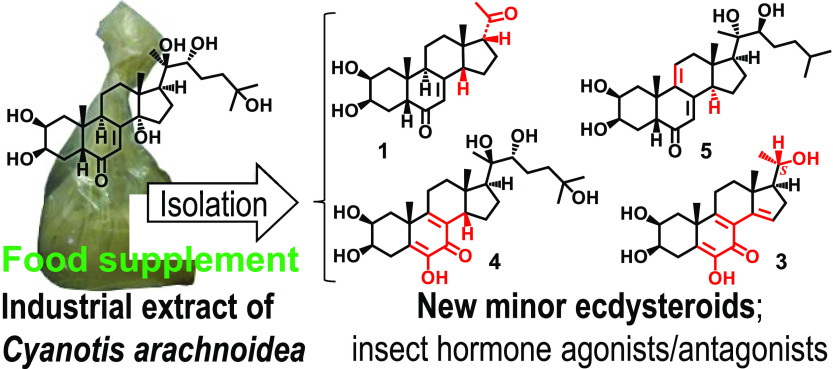

Ecdysteroids act as molting hormones in insects and as nonhormonal anabolic agents and adaptogens in mammals. A wide range of ecdysteroid-containing herbal extracts are available worldwide as food supplements. The aim of this work was to study such an extract as a possible industrial source of new bioactive ecdysteroids. A large-scale chromatographic isolation was performed from an extract of *Cyanotis arachnoidea* roots. Ten ecdysteroids (**1**–**10**) including eight new compounds were isolated and characterized by extensive nuclear magnetic resonance studies. Highly unusual structures were identified, including a H-14β (**1**, **2**, **4**, and **10**) moiety, among which a 14β(H)17β(H) phytosteroid (**1**) is reported for the first time. Compounds with an intact side chain (**4**–**10**) and 11 other natural or semisynthetic ecdysteroids (**11**–**21**) were tested for insect ecdysteroid receptor (EcR) binding activity. Two new compounds, i.e., 14-deoxydacryhainansterone (**5**) and 22-oxodacryhainansterone (**6**), showed strong EcR binding activity (IC_50_ = 41.7 and 380 nM, respectively). Six compounds were identified as EcR agonists and another two as antagonists using a transgenic ecdysteroid reporter gene assay. The present results demonstrate that commercial *C. arachnoidea* extracts are rich in new, unusual bioactive ecdysteroids. Because of the lack of an authentic plant material, the truly biosynthetic or artifactual nature of these compounds cannot be confirmed.

Ecdysteroids represent a particularly versatile group of natural products due to their chemical variability and the broad range of bioactivities they can exert. They are best known as analogues of the insect-molting hormone 20-hydroxyecdysone (20E). Their polar, polyhydroxylated character hinders the absorption of typical phytoecdysteroids through the cuticle of insects; in contrast, they need to be consumed to function as insect hormones, which prevents their use as sprays in pest management. Nevertheless, these compounds serve as models for the rational design of synthetic analogues,^1,2^ rendering the study of their structure–activity relationships important. Ecdysteroids are also bioactive in mammals; some of their representatives, including 20E and its metabolite poststerone,^3,4^ act as nonhormonal, green anabolic agents and adaptogens and offer a wide range of metabolic benefits. As a result, their consumption is typically considered “healthy”. This has led to the production and worldwide marketing of ecdysteroid-containing herbal extracts^5^ for various purposes, particularly as anabolic food supplements for athletes. A simple Internet search revealed that ecdysteroid-containing extracts typically prepared from the roots of *Cyanotis arachnoidea* C.B. Clarke (Commelinaceae) are available for online purchase up to a scale of several metric tons per month at highly competitive prices; depending on the purity, some companies offer extracts at 1 USD/kg. The roots of *C. arachnoidea* are indeed very rich in ecdysteroids, containing as much as 2–3% of these compounds. Several studies reported the isolation of minor phytoecdysteroids from this plant, comprising a total of 22 compounds.^6−10^

Over the past few decades, attempts to translate the chemical complexity of ecdysteroids into possible pharmacological use(s) have been performed in two major directions, namely, (i) the isolation and bioactivity evaluation of new natural compounds and (ii) the extension of the chemical space of these compounds by performing semisynthetic modifications to improve/optimize certain bioactivities and achieve new ones. In the context of this latter strategy, our group has investigated certain structure–activity relationships to explore the effect of these compounds on the drug resistance of cancer cell lines.^11−14^

Unfortunately, the study of the bioactivity of ecdysteroids has been limited by their availability in sufficient amounts. Despite their very high structural diversity, which has led to the discovery of 526 natural ecdysteroids as of November 2020,^15^ the ecdysteroid composition of plants is always dominated by a few major compounds. Among these, 20E is by far the most abundant, and other analogues are present in much lower amounts. Therefore, much research effort has been devoted to the preparation of rare phytoecdysteroids from 20E via semisynthesis. In the present work, our aim was to initiate a large-scale phytochemical investigation into the potential of commercial *Cyanotis* extracts as valuable and plentiful raw materials of ecdysteroids. Although this is a rather unorthodox way to initiate a phytochemical study because the truly biosynthetic or artifactual origin of any compound isolated from such a preprocessed raw material is unknown, these studies are of importance because of the large amounts of *Cyanotis* extracts that are consumed by humans worldwide. Further, the industrial-scale availability of these extracts provides a stable background for a large-scale production and further development of new bioactive compounds for their use as pure substances. As a starting point of our in-depth evaluation of the biological value of compounds present in commercial *Cyanotis* extracts, we first aimed to test newly isolated ecdysteroids for their insect hormone activity, which could pave the way toward the development of new biological and green synthetic plant-protecting agents.

## Results and Discussion

Inspired by an unexpected outcome of a previous study conducted by our group, in which the chromatographic processing of only 5 g of a *C. arachnoidea*-containing food supplement led to the discovery of two new ecdysteroids,^5^ it was decided to initiate an extensive preparative work on a much larger scale (several kilograms) to search for new, minor bioactive ecdysteroid derivatives. It needs to be stressed that in this work the starting material was not a ground plant but an industrial extract purchased online. Nevertheless, the starting material showed a qualitative minor constituent fingerprint using high-performance liquid chromatography (HPLC) photodiode array (PDA), which conformed with that of other *C. arachnoidea* extracts that were independently purchased and used in previous related studies. This supported the manufacturer’s declaration on the botanical identity of the source plant.

Because of the extremely rich ecdysteroid composition of the starting material, an extensive, multistep chromatographic purification was required to obtain the minor compounds. It is worth mentioning that this procedure led to the isolation of many ecdysteroids that are out of the scope of this contribution. Here, we report and discuss 10 compounds (**1**–**10**) that were successfully obtained in this study. For the structural elucidation, we performed a comprehensive one- and two-dimensional (1D and 2D, respectively) NMR analysis,^16,17^ achieving a complete ^1^H and ^13^C NMR signal assignment for all the investigated compounds. Because of the molecular mass of the compounds (∼500 Da), the signal/noise value of the selective rotating-frame Overhauser effect (ROE) experiments strongly exceeded those of the selective nuclear Overhauser effects (NOEs). The ^1^H and ^13^C NMR chemical shifts of compounds **1**–**10** are compiled in Tables 1–3. Characteristic NMR spectra of these compounds, along with their stereostructures, ^1^H and ^13^C assignments, and characteristic HMBC correlations and steric proximities, are presented in Figures S1–S69, Supporting Information.

**Table 1:** ^13^C NMR Chemical Shifts of Compounds **1**–**10**

atom no.	**1**^a^	**2**^a^	**3**^a^	**4**^a^	**5**^b^	**6**^b^	**7**^a^	**8**^a^	**9**^a^	**10**^a^
1	36.5	36.3	41.7	40.3	37.4	37.3	36.6	36.3	42.4	36.3
2	66.8	66.6	68.3	68.0	69.0	68.8	67.0	66.7	68.5	66.5
3	66.7	66.6	72.2	71.7	68.3	68.4	66.3	66.7	70.8	66.6
4	32.0	31.9	27.1	26.8	36.3	35.8	34.8	31.8	24.1	31.8
5	50.1	49.8	133.0	133.2	51.4	51.7	49.7	50.1	53.4	49.7
6	202.1	201.9	142.8	142.6	206.1	206.9	202.2	202.0	198.8	201.9
7	124.0	123.5	179.7	180.2	120.1	119.4	118.7	120.7	122.4	124.1
8	164.2	166.0	123.2	131.5	158.4	156.2	155.1	164.9	163.5	166.3
9	34.2	34.0	164.1	164.7	138.1	136.3	136.1	37.3	50.0	34.6
10	37.6	37.6	41.1	40.5	40.9	40.7	38.6	37.5	37.5	38.1
11	20.6	20.8	23.8	22.4	134.2	133.7	131.6	21.5	21.4	20.4
12	26.9	34.7	34.8	35.0	44.4	39.0	42.4	38.7	38.7	38.3
13	45.2	44.5	44.6	40.4	45.5	48.2	43.6	45.0	44.8	43.7
14	55.9	54.3	141.5	46.4	53.3	84.5	51.5	55.0	54.9	56.8
15	29.9	32.4	126.5	30.2	23.8	31.5	22.2	22.0	22.1	26.6
16	23.9	21.9	35.4	25.2	23.0	22.0	21.5	21.3	21.3	32.4
17	63.1	55.3	57.8	50.8	56.3	51.8	54.4	54.5	54.5	54.6
18	24.2	19.7	16.0	23.8	13.6	18.1	12.9	14.0	14.0	23.9
19	24.2	24.0	27.3	26.5	32.0	31.7	31.2	24.1	15.4	23.7
20	208.8	65.3	67.0	75.6	77.5	81.8	76.2	75.6	75.6	75.8
21	31.6	23.2	24.3	19.9	20.8	25.2	20.6	20.9	20.9	20.0
22				76.4	77.9	217.4	75.4	76.1	76.2	76.4
23				26.0	30.6	35.5	26.1	26.1	26.1	25.9
24				41.3	37.7	33.9	41.4	41.4	41.5	41.3
25				68.7	29.3	28.9	68.8	68.8	68.9	68.7
26				29.0	22.9	22.9	29.1	29.0	29.0	29.0
27				29.9	23.6	22.9	29.8	29.9	30.0	30.0

a Run in DMSO-*d*_6_.

b Run in MeOH-*d*_4_.

**Table 2:** ^1^H NMR Chemical Shifts, Multiplicity, and Coupling Constants *J*_HH_ (in Hz) of Compounds **1**–**5**

no.	**1**^a^	**2**^a^	**3**^a^	**4**^a^	**5**^b^
1β	1.24 t (12.5)	1.24 t (12.5)	2.27 dd (14.1, 3.0)	2.17 dd (14.0, 2.7)	1.71 t (12.5)
1α	1.60	1.60	1.26	1.14	2.06
2α	3.74	3.71	3.83	3.80	3.63
3α	3.74	3.74	3.33	3.31	3.83
4β	1.48	1.48	2.38 t (12.2)	2.35 t (12.2)	1.78
4α	1.44	1.43	2.91 ddd (12.2, 4.6, 1.0)	2.89 dd (12.2, 4.5)	1.42
5β	2.16 dd (12.5, 4.3)	2.16 dd (12.5, 4.3)			2.43 dd (12.5, 4.0)
5α					
7	5.68 d (2.4)	5.60 d (2.3)			5.58
9	2.75 ddd (12.0, 5.0, 2.0)	2.75 ddd (11.0, 5.0, 2.0)			
11β	1.34	1.45	2.52	2.32	6.33
11α	1.70	1.65	2.58	2.22	
12β	1.15	1.25	1.99	1.72	2.67
12α	1.55	1.62	1.34	1.37	2.37
14β	2.47 t (9.5)	2.54 t (9.0)		2.43 t (8.1)	
14α					2.56 ddd (11.0, 7.5, 2.0)
15β	1.69	1.62	6.77 dd (3.2, 2.2)	2.08	1.57
15α	1.84	1.80		0.92	1.93
16β	1.71	1.79	2.35	1.70	2.02
16α	2.10	1.75	2.46	1.50	1.73
17β	2.85 t (9.5)				
17α		1.42	1.58 dt (10.2, 8.2)	1.68	1.89 t (9.5)
18	1.14 s	1.00 s	0.76 s	1.10 s	0.83 s
19	0.80 s	0.80 s	1.40 s	1.36 s	1.11 s
20		3.89 qdd (6.0, 4.6, 2.2)	3.68 dq (14.5, 6.1)		
21	2.11 s	1.00 d (6.0)	1.15 d (6.1)	1.13 s	1.20 s
22				3.20	3.32
23				1.42, 1.13	1.52, 1.24
24				1.64, 1.23	1.48, 1.24
25					1.57
26				1.03 s	0.91 d (6.5)
27				1.05 s	0.93 d (6.5)
HO-2	4.34	4.34	4.64	4.57	
HO-3	4.34	4.34	4.94	4.87	
HO-6			8.10 s	8.02 s	
HO-20		4.22	4.35	3.61 s	
HO-22				4.33	
HO-25				4.07 s	

a Run in DMSO-*d*_6_.

b Run in MeOH-*d*_4_.

**Table 3:** ^1^H NMR Chemical Shifts, Multiplicity, and Coupling Constants *J*_HH_ (in Hz) of Compounds **6**–**10**

no.	**6**^b^	**7**^a^	**8**^a^	**9**^a^	**10**^a^
1β	1.71 t (12.5)	1.53	1.25 t (12.5)	1.89 dd (14.1, 3.2)	1.23 t (12.5)
1α	2.09	1.91	1.58	1.40	1.60
2α	3.73	3.41	3.64	3.75	3.70
3α	3.85	3.64	3.75	3.39	3.74
4β	1.77	1.58	1.48	1.52	1.47
4α	1.59	1.24	1.48	1.68	1.47
5β	2.45 dd (12.5, 4.0)	2.25 dd (12.5, 4.0)	2.18 dd (11.7, 5.7)		2.16 dd (12.2, 4.7)
5α				2.26 dd (12.0, 3.2)	
7	5.74	5.42 s	5.45 t (2.0)	5.52 t (2.2)	5.70 d (2.4)
9			2.59	2.17	2.74
11β	6.30	6.20	1.58	1.56	1.44
11α			1.75	1.74	1.67
12β	2.43	2.56	2.16	2.14	1.44
12α	2.83	2.37	1.50	1.43	1.69
14β					2.30 dd (11.0, 6.7)
14α		2.46 ddd (11.0, 7.5, 2.0)	2.14	2.04 ddd (12.0, 6.5, 1.5)	
15β	1.95	1.42	1.44	1.44	1.63^c^
15α	1.79	1.80	1.54	1.55	1.69^c^
16β	1.79	1.91	1.89	1.88	1.53
16α	1.55	1.59	1.53	1.53	1.70
17β					
17α	2.76 t (9.5)	1.79 t (9.5)	1.67 t (9.5)	1.67 t (9.5)	1.76 t (8.7)
18	0.88 s	0.72 s	0.71 s	0.72 s	1.18 s
19	1.11 s	0.99 s	0.83 s	0.88 s	0.79 s
20					
21	1.40 s	1.09 s	1.09 s	1.09 s	1.09 s
22		3.11	3.10	3.10	3.20
23	2.67	1.49, 1.11	1.46, 1.10	1.47, 1.10	1.44, 1.13
24	1.46	1.65, 1.25	1.64, 1.24	1.64, 1.24	1.65, 1.23
25	1.56				
26	0.92 d (6.5)	1.05 s	1.05 s	1.05 s	1.05 s
27	0.92 d (6.5)	1.07 s	1.07 s	1.07 s	1.07 s
HO-2			4.39	4.51	4.34
HO-3			4.36		4.34
HO-6					
HO-20			3.61	3.60 s	3.61 s
HO-22			4.37	4.36	4.34
HO-25			4.10 s	4.12 s	4.10 s

a Run in DMSO-*d*_6_.

b Run in MeOH-*d*_4_.

c Interchangeable assignments.

The molecular formula of compound **1** was established as C_21_H_30_O_4_ using a high-resolution mass spectroscopy (HRMS) analysis, finding that **1** is a C_21_-ecdysteroid with seven double-bond equivalents. According to the ^1^H and ^13^C NMR data (Figures S1–S11, Supporting Information), this compound contains four rings, a 7-en-6-one (α,β-enone) chromophore group, a 17-acetyl (Me–C=O), and two hydroxy groups attached to the C-2 and C-3 carbon atoms. This suggests that compound **1** could be the 14-deoxy analogue of a series of poststerone derivatives that our group recently reported.^18^ A ^1^H,^1^H–COSY experiment revealed the connectivity of nonoverlapping ^1^H signals within a coupled spin system. To achieve an unambiguous assignment of the overlapping ^1^H signals, a selective 1D TOCSY experiment was performed. Irradiation at H-7 revealed the H-9, H_2_-11, and H_2_-12 signals, and all ^1^H signals in the D ring were detected by irradiating H-17. In the spectra obtained using selective 1D ROE experiments irradiating CH_3_-19, CH_3_-18, and H-14, the hydrogen atoms that were in steric proximity (<5 Å) to those irradiated gave separate signals. A *cis* A/B and *trans* B/C ring junction could be extracted from the observed NOE contact of the H_3_-19 atoms with Hβ-5 and H_3_-18. Simultaneously, the detected steric proximities of H-14/H_3_-18 and H-14/H-17 strongly supported the β orientation of these methine hydrogens and a *cis* C/D ring junction. A selROE experiment irradiating Hα-9 provided further proof for the *cis* A/B, *trans* B/C, and *cis* C/D ring junctions. In addition, the Hβ-17 selROE responses allowed us to clearly distinguish the α and β methylene hydrogens. To achieve a complete ^13^C NMR signal assignment of the DEPTQ spectrum, edited HSQC was used to distinguish the CH_2_ cross-peaks. By introducing the 1D selROE spectrum on H_3_-19 and H_3_-18, respectively, into the HSQC experiment, the ROE signals allow identifying the corresponding C–H cross-peaks. The quaternary carbon signals were identified from the HMBC spectrum, for which the HMBC responses over two and three bonds of H_3_-19, H_3_-18, and H_3_-21 were very effective.

An HRMS measurement allowed establishing the molecular formula C_21_H_30_O_4_ for **2**, which indicates that this compound contains one double bond less than compound **1**. The ^1^H and DEPTQ spectra clearly showed that a HC–OH is present in position 20 instead of a C=O group. The adjacent CH_3_-21 signal appeared as a doublet (*J*_HH_ = 6.3 Hz) at 1.00 ppm, whereas the angular CH_3_-18 signal resonated at 1.00 ppm as a singlet. To unambiguously assign the ^1^H and ^13^C NMR resonances, selTOCSY irradiating H-5, H-7, and H-14 and selROE irradiating H-20, H-14, OH-20, and H-9 and edited HSQC and HMBC (Figures S13–S17, Supporting Information) spectra were recorded. The Hα-9 selROE responses on Hα-2 and Hα-4 proved the existence of a *cis* A/B ring junction, and those on Hα-12 and on Hα-15 revealed *trans* B/C and *cis* C/D ring junctions, respectively. Therefore, this part of the structure is similar to that of compound **1**, and the H-14 hydrogen is in the β orientation. The selROE experiment irradiating the Hβ-14 signal at 2.54 ppm did not show steric proximity with H-17 (1.42 ppm); in contrast, it showed proximity with the OH-20 and H-20 hydrogens. The hydroxy-substituted C-20 atom on the D ring of compound **2** is a stereogenic center. To reveal its absolute configuration, the ^3^*J*(H-17,H-20) coupling constant was determined. When decoupling the CH_3_-21 hydrogens, the complicated H-20 signal at δ 3.89 ppm was simplified into a doublet of doublets with couplings of 4.6 and 2.2 Hz. According to the splitting of 4.6 Hz of the OH-20 signal at δ 4.22 ppm, ^3^*J*(H-17,H-20) was determined to be 2.2 Hz, suggesting their gauche arrangement. Moreover, the existence of a strong ROE contact of H-20 only with CH_3_-18, CH_3_-21, HO-20, and Hα-17 reveals the *S* configuration of the stereocenter at C-20.

The molecular formula of **3** was established as C_21_H_28_O_5_ by means of HRMS. The number of double-bond equivalents increased to eight, indicating that this compound contains four rings and four double bonds. Compound **3** exhibited a characteristic UV spectrum like that of calonysterone. The DEPTQ spectrum of **3** showed 21 ^13^C NMR signals, indicating the presence of three methyl, five methylene, four sp^3^ CH methine, and one sp^2^ =CH group and two quaternary sp^3^ and six quaternary sp^2^ carbon atoms, one of which is a cross-conjugated C=O (δ 179.7 ppm). The HMBC correlations of H_3_-19 allowed assigning the quaternary C-10 and H_2_C-1 methylene moieties (1.40/41.1 and 1.40/41.7, respectively), and the 1.40/133.0 and 1.40/164.1 correlations proved the presence of quaternary sp^2^ =C atoms at C-5 and C-9. Thus, the B ring was assigned as a Δ^5,6^-7-one-Δ^8,9^ chromophore. The HMBC cross-peaks H_3_-18/C-14 (0.76/141.5) and H-15/C-13 (6.77/44.6) revealed the presence of a Δ^14,15^ C=CH ethylene moiety. Because the methyl signal at 1.15 ppm in the ^1^H spectrum appeared as a doublet (*J*_HH_ = 6.5 Hz), the presence of a CH_3_–CH–OH substituent in the C-17 position could be concluded. SelTOCSY experiments irradiating the CH_3_-21 signal allowed identifying the spin system H-20, H-17, H_2_-16, H-15, and OH-20, and irradiation at Hα-4 revealed the ^1^H signals around the A ring. Further, irradiation at Hβ-12 allowed differentiating the hydrogens of the C ring. Selective 1D ROE experiments on CH_3_-18, CH_3_-19, and CH_3_-21 differentiated between the α and β orientation of each hydrogen atom. CH_3_-18 presented strong ROE contacts with the H-20 and CH_3_-21 hydrogens, and CH_3_-21 exhibited a strong response on Hβ-12. Taken together, these results provide strong evidence for the *S* configuration of the stereocenter at C-20. The quartet multiplicity of the Hα-17 signal at 1.58 ppm, which exhibited a ^3^*J*(H-17,H-20) value of approximately 10 Hz, indicates a nearly antiperiplanar arrangement of these hydrogens, in agreement with the *S* configuration at C-20. The edited HSQC and HMBC spectra also supported the complete and unambiguous ^13^C signal NMR assignment. Compound **3** was given the trivial name bathoristerone to honor Prof. Mária Báthori on her 80th birthday; she made an extraordinary contribution to the ecdysteroid field with the discovery of approximately a quarter of the currently known phytoecdysteroids.

The HRMS measurement of **4** allowed establishing the molecular formula C_27_H_42_O_7_ as a C_27_-ecdysteroid-containing four rings and three double bonds. For the structure elucidation and NMR signal assignments, the same type of NMR spectra (^1^H, selTOCSY irradiating Hβ-14 + selROE irradiating Hβ-14 and CH_3_-18, DEPTQ, edHSQC, and HMBC; Figures S24–S28, Supporting Information) were recorded as above. The detected ^1^H and ^13^C NMR chemical shifts (see Tables 1 and 2) of the steroid core were similar to those of compound **3**, except for the Δ^14,15^ C=CH signals of the latter, which were replaced with those of a CH–CH_2_ moiety (2.43/46.4 and 2.08 ppm; 0.92/30.2 ppm). The ^13^C NMR chemical shifts of the B ring showed the presence of a Δ^5,6^-7-one-Δ^8,9^ chromophore. To unequivocally assign the very closely located C-1, C-10, and C-13 signals around 40 ppm, band-selective HMBC measurement was performed with a digital resolution of 8 Hz per point in the F_1_ dimension. The C-20–C-27 substituent attached to C-17 was as expected for C_27_-ecdysteroids such as calonysterone and 20E. Therefore, compound **4** was suspected to be a 14,15-dihydro-calonysterone. A selTOCSY experiment irradiating the H-14 signal allowed assigning the H_2_-15, H_2_-16, and H-17 spin system, and a selROE experiment irradiating CH_3_-18 showed a strong ROE contact with H-14, whose β-configuration was thereby confirmed. Meanwhile, the steric proximity between the CH_3_-18 and HO-20 hydrogen atoms and the absence of any correlation with H-17 revealed the α-configuration of H-17. A selROE experiment irradiating the Hβ-14 resonance demonstrated steric proximity with the HO-20, Hβ-15, Hβ-16, and CH_3_-18 hydrogens, supporting these assignments. Compound **4** can be therefore assigned as 14β,15-dihydrocalonysterone.

Compound **5** is also a C_27_-ecdysteroid of molecular formula C_27_H_42_O_5_, containing four rings and three double bonds, according to the corresponding HRMS measurement. For its structure elucidation and NMR signal assignments, ^1^H NMR; selTOCSY irradiating H-11, H-2, Hα-14, and H-22; selROE irradiating CH_3_-21, CH_3_-19, and CH_3_-18; DEPTQ, HSQC, edHSQC, and HMBC spectra were measured (Figures S29–S35, Supporting Information). The DEPTQ spectrum of **5** exhibited 27 ^13^C NMR signals, indicating the presence of five methyl, seven methylene, three sp^3^ HC–O, four CH methine, and two sp^2^ =CH groups and three quaternary sp^3^ and three quaternary sp^2^ carbon atoms, one of which is a conjugated C=O (δ 206.1 ppm). In the ^1^H NMR spectrum, two =CH, three sp^3^ HC–O, three singlet CH_3_, and two doublet CH_3_ signals appeared separately. The doublet multiplicity of the latter signals indicated the presence of one CH unit in position 25. The HMBC correlations of H_3_-19 (1.11/37.4, 1.11/40.9, and 1.11/51.4) allowed assigning the quaternary C-10, H_2_C-1 methylene, and HC-5 methine moieties, respectively, and the 1.11/138.1 cross-peak revealed the presence of a quaternary sp^2^ C atom in the Δ^9,11^ double bond. The olefinic H-7 singlet (5.58 ppm) and H-11 doublet (6.33 ppm, *J*_HH_ = 6.7 and 2.0 Hz) and their HMBC couplings with C-5, C-14, and C-9, and C-10, C-13, and C-8, respectively, provided strong evidence for a 7,9(11)-dien-6-one chromophore. The HMBC correlations of H_3_-18 allowed identifying the H_2_C-12 methylene, the quaternary C-13, and the HC-14 and HC-17 methine carbon atoms. The strong H_3_-19/Hβ-5 ROE response revealed a *cis* A/B ring junction, and the absence of strong H_3_-18/H-14 contact indicated a *trans* C/D ring connection. All these results confirm the 14-deoxy-14α-dacryhainansterone structure proposed for compound **5**. These data were in agreement with the ^13^C NMR spectrum of dacryhainansterone containing a 14α-OH substituent reported by Bourne et al.^19^ According to the known significant paramagnetic effects induced by an −OH group in α (δC-14 from 53.5 to 83.1 ppm) and β positions (δC-13 from 45.4 to 48.0 ppm and δC-15 from 23.8 to 29.1 ppm), the observed chemical shift differences further support the suggested structure.

On the basis of an HRMS measurement, the molecular formula C_27_H_40_O_6_ was established for **6**, which is a C_27_-ecdysteroid containing four rings and four double bonds and one oxygen atom more and two hydrogen atoms less than compound **5**. ^1^H NMR, ^13^C + DEPT-135, edHSQC, HMBC, and 2D-NOESY spectroscopy measurements were conducted for the structure elucidation and NMR signal assignments of **6** (Figures S36–S40, Supporting Information). The ^1^H and ^13^C NMR chemical shifts (Tables 1 and 2) of **6** were rather similar to those of compound **5**, confirming the presence of a 7,9(11)-dien-6-one chromophore. However, the ^13^C NMR spectrum of **6** showed several differences with respect to that of **5**; that is, the δC-14 signal shifted from 53.5 to 84.5 ppm, proving an OH substitution, and the δC-22 resonance shifted from 77.9 to 217.4 ppm, indicating the presence of a C=O instead of an HC–OH moiety. The H_3_-18/C-14 (0.88/84.5 ppm) and H_3_-21/C-22 (1.40/217.4 ppm) signals could be assigned on the basis of their HMBC cross-peaks. All other elements of the molecule were identical to those of compound **5**. The H_3_-19/H-5 (1.11/2.45 ppm) NOE correlation supported a *cis* A/B junction, and the NOE correlations of H_3_-18 (0.88 ppm) with δHβ-15 (1.95 ppm) and δHβ-16 (1.79 ppm) supported a *trans* C/D ring junction.

Next, the molecular formula of compound **7** was established as C_27_H_42_O_6_ by means of HRMS. This compound contains four rings and three double bonds and one oxygen atom more than **5**, suggesting that one hydrogen atom of the latter is replaced with a hydroxy group. The structure of **7** was elucidated on the basis of ^1^H NMR, ^1^H,^1^H-COSY, selROE irradiating CH_3_-19 and CH_3_-18, ^13^C, edHSQC, and HMBC spectra (Figures S41–S47, Supporting Information). The appearance of 27 signals in the ^13^C NMR spectrum of **7** indicated the presence of five methyl, seven methylene, three sp^3^ HC–O, three CH methine, and two sp^2^ =CH groups and four quaternary sp^3^ and three quaternary sp^2^ carbon atoms, one of which is a conjugated C=O (δ 202.2 ppm). The δC-4 signal at 34.8 ppm was rather broad probably due to hindered conformational motion; however, the H_2_C-4 signals were detectable in the edHSQC spectrum. In the ^1^H spectrum, two =CH (11 and 7 ppm) and five CH_3_ (21, 27, 26, 19, and 18 ppm) singlets appeared separately. The fact that the CH_3_-27 and CH_3_-26 signals appeared as singlets revealed the presence of an −OH group in position 25. The HMBC correlations of H_3_-19 with the quaternary C-10, the H_2_C-1 methylene, and the HC-5 methine moieties (0.99/38.6, 0.99/36.6, and 0.99/49.7, respectively), the cross-peak at 0.99/136.1 with the quaternary C-10, the doublet of doublets at 6.20 ppm (*J*_HH_ = 6.5 and 2.0 Hz) attributable to H-11 and its HMBC coupling with C-10, C-13, and C-8, and the singlet at 5.42 ppm and its HMBC coupling with C-5, C-14, and C-9 provided strong evidence for a 7,9(11)-dien-6-one chromophore. The HMBC correlations of H_3_-18 allowed identifying the H_2_C-12 methylene, the quaternary C-13, and the HC-14 and HC-17 methine carbon atoms. The strong H_3_-19/Hβ-5 ROE response revealed a *cis* A/B ring junction, whereas the ROE correlation of H_3_-18 with Hβ-15 (1.42 ppm) and Hβ-16 (1.91 ppm) and the absence of any H_3_-18/H-14 cross-peak supported a *trans* C/D ring connection. According to these results, compound **7** was identified as a 25-hydroxy derivative of compound **5**. Although the two samples were measured in different solvents (**5** in methanol (MeOH)-*d*_4_ and **7** in dimethyl sulfoxide (DMSO)-*d*_6_), their ^13^C NMR chemical shifts were in agreement, and the significant paramagnetic shift of δC-25 (from 29.3 to 68.8 ppm) supported the presence of an −OH substituent at position 25. Compound **7** was previously identified as a semisynthetic byproduct from the acidic hydrolysis of 20E, and it was characterized in D_2_O;^19^ here, we report it as a possible natural product and provide its complete NMR characterization in MeOH-*d*_4_.

Meanwhile, an HRMS analysis of compound **8** revealed its molecular formula as C_27_H_44_O_6_. Compound **8** contains four rings and two double bonds and has two hydrogens more than compound **7**. Structure elucidation and NMR signal assignments of **8** were performed by means of the ^1^H NMR, selTOCSY irradiating Hα-11 and Hβ-16, selROE irradiating H_3_-18 and H_3_-19, DEPTQ, HSQC, edHSQC, and band-selective HSQC and HMBC spectra (Figures S48–S53, Supporting Information). Compound **8** is structurally similar to **7**, except for the Δ^9,11^ C=CH moiety, which is replaced with a CH–CH_2_ group. In the ^1^H NMR spectrum, there is only one =CH signal for H-7 at 5.52 ppm that appears as a triplet (*J*_HH_ ≈ 2 Hz). The exact chemical shifts of the C ring hydrogens were identified on the basis of the selTOCSY irradiating Hα-11, and that irradiating Hβ-16 allowed assigning the hydrogens of the D ring. The selROE experiments irradiating H_3_-18 and H_3_-19 not only revealed the α/β position of the hydrogens but also proved the existence of *cis* A/B (H_3_-19/Hβ-5 contact) and *trans* B/C (H_3_-18/H_3_-19 contact) ring junctions. The H_3_-19 hydrogens correlated with the Hβ-11, Hβ-15, and Hβ-16 signals but not with H-14, which supported the *trans* C/D ring junction and the Hα-14 configuration. The appearance of the δC-9 methine (37.3 ppm) and δC-11 methylene (21.5 ppm) signals in the DEPTQ spectrum, together with the HSQC and HMBC results, unequivocally proved the 14α-deoxy-20-hydroxyecdysone structure. This compound was first reported by Harmatha et al., who obtained its NMR data in MeOH-*d*_4_.^20^ The quaternary carbon signals were identified from the HMBC spectrum, for which the HMBC responses over two and three bonds of the H_3_-19, H_3_-18, H_3_-21, H_3_-26, and H_3_-27 hydrogens were very effective. The HMBC correlations of the −OH hydrogens allowed their identification. For the unambiguous assignment of the three very close C-16, C-11, and C-15 signals (21.3, 21.5, and 22.0 ppm, respectively), band-selective HSQC was the method of choice.

The HRMS measurement of **9** afforded the same molecular formula as for compound **8** (C_27_H_44_O_6_). Their ^1^H and ^13^C spectra were also similar and showed the same structural elements, indicating that compounds **9** and **8** are structural isomers. When comparing the ^13^C NMR chemicals shifts of compounds **8** and **9**, significant changes were observed only for the C-1 (from 36.3 to 42.4 ppm), C-4 (from 31.8 to 24.1 ppm), C-5 (from 50.1 to 53.4 ppm), C-9 (from 37.3 to 50.0 ppm), and C-19 (from 24.1 to 15.4 ppm) signals; the rest of the resonances were essentially the same. The ^1^H and ^13^C NMR chemical shifts of the CH_*n*_ units were detectable from the HSQC and edited HSQC spectra; however, the clear identification of C-11, C-15, and C-16 around 20–21 ppm failed because of insufficient resolution. This problem was circumvented by performing band-selective HSQC. A selTOCSY correlation with 10 ms of mixing time starting from Hα-14 (2.04 ppm) unveiled the H_2_-15 signals, and the H_2_-16 signals also became visible with a mixing time of 40 ms. Furthermore, the partially overlapping H-15/H-16 multiplets were resolved by introducing the selTOCSY spectra into the 2D band-selective HSQC spectrum. A selROE experiment irradiating Hα-14 afforded correlations with Hα-9, Hα-11, and Hα-17, which proved a *trans* C/D ring junction and the α-configuration of H-14. CH_3_-18 presented strong ROE contacts with the Hβ-11, CH_3_-19, and Hβ-12 hydrogens, providing strong evidence for a *trans* B/C ring junction. In contrast with compound **8**, no CH_3_-19/H-5 interaction was observed for compound **9**; instead, a selROE experiment irradiating the H_3_-19 resonance revealed steric proximity with the Hβ-4 hydrogen, which demonstrates the *trans* A/B ring junction and the α-configuration of the H-5 atom. Accordingly, it can be concluded that compound **9** is a 5α-epimer of **8**. To achieve a complete and reliable ^1^H and ^13^C NMR signal assignment, 1D selROE and selTOCSY experiments with high digital resolution were combined with 2D HSQC. This method is highly efficient, as shown by the two examples provided in the Supporting Information, i.e., the introduction of selROE and selTOCSY spectra obtained by irradiating Hα-14 into the edHSQC spectrum and subsequent incorporation of two selROE spectra (Figures S60 and S61, Supporting Information). The quaternary carbon signals were identified by the HMBC responses of the five methyl groups over two and three bonds.

According to an HRMS measurement, the molecular formula of compound **10** is identical to that of compound **8** (C_27_H_44_O_6_). The similarities between the ^1^H and DEPTQ spectra of **8** and **10** indicate that both compounds are structural isomers. When comparing the ^13^C NMR chemical shifts of compounds **8** and **10**, noticeable changes were observed only at the C-15 (from 22.0 to 26.6 ppm), C-16 (from 21.3 to 32.4 ppm), and C-18 (from 14.0 to 23.9 ppm) signals. A selTOCSY experiment irradiating Hβ-14 allowed assigning the chemical shifts of the hydrogens located in the D ring as one spin system, and a selROE experiment irradiating Hβ-14 revealed that H_3_-18, OH-20, Hβ-16, and H-7 were located in steric proximity to H-14. The *cis* C/D ring connection and the β-configuration of H-14 were thereby demonstrated. A TOCSY correlation of Hα-9 with the H_2_-11, H_2_-12, and H-7 signals enabled their assignment. A selROE experiment irradiating H_3_-18 showed a correlation with Hβ-14, which further supported the *cis* C/D ring connection, and ROE responses were detected on Hβ-11 and H_3_-19, which confirmed the *trans* B/C ring annulation. A selROE experiment irradiating H_3_-19 resulted in a strong contact with Hβ-5, further confirming the *cis* A/B ring junction. Therefore, compound **10** is a 14β-epimer of **8**. Unambiguous ^1^H and ^13^C NMR assignment of the CH_*n*_ units was achieved using the HSQC and edited HSQC spectra, and the HMBC spectrum enabled the identification of the quaternary carbon signals.

**Figure 1:**
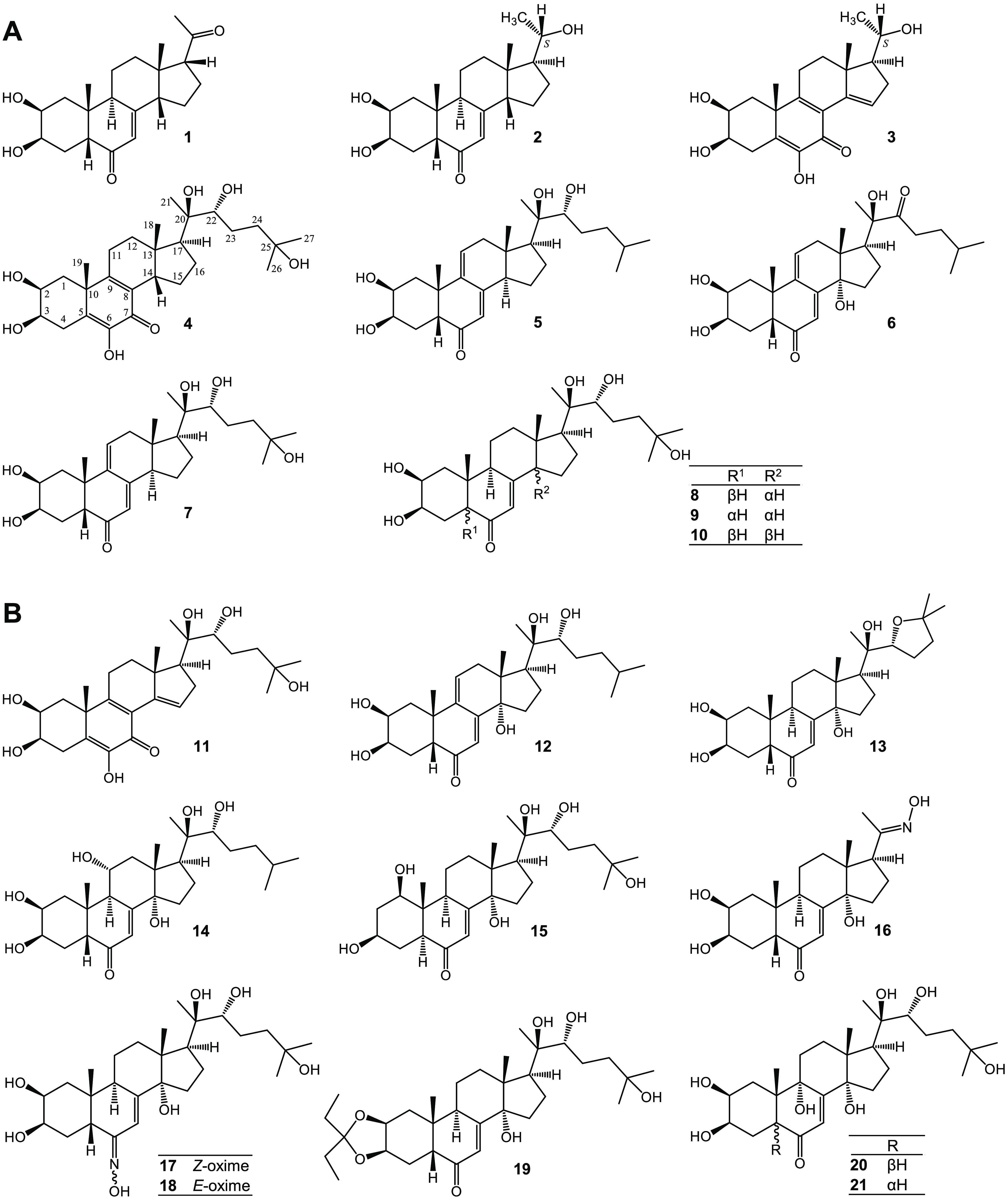
Structures of compounds **1**–**21**. (A) Compounds isolated from a commercial extract of *Cyanotis arachnoidea* (**1**–**10**) and (B) natural (**11**–**15**) and semisynthetic (**16**–**21**) ecdysteroids prepared during previous studies.

Figure 1 displays the structures of compounds **1**–**10** prepared in this study and compounds **11**–**21** used for evaluating the bioactivity.

Several new ecdysteroids (**1**–**10**) with highly unusual structures were obtained during this study. First, compound **1** possesses the 14β(H)17β(H) configuration expected for a synthetic compound; to the best of our knowledge, this is the first time such a steroid was isolated from a plant source. Natural sterols, the precursors in the biosynthesis of ecdysteroids in plants, typically have a 17β-oriented side chain,^21^ as do all phytoecdysteroids isolated to date. It must be stressed that the total synthesis of ecdysteroids requires many stereoselective steps and is much less economical than their isolation from plants;^22^ therefore, adulteration of the extract with synthetic analogues would be expensive and pointless. It also seems highly improbable that any processing aiming to enrich the 20E content during an extract preparation could result in the 17-epimerization of an ecdysteroid. Meanwhile, 14β(H)17β(H) steranes are major marker compounds of petroleum^23,24^ and typically formed during diagenesis under hypersaline conditions.^25^ Taking this and the industrial origin of the extract into account, it could be hypothesized that the plant roots could absorb a petroleum-originated sterane from polluted ground and utilize it for ecdysteroid biosynthesis. Polycyclic terpanes and steranes are involved in bioremediation,^26^ i.e., an ecofriendly approach for transforming environmental pollutants to nontoxic substances by microorganisms and plants.^27^ Finding evidence of the effective utilization of such pollutants by plants for the biosynthesis of unusual secondary metabolites such as compound **1** would be of extraordinary interest and would have many pharmacological and toxicological implications. Although the present results seem to represent an indirect evidence for this, it should be confirmed by additional experiments.

The 14-deoxyecdysteroid compounds (**2**, **4**, **5**, and **7**–**10**) also exhibit unusual structures, especially those with an H-14β substituent (**1**, **2**, **4**, and **10**) as natural products. Ecdysteroids with a *cis* C/D ring junction containing an OH-14β group are very rare, and only two examples had been reported from plant sources up to now, i.e., 14-*epi*-20-hydroxyecdysone from *Serratula wolffii* Andrae^28^ and 14-*epi*-ponasterone A 22-glucoside from *Leuzea carthamoides* Willd.^29^ Similarly, only two naturally occurring 14-deoxyecdysteroids have been reported before, both containing the common *trans* C/D ring junction and an H-14α substituent: 14-deoxyecdysone and 14-deoxy-20-hydroxyecdysone (i.e., compound **5** in this study), which were identified as *in vivo* metabolites of exogenous ecdysone and 20E, respectively, in animal species such as the cricket (*Gryllus bimaculatus*)^30^ or mice.^31,32^ This is the first report on 14-deoxyecdysteroids with a *cis* C/D ring junction isolated from a plant extract. Meanwhile, compound **7** and its 14β-epimer were previously reported as minor products of an intensive overnight acidic treatment of 20E (100 μL of 10 N HCl in 2.0 mL of EtOH) to synthesize stachysterone B.^19^ Such harsh conditions are not suitable for an optimized industrial extraction procedure aiming to maximize the yield of 20E. Further, as it was reported in our previous work, the extract is particularly rich in 2- and 3-acetates of 20E,^5^ whose acid sensitivity suggests that the extract could not have undergone any strong acidic treatment, indicating that compound **7** is most likely of biosynthetic origin. Nevertheless, its acid-catalyzed formation as an artifact of 20E cannot be ruled out.

On the basis of known structure–activity relationships,^33^ the shortened side chain of compounds **1**–**3** was expected to render them inactive as insect hormones. Therefore, of the 10 compounds isolated in this work, only **4**–**10** were examined for their ecdysteroid receptor (EcR) binding affinity, along with previously prepared natural or semisynthetic ecdysteroids **11**–**21** to gain more insight into the structure–activity relationships. The results of the bioactivity evaluation are summarized in Table 4.

**Table 4:** Ecdysteroid Receptor Binding Affinity of Compounds **4**–**21**

compound	trivial name	pIC_50_ (M)^a^ (inh% at 25 μM)^b^
**4**	14β-14,15-dihydrocalonysterone	≈4.60 (49.8%)
**5**	14-deoxydacryhainansterone	7.38
**6**	20-oxodacryhainansterone	6.42 ± 0.04
**7**	14-deoxy-25-hydroxydacryhainansterone	6.32 ± 0.03
**8**	14-deoxy-20-hydroxyecdysone	7.16
**9**	5α-14-deoxy-20-hydroxyecdysone	<4.60 (0.93%)
**10**	5α,14β-14-deoxy-20-hydroxyecdysone	<4.60 (18.2%)
**11**	calonysterone	<4.60 (0%)
**12**	dacryhainansterone	7.64
**13**	shidasterone	5.60
**14**	ajugasterone C	6.19
**15**	5α-2-deoxyintegristerone A	<4.60 (2.42%)
**16**	poststerone 20(*E*)-oxime	<4.60 (4.3%)
**17**	20-hydroxyecdysone 6(*Z*)-oxime	<4.60 (4.53%)
**18**	20-hydroxyecdysone 6(*E*)-oxime	≈4.60 (60.1%)
**19**	28,28-diethyl-2,3-methylidene-20-hydroxyecdysone	5.52
**20**	9α,20-dihydroxyecdysone	<4.60 (45.7%)
**21**	5α-9α,20-dihydroxyecdysone	<4.60 (27.1%)
20E^c^	20-hydroxyecdysone	6.78
PonA^c^	ponasterone A	8.05

a pIC_50_ values are given either as mean ± standard deviation of two parallel measurements (**6** and **7**) or as the result of a single experiment (**5**, **8**, **12**–**14**, and **19**).

b IC_50_ was not determined for compounds showing weak binding at 25 μM; for these, the inhibition (inh%) is given at this concentration.

c Used as positive controls.

Some interesting new structure–activity relationships (SARs) were found in this study. Two compounds, 14-deoxydacryhainansterone (**5**) and dacryhainansterone (**12**), showed strong receptor binding affinities with low IC_50_ values (42 and 23 nM, respectively). The new compound **5** was approximately 4 times more potent than the natural molting hormone 20E (pIC_50_ = 6.78), although still less active than PonA (pIC_50_ = 8.05). The chemical structure of compound **12** is closely related to that of PonA, differing only in the 9,11-bond. The EcR binding results of these two compounds suggest that a 9,11-saturated bond (as in PonA) is slightly more beneficial than an olefin (as in **12**) for this activity, leading to approximately 4.7 times higher binding affinity for PonA than for compound **12**. According to Nakanishi, the activity of muristerone A against Kc cells was enhanced by removing the OH-14 group,^34^ which is the general trend for other ecdysteroids. This is consistent with the present results showing that compound **8** exhibits 2.4 times higher EcR binding affinity than 20E. Interestingly, this was not the case for the 9,11-unsaturated compounds, in which the retained OH-14α group led to higher EcR binding affinity (compound **5** vs **12**). Consistent SAR was found for the OH-25 group, i.e., an increased binding affinity in its absence (20E vs PonA and compound **7** vs **5**). The presence of a C-22 keto group, however, markedly decreased the EcR binding affinity (compound **6** vs **12**).

Compounds **9**, **15**, and **21** having a stereostructure different from that of the natural insect hormone 20E, that is, a *trans* A/B ring junction, showed very weak activity. This is coherent with a previous structure–activity relationship study of PonA analogues, according to which the conversion of the A/B ring junction from *trans* to *cis* enhanced the binding affinity by approximately 100 times.^35^

**Figure 2:**
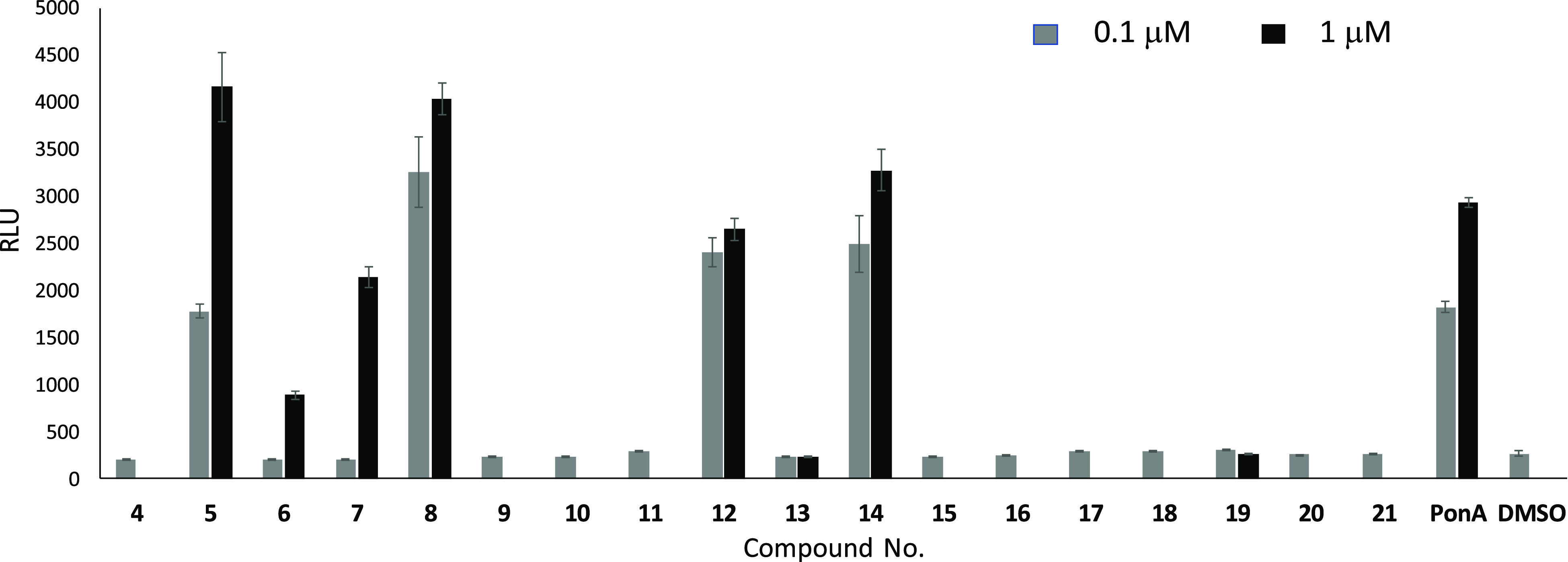
Insect-molting hormone activity of compounds **4**–**21** by a reporter gene assay. Compounds were tested at a concentration of 0.1 or 1 μM. Ponasterone A (PonA) was used as a positive control, and dimethyl sulfoxide (DMSO) as a negative control. RLU: reporter luminescence. Error bars represent the standard deviation, *n* = 4 (**4**, **9**–**11**, **15**–**18**, **20**, **21**, and PonA), *n* = 8 (DMSO), or *n* = 12 (**5**–**8**, **12**–**14**, and **19**).

To distinguish more clearly between agonistic and antagonistic activity, a reporter gene assay was also performed, and the results are shown in Figure 2.

According to the present results, compounds **5**, **8**, **12**, and **14** acted as agonists at a concentration of 0.1 μM. Even though compounds **6** and **7**, for which the binding activity was moderate, did not show agonistic activity at 0.1 μM, they were agonists at a higher concentration (1 μM). In contrast, compounds **13** and **19**, with pIC_50_ values of 5.60 and 5.52 in the binding assay, respectively, did not activate the reporter gene even at 1 μM. This suggests strongly that these compounds were ecdysteroid antagonists. The sterol-type hydroxyalkyl side chain of compounds **5**, **7**, **8**, **12**, and **14** is the same as that of 20E or PonA. In the case of compound **6**, the only difference with the side chain of PonA is the oxo group at C-22 instead of an OH-22 group. Meanwhile, the structures of **13** and **19** showed substantial differences as compared with those of the agonists (**5**–**8**, **12**, and **14**). The short and bulky side chain of **13** resembles the structure of the potent ecdysteroid antagonist ajugalactone,^36^ which can explain its activity. To the best of our knowledge, this is the first report describing a semisynthetic modification on the A ring of an ecdysteroid turning a potent EcR agonist (20E) into an antagonist (**19**).

## Experimental Section

### General Experimental Procedures

Optical rotations were measured with an InsMark IP-Digi1 polarimeter (Shanghai InsMark Instrument Technology Co., Ltd., Shanghai, People’s Republic of China) in MeOH. ^1^H (600 and 500 MHz) and ^13^C (150 and 125 MHz) NMR spectra were recorded at room temperature on Bruker Avance III NMR spectrometers equipped with Prodigy and cryo probeheads, using MeOH-*d*_4_ or DMSO-*d*_6_ as solvents. Chemical shifts (δ) are given on a δ scale and referenced to the solvents (CH_3_OH-*d*_4_: δ_H_ = 3.31 and δ_C_ = 49.1 ppm, and DMSO-*d*_6_: δ_H_ = 2.50 and δ_C_ = 39.5 ppm), and coupling constant (*J*) values are expressed in Hz. Approximately 1–5 mg of compounds were measured in 2.5 mm Bruker MATCH NMR sample tubes or in 5 mm NMR sample tubes. Pulse programs for all the experiments, i.e., ^1^H and ^13^C NMR, DEPTQ, DEPT-135, 1D sel-TOCSY, 1D sel-ROE (τ_mix_: 300 ms), 2D ^1^H,^1^H–COSY, ^1^H,^1^H-NOESY, HSQC, edHSQC, and HMBC (optimized for 8 Hz), were taken from the Bruker software library (TopSpin 3.5). In several cases, to achieve the required extremely high ^13^C NMR chemical shift resolution, band-selective HMBC and band-selective HSQC experiments were the method of choice. For 1D measurements, 64K data points were used to yield the FID. For 2D measurements, usually 2K × 256 or 1K × 128 data points (*t*_2_ × *t*_1_) were acquired, respectively. For F_1_, linear prediction was applied to enhance the resolution. For the band-selective HSQC experiments, a digital resolution of 8–25 Hz per point was used. Most ^1^H NMR assignments were accomplished using a general knowledge of chemical shift dispersion with the aid of the hydrogen–hydrogen coupling pattern (^1^H NMR spectra). HRMS were acquired on a Thermo Scientific Q-Exactive Plus Orbitrap mass spectrometer (Thermo Fisher Scientific Inc., Budapest, Hungary) equipped with an electrospray ionization ion source in the positive ionization mode (HRESIMS). Flash chromatography was performed on a CombiFlash Rf+ Lumen instrument equipped with an integrated evaporative light-scattering detector (Teledyne Isco, Lincoln, NE, USA). RediSep stationary phases and flash columns were obtained from Teledyne Isco Inc. Preparative HPLC and preparative supercritical fluid chromatography (SFC) was performed on a JASCO SFC system (PU-4386 and PU-4086 pumps, MX-4300 dynamic mixer, CO-4060 thermostat, MD-4015 PDA detector, and BP-4340-H back-pressure regulator; JASCO International Co. Ltd., Hachioji, Tokyo, Japan) used in HPLC or SFC mode. To provide a better overview of the structural relationships between the compounds, they were numbered according to the logic of the structure elucidation, and therefore, compound numbers do not strictly follow the order of isolation (see below). During the multistep chromatographic isolation procedure, fractions were always joined according to their thin-layer chromatography fingerprints obtained with previously published solvent systems.^37^

### Plant Material

The starting material for the isolation was a commercial extract of *C. arachnoidea* roots (20 kg) purchased from Xi’an Olin Biological Technology Co., Ltd. (Xi’an, People’s Republic of China). A representative sample of the extract was deposited in the Institute of Pharmacognosy, University of Szeged, and it is available from the authors upon request.

### Extraction and Isolation

An aliquot of 5460 g of *C*. *arachnoidea* was extracted with MeOH and purified via a multistep chromatographic purification procedure to isolate compounds **1**–**10**; a detailed description of the isolation procedure is provided in the Supporting Information.

#### 14β,17α-14-Deoxypoststerone (**1**):

white solid, [α]^25^_D_ +29.3 (*c* 0.096, MeOH); ^13^C and ^1^H NMR data, see Tables 1 and 2, respectively, and Figures S1–S11, Supporting Information; HRESIMS *m*/*z* 347.22157 [M + H]^+^ (calcd for C_21_H_31_O_4_^+^ 347.22169; Δ*m*/*z* = −0.33 ppm), 369.20350 [M + Na]^+^ (calcd for C_21_H_30_O_4_Na^+^ 369.20363; Δ*m*/*z* = −0.35 ppm).

#### 14β,20(*S*)-14-Deoxy-20-hydropoststerone (**2**):

white solid, [α]^25^_D_ +11.5 (*c* 0.1, MeOH); ^13^C and ^1^H NMR data, see Tables 1 and 2, respectively, and Figures S12–S17, Supporting Information; HRESIMS *m*/*z* 349.23731 [M + H]^+^ (calcd for C_21_H_33_O_4_^+^ 349.23734; Δ*m*/*z* = −0.07 ppm), 371.21925 [M + Na]^+^ (calcd for C_21_H_32_O_4_Na^+^ 371.21928; Δ*m*/*z* = −0.08 ppm).

#### Bathoristerone (**3**):

white solid, [α]^25^_D_ +81 (*c* 0.061, MeOH); ^13^C and ^1^H NMR data, see Tables 1 and 2, respectively, and Figures S18–S23, Supporting Information; HRESIMS *m*/*z* 361.20089 [M + H]^+^ (calcd for C_21_H_29_O_5_^+^ 361.20095; Δ*m*/*z* = −0.17 ppm), 383.18272 [M + Na]^+^ (calcd for C_21_H_28_O_5_Na^+^ 383.18290; Δ*m*/*z* = −0.46 ppm).

#### 14β-14,15-Dihydrocalonysterone (**4**):

white solid, [α]^25^_D_ +123.7 (*c* 0.130, MeOH); ^13^C and ^1^H NMR data, see Tables 1 and 2, respectively, and Figures S24–S28, Supporting Information; HRESIMS *m*/*z* 479.30065 [M + H]^+^ (calcd for C_27_H_43_O_7_^+^ 479.30033; Δ*m*/*z* = 0.67 ppm), 501.28245 [M + Na]^+^ (calcd for C_27_H_42_O_7_Na^+^ 501.28227; Δ*m*/*z* = 0.35 ppm).

#### 14-Deoxydacryhainansterone (**5**):

white solid, [α]^25^_D_ +58 (*c* 0.086, MeOH); ^13^C and ^1^H NMR data, see Tables 1 and 2, respectively, and Figures S29–S35, Supporting Information; HRESIMS *m*/*z* 447.31055 [M + H]^+^ (calcd for C_27_H_43_O_5_^+^ 447.31050; Δ*m*/*z* = 0.11 ppm), 469.29238 [M + Na]^+^ (calcd for C_27_H_42_O_5_Na^+^ 469.29245; Δ*m*/*z* = −0.14 ppm).

#### 22-Oxodacryhainansterone (**6**):

white solid, [α]^25^_D_ +51.3 (*c* 0.170, MeOH); ^13^C and ^1^H NMR data, see Tables 1 and 3, respectively, and Figures S36–S40, Supporting Information; HRESIMS *m*/*z* 461.28981 [M + H]^+^ (calcd for C_27_H_41_O_6_^+^ 461.28977; Δ*m*/*z* = 0.10 ppm), 483.27175 [M + Na]^+^ (calcd for C_27_H_40_O_6_Na^+^ 483.27171; Δ*m*/*z* = 0.08 ppm).

#### 14-Deoxy-25-hydroxydacryhainansterone (**7**):

white solid, [α]^25^_D_ +123.4 (*c* 0.013, MeOH); ^13^C and ^1^H NMR data, see Tables 1 and 3, respectively, and Figures S41–S47, Supporting Information; HRESIMS *m*/*z* 463.30548 [M + H]^+^ (calcd for C_27_H_43_O_6_^+^ 463.30542; Δ*m*/*z* = 0.14 ppm), 485.28733 [M + Na]^+^ (calcd for C_27_H_42_O_6_Na^+^ 485.28736; Δ*m*/*z* = −0.06 ppm).

#### 14-Deoxy-20-hydroxyecdysone (**8**):

white solid, [α]^25^_D_ +42.2 (*c* 0.095, MeOH); ^13^C and ^1^H NMR data, see Tables 1 and 3, respectively, and Figures S48–S53, Supporting Information; HRESIMS *m*/*z* 465.32119 [M + H]^+^ (calcd for C_27_H_45_O_6_^+^ 465.32107; Δ*m*/*z* = 0.27 ppm), 487.30308 [M + Na]^+^ (calcd for C_27_H_44_O_6_Na^+^; Δ*m*/*z* = 0.14 ppm).

#### 5α-14-Deoxy-20-hydroxyecdysone (**9**):

white solid, [α]^25^_D_ +28.1 (*c* 0.096, MeOH); ^13^C and ^1^H NMR data, see Tables 1 and 3, respectively, and Figures S54–S62, Supporting Information; HRESIMS *m*/*z* 465.32125 [M + H]^+^ (calcd for C_27_H_45_O_6_^+^ 465.32107; Δ*m*/*z* = 0.40 ppm), 487.30322 [M + Na]^+^ (calcd for C_27_H_44_O_6_Na^+^ 487.30301; Δ*m*/*z* = 0.43 ppm).

#### 14β-14-Deoxy-20-hydroxyecdysone (**10**):

white solid, [α]^25^_D_ −3.8 (*c* 0.106, MeOH); ^13^C and ^1^H NMR data, see Tables 1 and 3, respectively, and Figures S63–S69, Supporting Information; HRESIMS *m*/*z* 465.32124 [M + H]^+^ (calcd for C_27_H_45_O_6_^+^ 465.32107; Δ*m*/*z* = 0.37 ppm), 487.30306 [M + Na]^+^ (calcd for C_27_H_44_O_6_Na^+^ 487.30301; Δ*m*/*z* = 0.10 ppm).

Calonysterone (**11**) and dacryhainansterone (**12**) were obtained using centrifugal partition chromatography from the same source extract used for compounds **1**–**10** as previously reported.^38^ Shidasterone (**13**) and ajugasterone C (**14**) were isolated from a food supplement containing a *C. arachnoidea* extract of unknown origin.^5^ 5α-2-Deoxyintegristerone A (**15**) was isolated from *Silene italica* ssp. *nemoralis*.^39^

### Preparation of Semisynthetic Ecdysteroid Derivatives

Poststerone (20*E*)-oxime (**16**) was prepared using semisynthesis from 20E through poststerone according to a previously published procedure.^40^ Briefly, an aliquot of 380 mg of poststerone (1.04 mmol) was dissolved in 20 mL of pyridine. Then, 110 mg (1.57 mmol) of hydroxylamine hydrochloride was added, and the mixture was stirred for 25 min at room temperature. Subsequently, the solution was cooled to 0 °C in an ice bath and neutralized with an ethyl alcohol solution of potassium hydroxide (88.2 mg, 1.57 mmol). The solution was then evaporated to dryness on a rotary evaporator, water (50 mL) was added to the dry residue, and the products were extracted with ethyl acetate (3 × 50 mL). The organic fractions were combined, dried over Na_2_SO_4_, and filtered. Then, the solution was evaporated to dryness under reduced pressure, and the residue was subjected to preparative RP-HPLC purification (Kinetex XB-C18, 21 × 250 mm, 5 μm; 23% acetonitrile (CH_3_CN)(aq), flow rate: 15 mL/min), which afforded poststerone (20*E*)-oxime (**16**) in 75% yield (297 mg).

20E (6*Z*)-oxime (**17**) and 20E (6*E*)-oxime (**18**) were prepared from 20E by slightly modifying the procedure previously reported by Galyautdinov et al.^41^ Briefly, an aliquot of 1 g of hydroxylamine hydrochloride (14.39 mmol) was dissolved in 15 mL of ethanol. The solution was neutralized with an ethyl alcoholic solution of potassium hydroxide (807.4 mg, 14.39 mmol), and subsequently, 1 g of 20E was added. The reaction mixture was stirred for 14 days at the boiling point. Subsequently, silica gel (4 g) was added to the solution, which was then evaporated to dryness on a rotary evaporator to prepare the sample for dry loading normal-phase flash chromatographic purification. The separation was performed on a 24 g silica column (flow rate 35 mL/min, run time: 30 min) with a gradient of dichloromethane (A) and MeOH (B), from 0% to 35% of solvent B in A. The eluted product mixture consisted of 20E (6*Z*)-oxime (**17**) and 20E (6*E*)-oxime (**18**) in an approximately 1:2 ratio. Subsequently, the ecdysteroid oxime isomers were separated using preparative RP-HPLC (Kinetex XB-C18, 21 × 250 mm, 5 μm; 15% CH_3_CN(aq), flow rate: 15 mL/min), affording 20E (6*Z*)-oxime (**17**) in 20% yield (202 mg) and 20E (6*E*)-oxime (**18**) in 46% yield (478 mg).

The 2,3-dioxolane-substituted compound **19**,^12^ 9α,20E (**20**), and 5α-9α,20E (**21**)^42^ were available from previous work on semisynthetic derivatives of 20E.

### EcR Binding Assay

Tritiated PonA ([^3^H]PonA; 3.5 TBq/mmol) was custom synthesized by American Radiolabeled Chemicals, Inc. (St. Louis, MO, USA). A stock solution of [^3^H]PonA (30 000 dpm/μL, ∼0.1 μM) was prepared in 70% aqueous EtOH. Cold PonA and 20E were obtained from Enzo Biochem Inc. (New York, NY, USA) and Sigma-Aldrich Co. (St. Louis, MO, USA), respectively. Tebufenozide was obtained from stock samples. All test compounds were dissolved in DMSO. An Sf-9 cell line was cultured in EX-CELL 420 medium (SAFC Biosciences, Inc., Lenexa, KS, USA) supplemented with 10% fetal bovine serum.

The binding assay was performed as described previously with some modifications.^43,44^ To an Sf-9 cell suspension (400 μL; (2.0–3.0) × 10^6^ cells/mL) in a disposable culture tube (12 mm × 75 mm) was added a test compound solution (1 μL), followed by a [^3^H]PonA solution (2 μL, 60 000 dpm). After incubation at 25 °C for 30 min, the cell suspension was diluted with water (3 mL) and filtered through a glass fiber filter GF/B (25 mm; Whatman plc, Maidstone, UK). The filter was washed with water (2 × 3 mL), dried, and transferred to a glass vial. To the vial were added 3 mL of Insta-Gel Plus (PerkinElmer Inc., Waltham, MA, USA), and the radioactivity was measured with an LSC-8000 counter (Aloka, Tokyo, Japan). DMSO and PonA (final concentration: 2.7 μM) were used to determine the total and nonspecific binding, respectively. The test compounds were first screened at 25 μM, and the compounds with an inhibition rate of ≥50% were subjected to concentration–response experiments. The 50% inhibition concentration for [^3^H]PonA binding [IC_50_ (M)] was determined using probit analysis,^45^ and the logarithm of its reciprocal, pIC_50_, was used as the index of the binding affinity.

### Reporter Gene Assay

The reporter gene assay was performed in Sf-9 cells according to a previously reported method.^46^ Briefly, a reporter plasmid was constructed from pBmbA/hsp27/firefly luciferase (hsp27/Fluc), in which seven repeats of hsp27 ecdysone response element were inserted. This plasmid was a kind gift from Dr. Luc Swevers (Institute of Biosciences & Applications, National Center for Scientific Research “Demokritos”, Athens, Greece). The plasmid was amplified in *Escherichia coli* and purified using a QIAGEN plasmid midi kit (QIAGEN GmbH, Hilden, Germany). A cell suspension (approximately 2.0 × 10^6^ cells/mL; 400 μL) was added to each well of a 24-well plate, and it was incubated for 1 h. Then, 200 μL of a mixture of plasmids and TransFast transfection reagent (Promega; Madison, WI, USA) was added to each well. After incubating for 24 h, 6 μL of a solution of test compound (1% v/v) was added and incubated. After 48 h, the medium in each well was removed with a pipet. Cells were lysed by adding 100 μL/well of Passive Lysis Buffer and incubating for 15 min at 25 °C. The lysate (20 μL) was transferred to a 96-well microplate for the luciferase assay. Chemical luminescence was measured using a 96-microplate GLOMAX luminometer (Promega), according to the manufacturer’s instructions.

## Supporting Information Available

Detailed isolation procedure for compounds **1**–**10** (PDF)
Characteristic 1D and 2D NMR spectra for compounds **1** (Figures S1–S11), **2** (Figures S12–S17), **3** (Figures S18–S23), **4** (Figures S24–S28), **5** (Figures S29–S35), **6** (Figures S36–S40), **7** (Figures S41–S47), **8** (Figures S48–S53), **9** (Figures S54–S62), and **10** (Figures S63–S69) (PDF)
