# Supplementary material for: A Commercial Extract of Cyanotis arachnoidea Roots as a Source of Unusual Ecdysteroid Derivatives with Insect Hormone Receptor Binding Activity
Source: J Nat Prod. 2021 Jun 18;84(7):1870–81. doi: 10.1021/acs.jnatprod.0c01274 (PMC8314276; doi:10.1021/acs.jnatprod.0c01274)
Supplement: Supplementary file 1 — np0c01274_si_001.pdf [file np0c01274_si_001.pdf]

Supporting information for

Commercial extracts of *Cyanotis arachnoidea* as a Source of Unusual Ecdysteroid Derivatives with Insect Hormone Receptor Binding Activity

Gábor Tóth,* Ibolya Herke, Tamás Gáti, Máté Vágvölgyi, Róbert Berkecz, Lyudmila V. Parfenova, Minori Ueno, Taiyo Yokoi, Yoshiaki Nakagawa, and Attila Hunyadi*

*: Corresponding authors, email: hunyadi.attila@szte.hu (A.H.) and drtothgabor@t-online.hu (G.T.)

Detailed Description of the Isolation Procedure

An aliquot of 5460 g of CA was extracted with 15.5 L of MeOH in a percolator and evaporated to dryness under reduced pressure to yield 1398 g of dry residue. This was divided into two equal parts, each of which was fractionated by column chromatography as follows, and the corresponding fractions eluted with the same solvent were subsequently joined. The ca. 700 g of extract was adsorbed on 2100g of silica and applied on top of a column of 1500 g of silica. Elution was performed by CH_2Cl_2 (20 L) followed by a stepwise gradient of CH_2Cl_2 – MeOH mixtures (97:3, 10 L; 95:5, 20 L; 93:7, 22.5 L; 85:15, 25 L). The combined fractions eluted with 95:5 (*F1*), 93:7 (*F2*), and 85:15 (*F3*) were evaporated to dryness to yield 210, 415.8, and 406 g of dry residues, respectively.

Fraction F1 was adsorbed to 840 g of Celite 545 and layered on top of a 2540 g silica column. Gradient elution was performed, and a total of 80 fractions (*F1.1-80*) of 2.5 L each were collected, using *c*-hexane (*F1.1*), *c*-hexane – ethyl acetate (6:2, *F1.2-3*), (6:3, *F1.4-5*), (6:4, *F1.6-9*), (6:5, *F1.10-19*), and *c*-hexane – ethyl acetate – ethanol (60:50:2, *F1.20-30*), (60:50:5, *F1.31-49*), (60:50:10, *F1.50-66*), (60:50:20, *F1.67-80*) solvent systems. Fractions *F1.26-51* were combined evaporated to dryness to yield

130.8 g of dry residue. This was adsorbed on 327 g of RediSep Rf C_{18} (40-60 um, Teledyne Isco Inc, Lincoln, NE, USA) and applied on top of 400 g of the same stationary phase. Gradient elution was performed, and a total of 35 fractions (*F1.RP1-31*) were collected, using H_2O – acetonitrile (8:2, *F1.RP1-23*), (7:3, *F1.RP24-31*), and (6:4, *F1.RP32-35*) solvent systems. The dry residue of fraction *F1.RP4* (5.6 g) was purified by flash chromatography with dry loading technique on a 275 g RediSep C_{18} Gold column (flow rate: 125 mL/min, fraction volume: 300 mL, run time: 80 min) eluted by a gradient of aqueous MeOH from 25 to 60%. Fractions *11-13* of this separation were then further purified by flash chromatography on an 80 g RediSep Silica Gold column (flow rate 60 mL/min, fraction volume: 300 mL, run time: 70 min) with a gradient of CH_2Cl_2 (A) and 97% ethanol (B), from 0 to 6 % of solvent B in A. Fractions 8-11 of this separation were joined, and preparative NP-HPLC (Luna Silica column, 21x250 mm, 5 μm, flow rate: 15 mL/min, solvent: CH_2Cl_2 – *i*-PrOH – H_2O, 125:15:1) was used to obtain compounds **1** (14.2 mg) and **3** (8.7 mg). From the RP-flash separation of *F1.RP4*, fractions 14-15 were purified by NP-flash chromatography using the previously described conditions, and preparative NP-HPLC (Luna Silica column, 21x250 mm, 5 μm, flow rate: 15 mL/min, solvent: *c*-hexane – *i*-PrOH – H_2O, 100:40:3) of fractions 3-5 afforded compound **2** (59.6 mg).

The combined dry residue of fractions *F1.RP26-31* (19.6 g) was divided into two, and each was further fractionated by flash chromatography on an 80g RediSep Silica Gold column with dry loading technique (flow rate 60 mL/min, fraction collection based on detector signal, run time: 70 min) with a gradient of CH_2Cl_2 (A) and *i*-PrOH (B), from 0

to 30 % of solvent B in A. The corresponding fractions of these two separations were joined into 5 sub-fractions (*F1.RP26-31.NP1-5*). *F1.RP26-31.NP2* (3.06 g) was further purified by flash chromatography on a 24 g RediSep Silica Gold column (flow rate 35 mL/min, fraction collection based on detector signal, run time: 75 min) with a gradient of *n*-hexane - CH_2Cl_2 (1:1; A) and 96% ethanol (B), from 3 to 10 % of solvent B in A. The main component of fractions 31-66 was purified by semi-preparative NP-HPLC on a Zorbax sil column (9x250 mm, 5µm; CH_2Cl_2 – *i*-PrOH – H_2O, 125:15:1, flow: 2.5 mL) to obtain compound **5** (69.5 mg).

Fraction *F1.RP26-31.NP5* (4.16 g) was adsorbed on 21.5 g silica for dry loading, and purified by flash chromatography on an 80 g RediSep Silica Gold column (flow rate 60 mL/min, fraction collection based on detector signal, run time: 75 min) with a gradient of *n*-hexane – CH_2Cl_2 (1:1; A) and 96% ethanol (B), using the following gradient steps: from 0 to 5 % (20 min), then to 10% (55 min), then to 30% (75 min) of solvent B in A. Fractions 55-65 were subjected to semi-preparative RP-HPLC on a Zorbax ODS column (isocratic 45% aqueous ACN, flow rate 2.5 mL/min) to afford compound **6** (60.4 mg).

Fraction *F2* (415.8 g) was adsorbed on 1000 g of silica, applied on top of a column of 2000 g of silica, and further fractionated with a stepwise gradient of *n*-hexane – ethyl acetate – ethanol, 60:40:0, 60:50:0, 60:50:2, 60:50:5, 60:50:10, 60:50:20, and 60:50:30, collecting 350 fractions of 500 mL each. Fractions 115-153 (78.9 g) were joined, adsorbed on 300 g of RediSep C_{18}, applied on a column of 500 g of RediSep C_{18}, and purified with a stepwise gradient of aqueous MeOH from 10% to 40%,

collecting 127 fractions of 200 mL each. Fractions 91-105 (10.4 g), 106-115 (7.7 g), and 116-127 (4.9 g) were separated one-by-one by flash chromatography on a 330 g RediSep Silica Gold column (flow rate 170 mL/min, fraction volume: 300 mL, run time: 60 min) with a gradient of CH_2Cl_2 (A) and 99% MeOH(aq) (B), 4% to 10% B in A, and corresponding fractions of each separation were joined (*F2.NP1-11*). Fraction *F2NP6* (0.9 g) was purified by preparative SFC on a Luna Silica column (21x250 mm, 5µm; 25% EtOH in CO_2, flow rate: 15 mL/min) to afford compound **4** (10.4 mg). Fraction *F2NP7* (2.5 g) was purified by preparative RP-HPLC (Kinetex XB-C18, 21x250 mm, 5µm; 23% ACN(aq), flow rate: 15 mL/min), and subsequently by preparative SFC (Luna Silica, 21x250 mm, 5µm; 20% EtOH in CO_2, flow rate: 15 mL/min) to afford compounds **7** (8.6 mg), **8** (36.0 mg), and **9** (71.2 mg).

Fraction *F2NP9* (1.8 g) was adsorbed on 40 g of Celite to use dry loading and purified by flash chromatography on an 80 g RediSep Silica Gold column (flow rate: 60 mL/min) with a gradient of n-hexane – EtOAc, 7:3 (A), and 66% EtOH(aq) (B), 2.5% to 4% in 90 min, and peak collection was applied. Subsequent purification of the main peak by preparative SFC (Luna Silica, 21x250 mm, 5µm; 20% EtOH in CO_2, flow rate: 15 mL/min) afforded compound **10** (77.2 mg).
