# Supplementary material for: A Commercial Extract of Cyanotis arachnoidea Roots as a Source of Unusual Ecdysteroid Derivatives with Insect Hormone Receptor Binding Activity
Source: J Nat Prod. 2021 Jun 18;84(7):1870–81. doi: 10.1021/acs.jnatprod.0c01274 (PMC8314276; doi:10.1021/acs.jnatprod.0c01274)

Supporting information for

Commercial extracts of *Cyanotis arachnoidea* as a Source of Unusual Ecdysteroid Derivatives with Insect Hormone Receptor Binding Activity

Gábor Tóth, Ibolya Herke, Tamás Gáti, Máté Vágvölgyi, Róbert Berkecz, Lyudmila V. Parfenova, Minori Ueno, Taiyo Yokoi, Yoshiaki Nakagawa, and Attila Hunyadi**

*: Corresponding authors, email: hunyadi.attila@szte.hu (A.H.) and drtothgabor@t-online.hu (G.T.)

List of Figures

		page
S1-11	Characteristic NMR spectra of compound **1**	2-12
S12-17	Characteristic NMR spectra of compound **2**	13-18
S18-23	Characteristic NMR spectra of compound **3**	19-24
S24-28	Characteristic NMR spectra of compound **4**	25-29
S29-35	Characteristic NMR spectra of compound **5**	30-36
S36-40	Characteristic NMR spectra of compound **6**	37-41
S41-47	Characteristic NMR spectra of compound **7**	42-28
S48-53	Characteristic NMR spectra of compound **8**	49-54
S54-62	Characteristic NMR spectra of compound **9**	55-63
S63-69	Characteristic NMR spectra of compound **10**	64-70

S1 Compound **1**

^{1}H 500 MHz

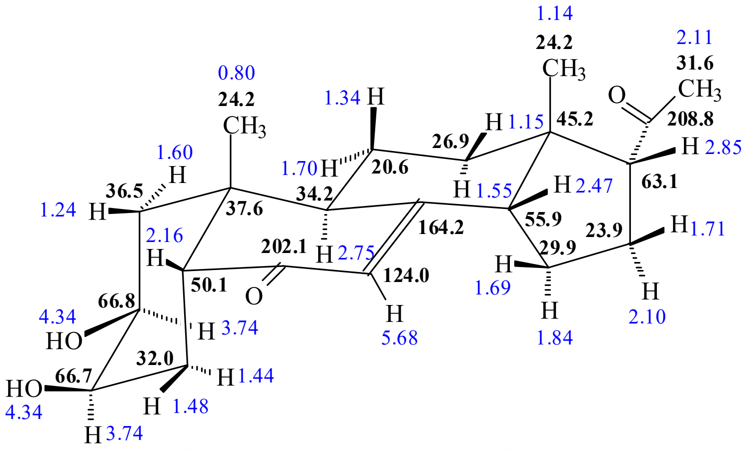

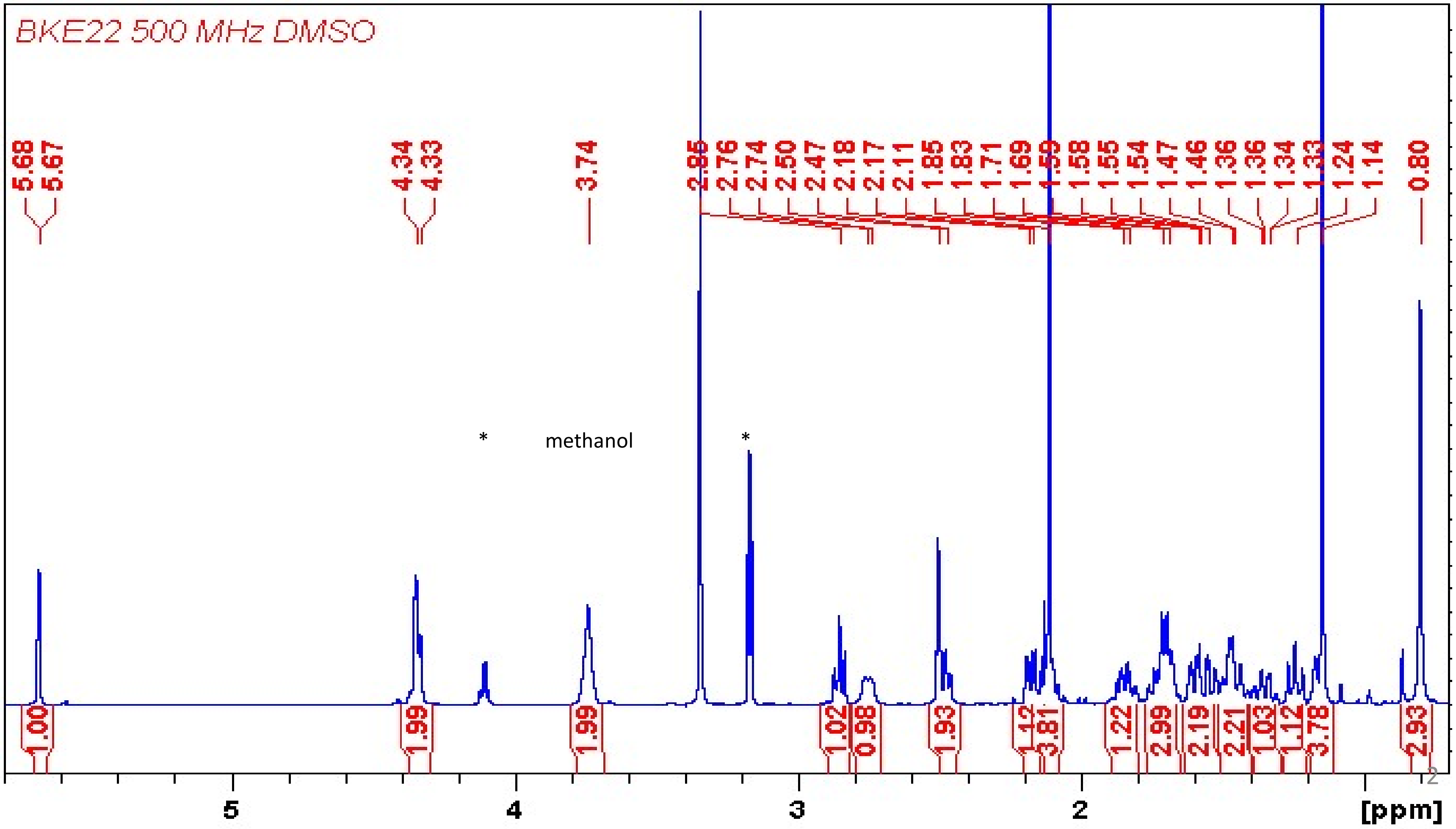

S2 Compound **1**

^{1}H,^{1}H-COSY   500 MHz

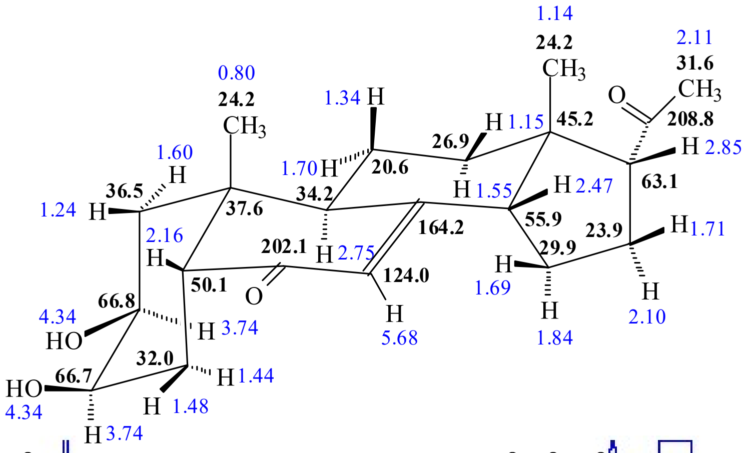

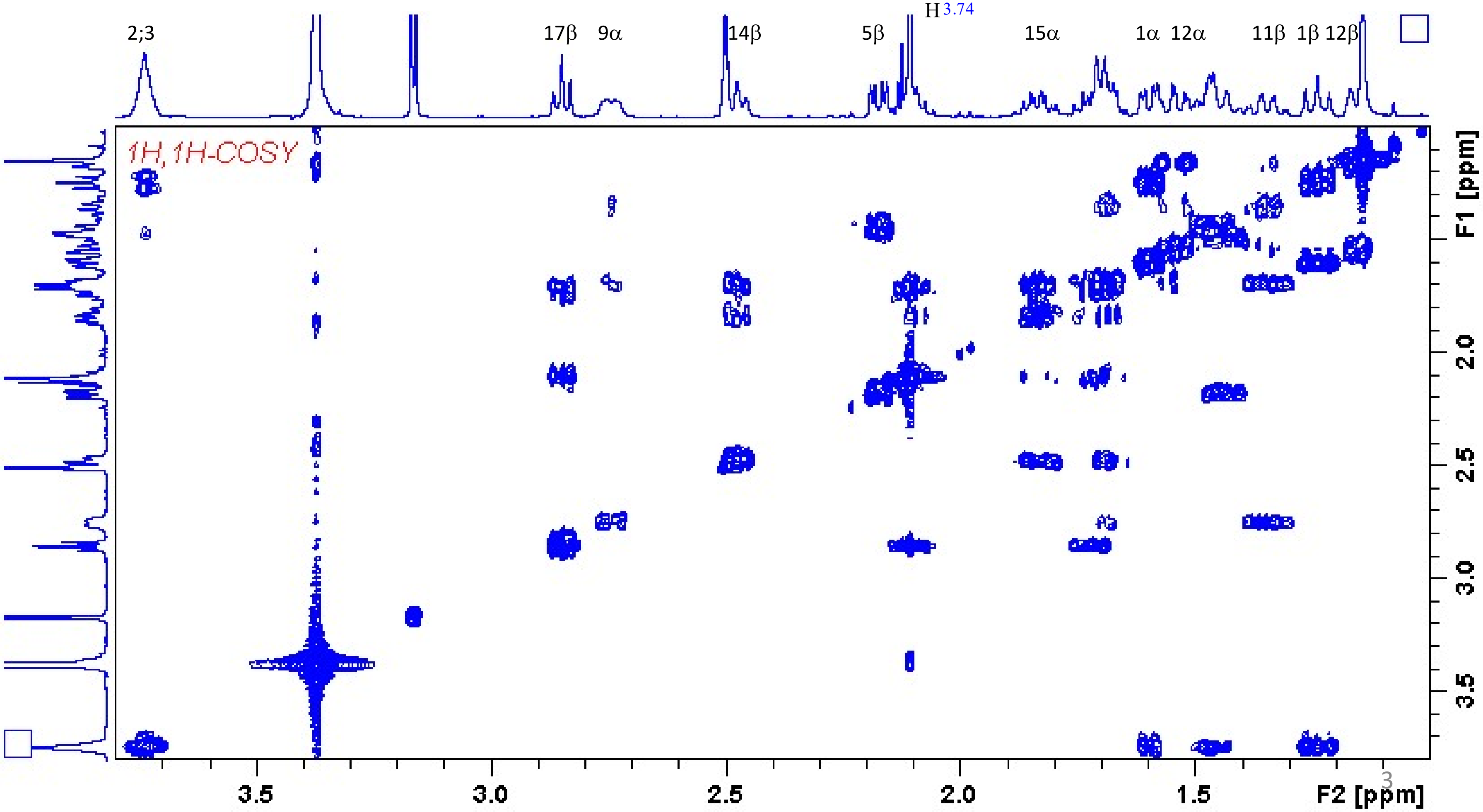

S3 Compound **1**

Identification of spin-systems by selTOCSY on H-7 and Hβ-17

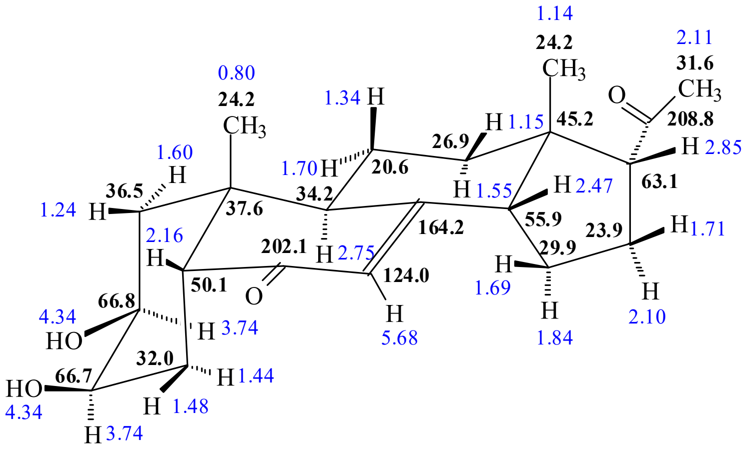

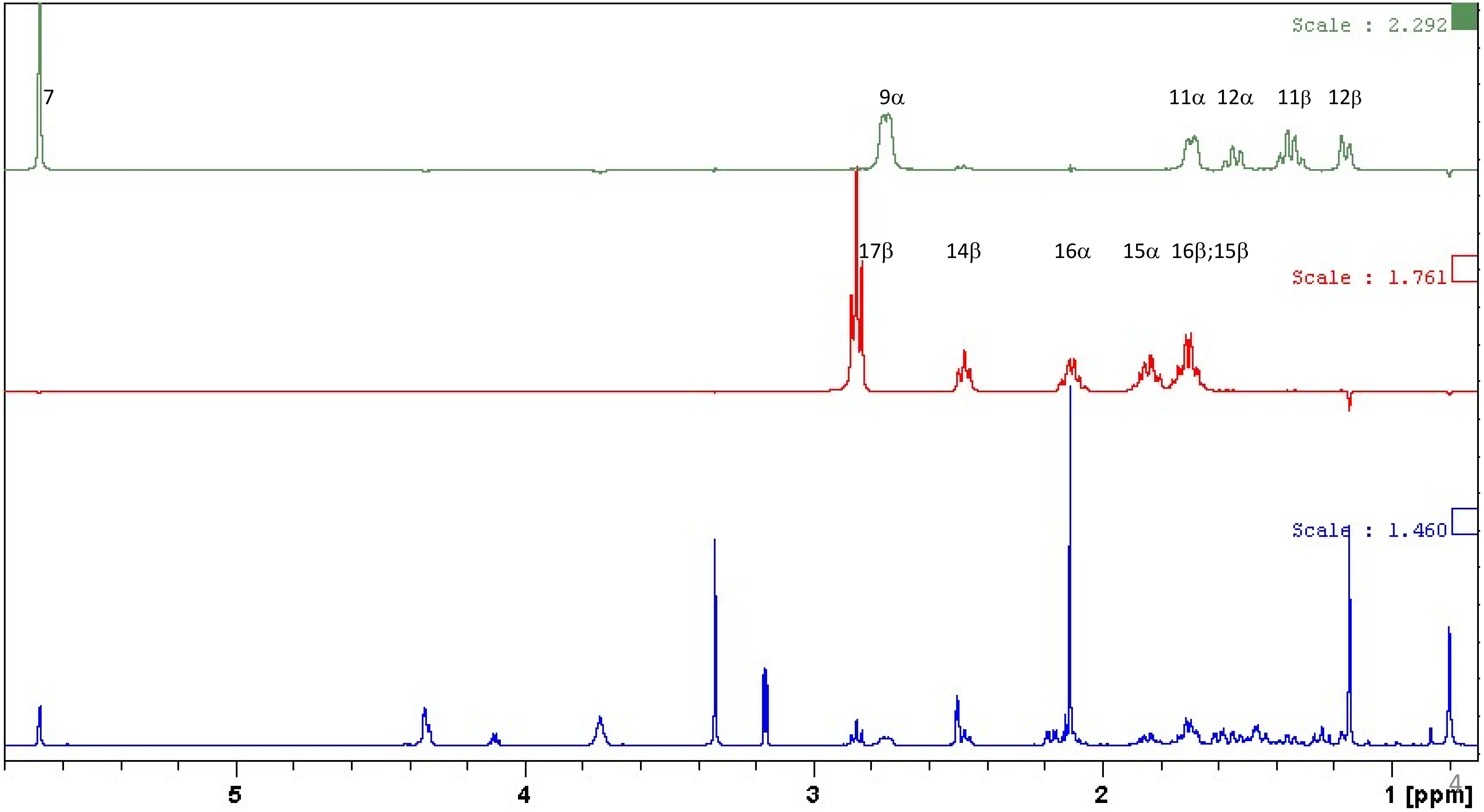

S4 Compound **1**

Steric proximities detected by selROE on Me-19, Me-18 and Hβ-14

Supporting the β positions of H-5, H-14 and H-17 atoms

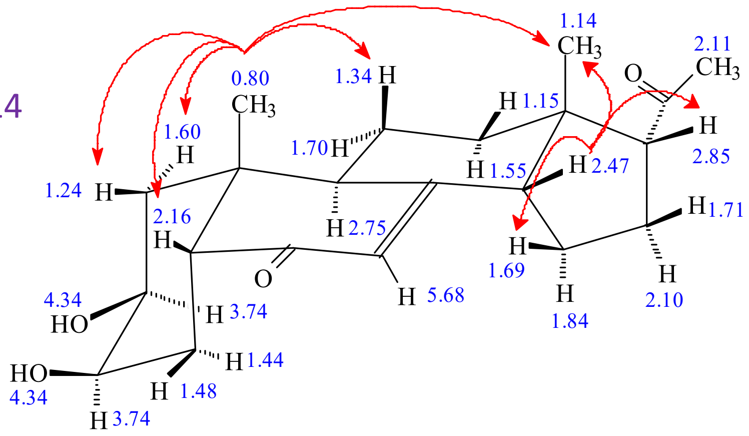

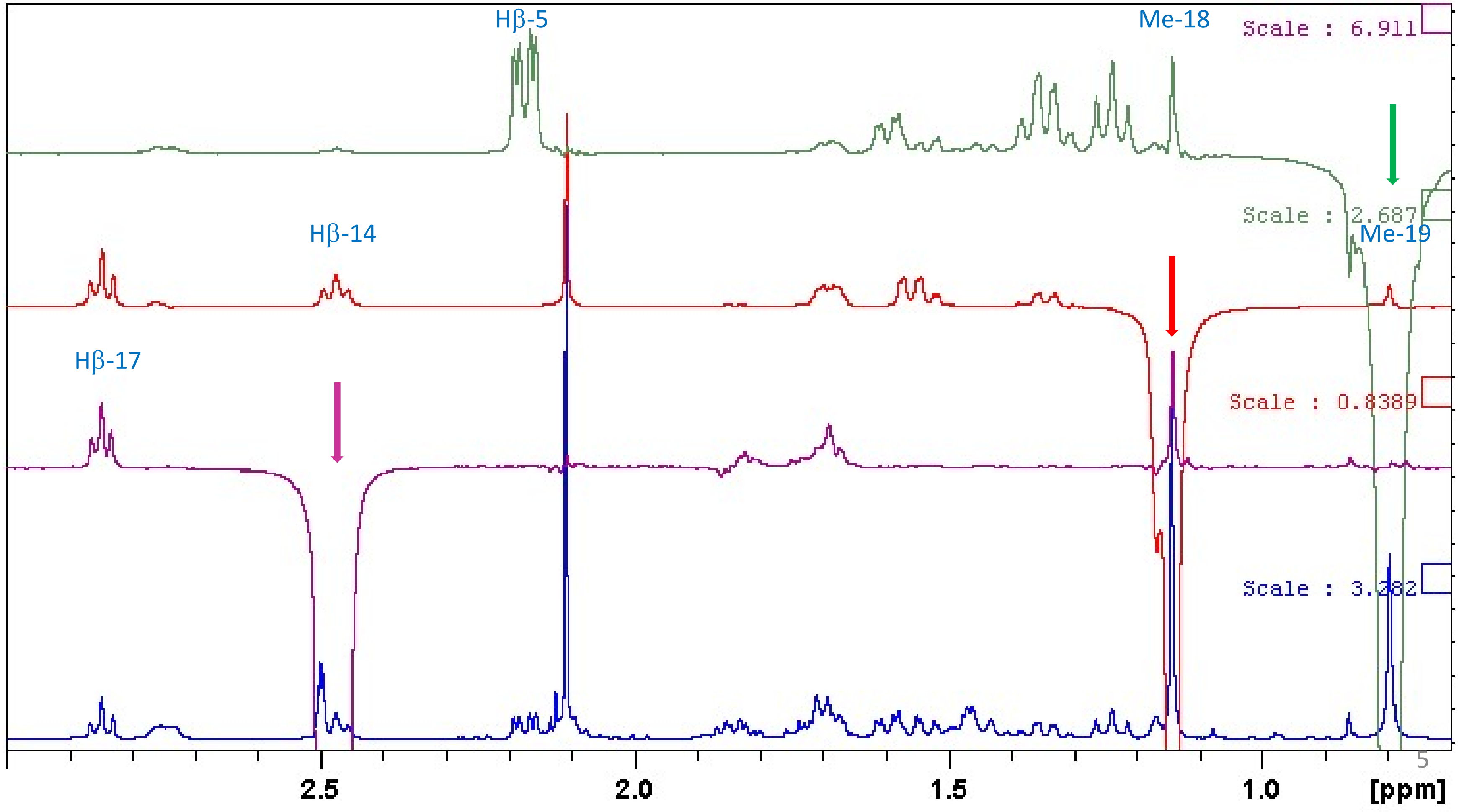

S5 Compound **1**

Steric proximities detected by selROE on Hβ-17 and Hα-9

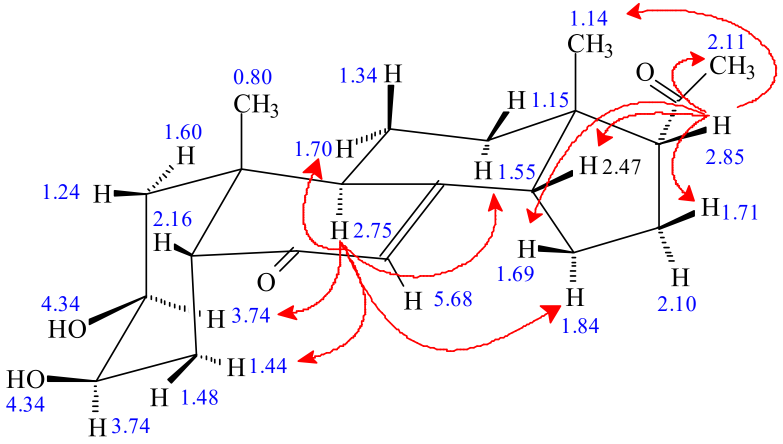

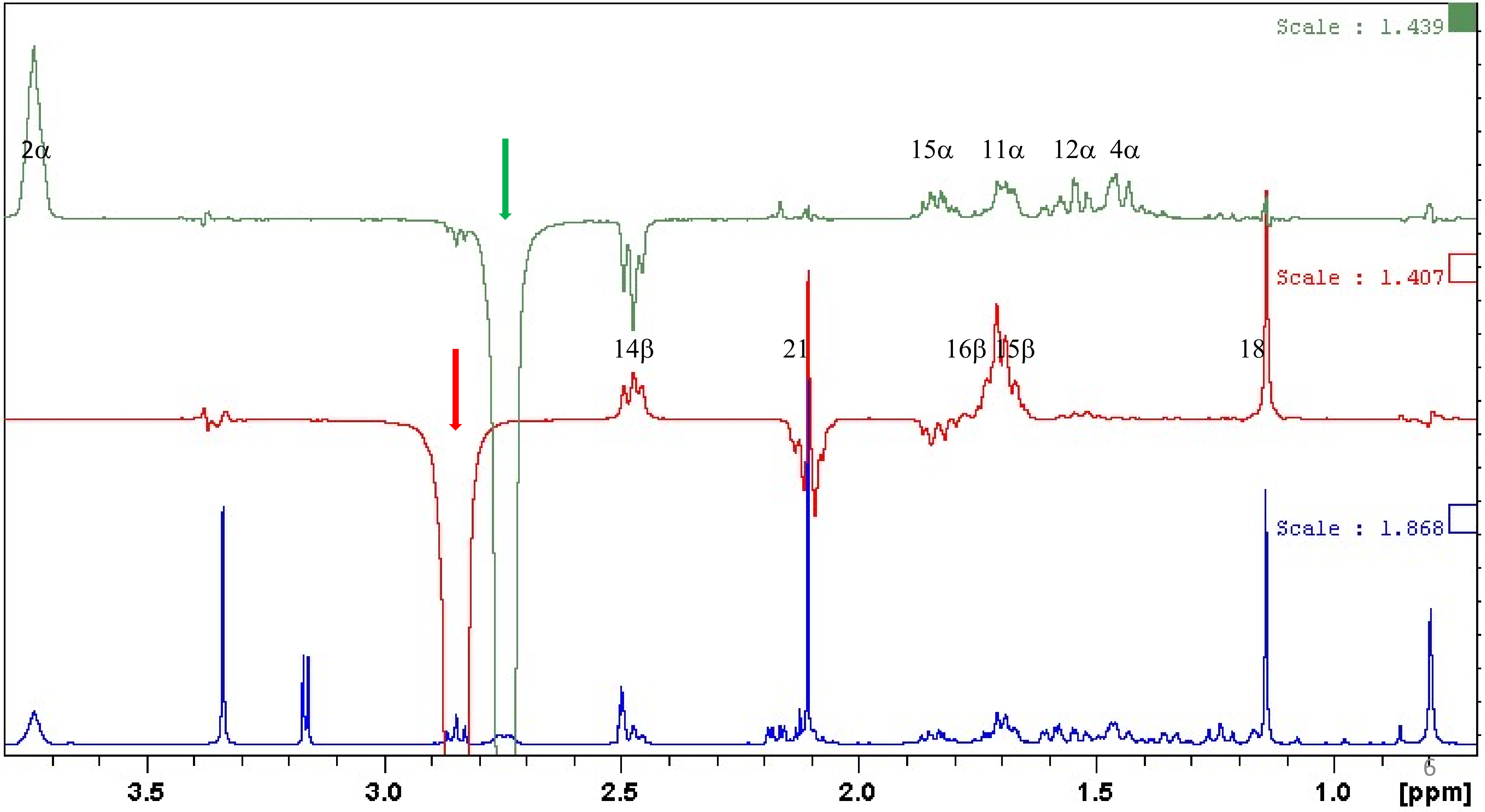

S6 Compound **1**

DeptQ 125 MHz

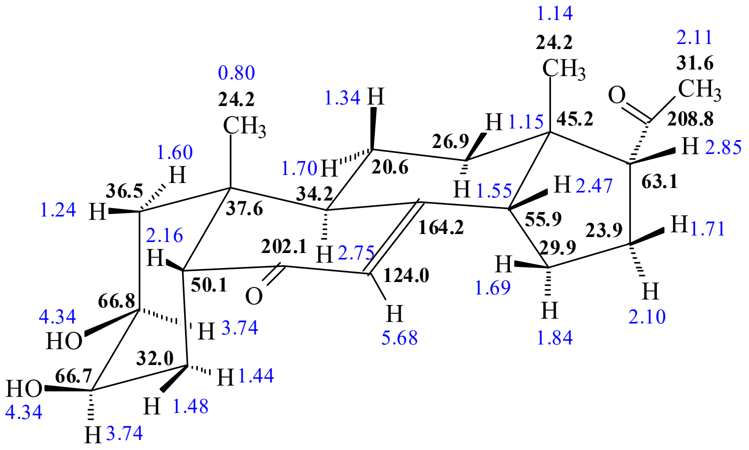

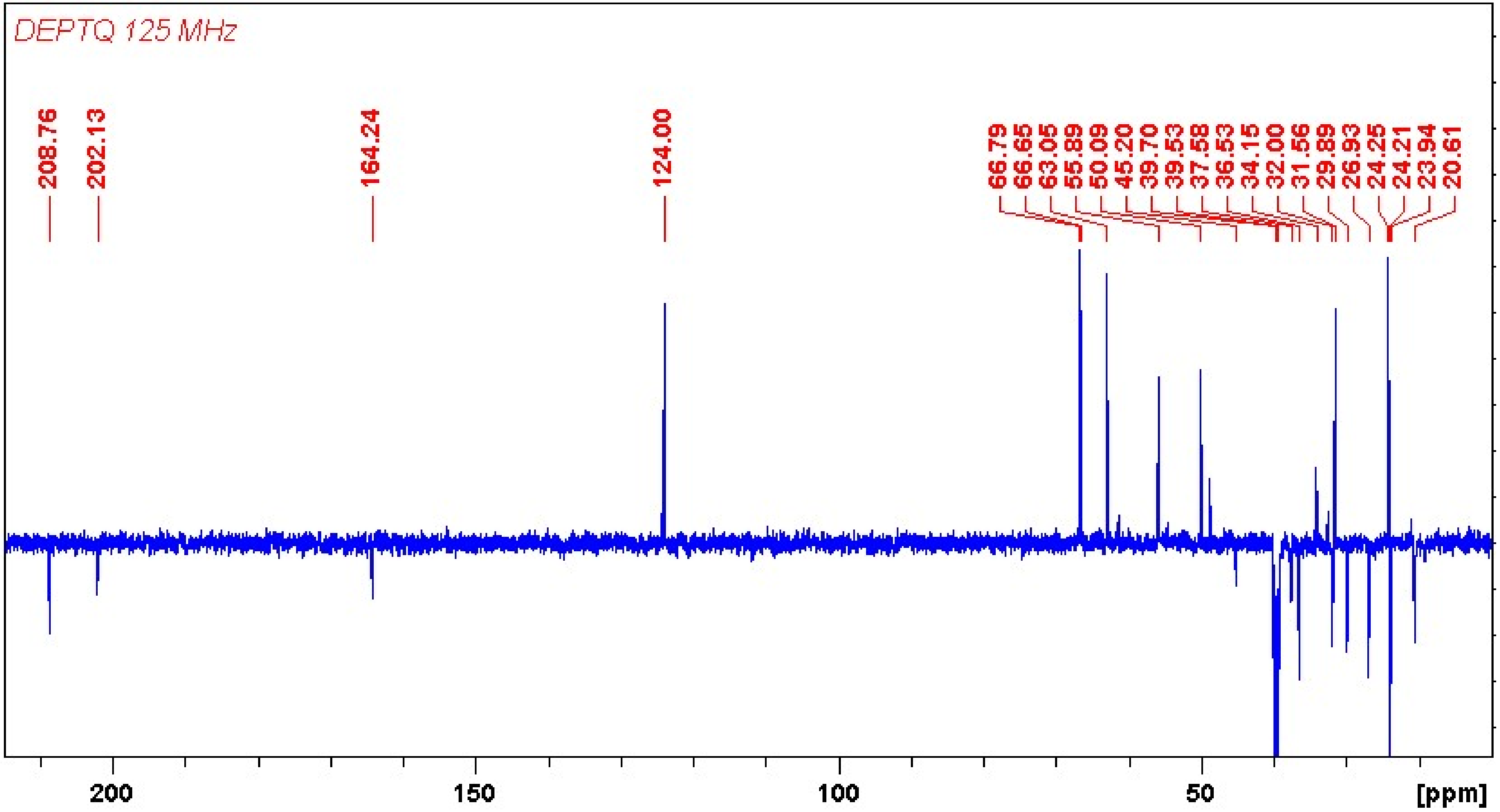

S7 Compound **1**

edHSQC

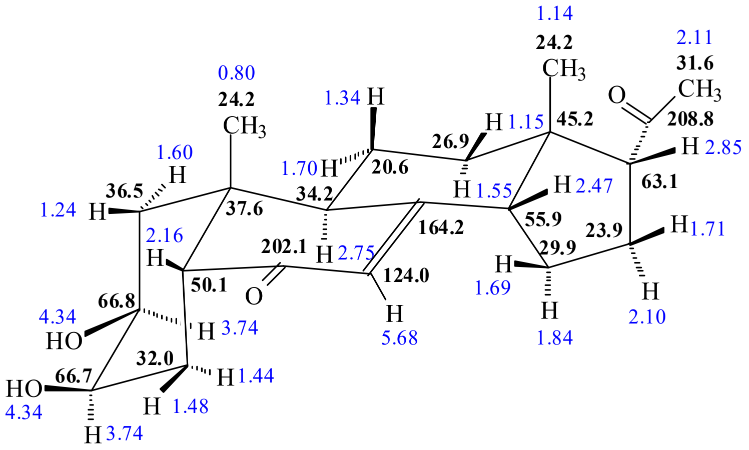

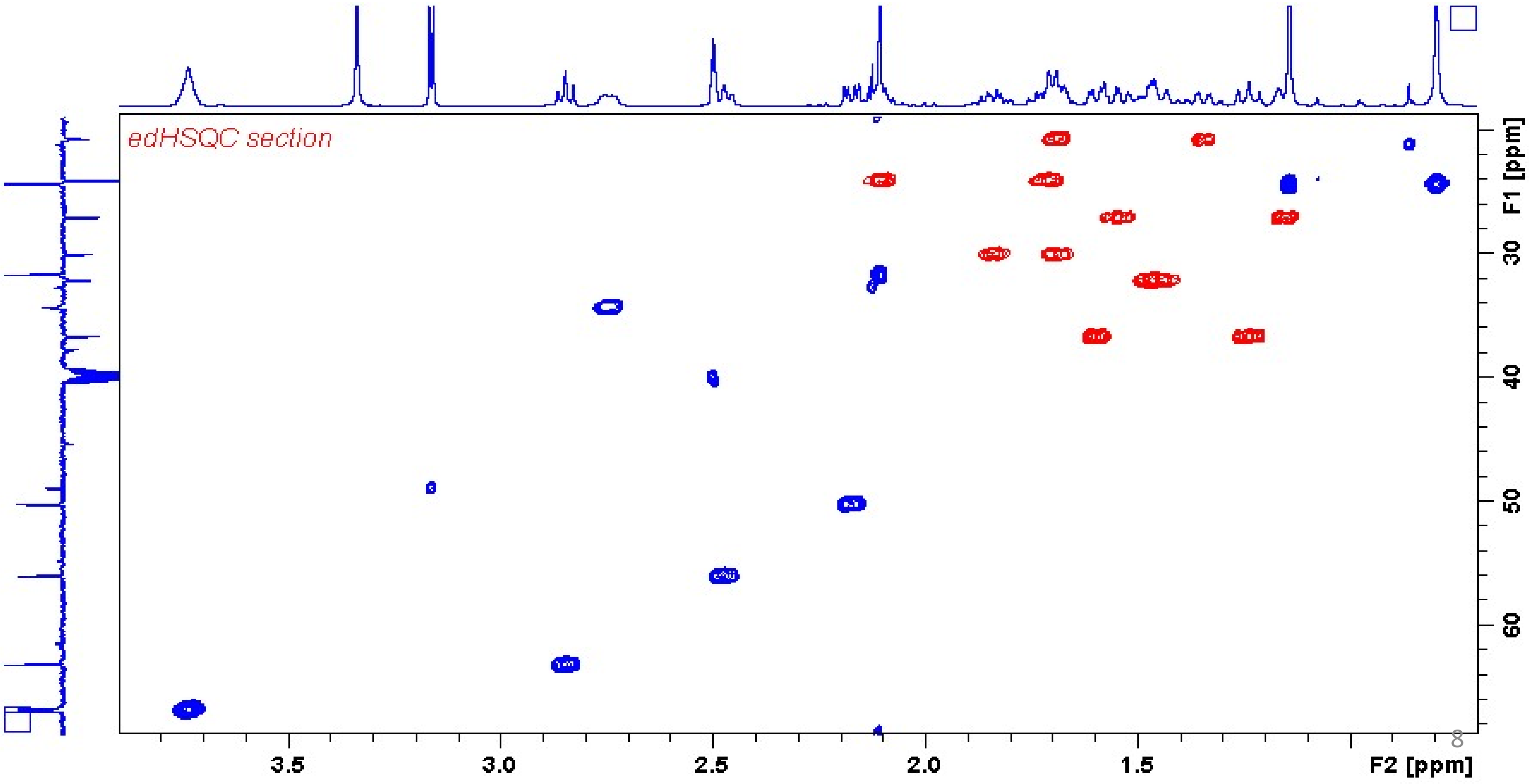

S8 Compound **1**

edHSQC + selROE on Me-19

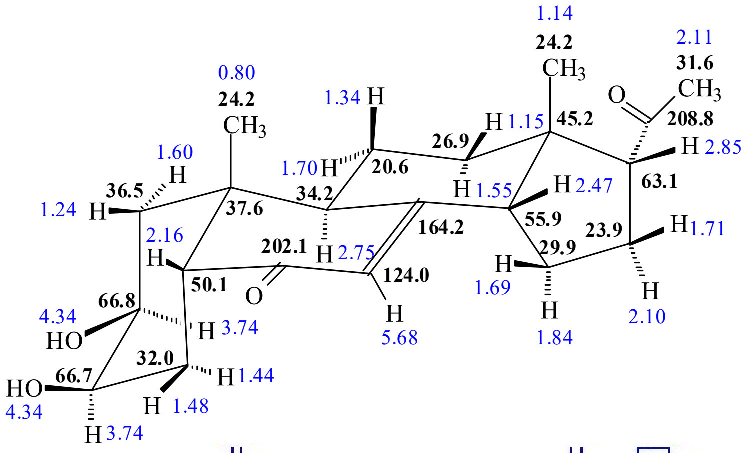

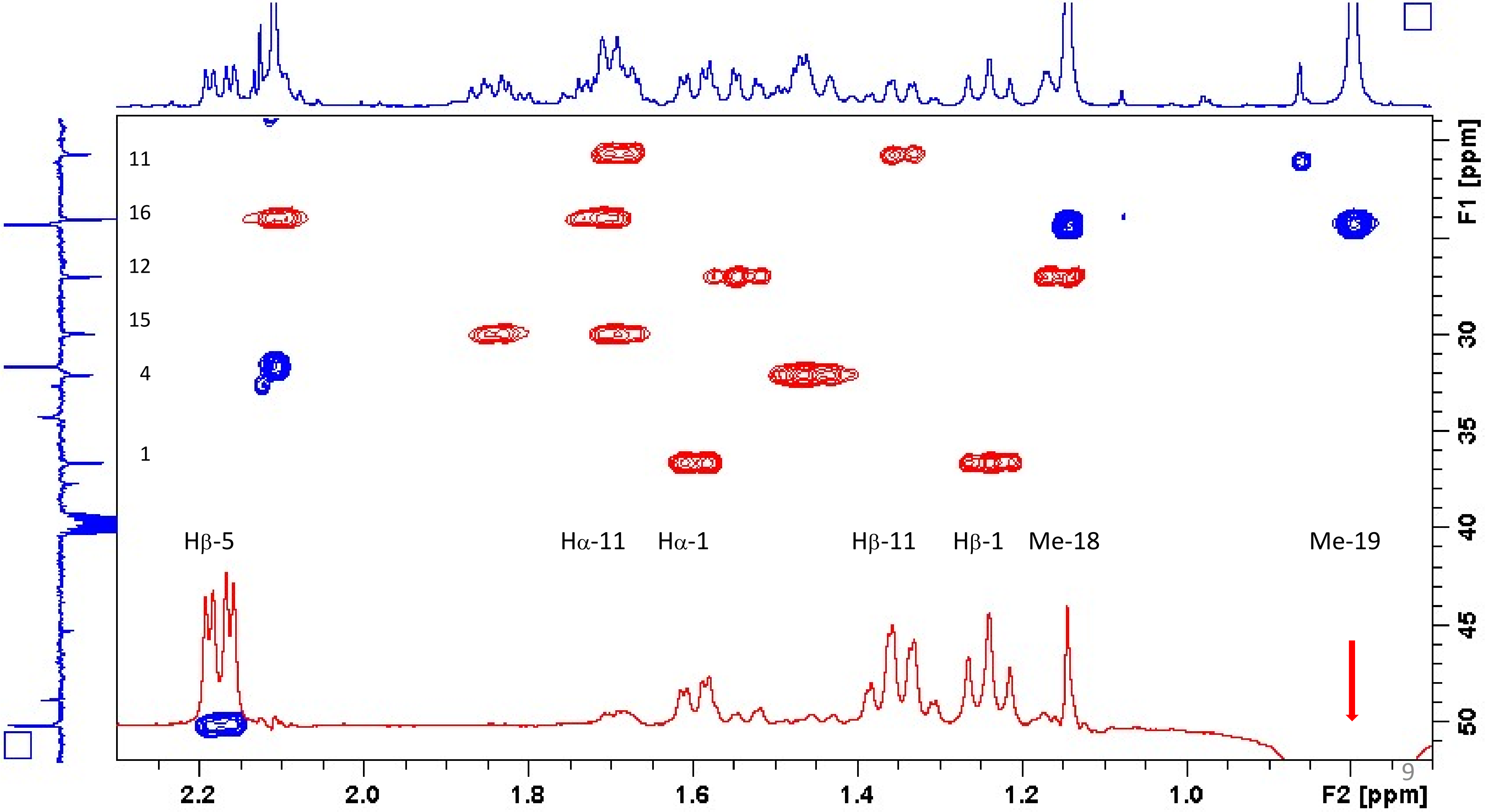

S9 Compound **1**

edHSQC + selROE on Me-18

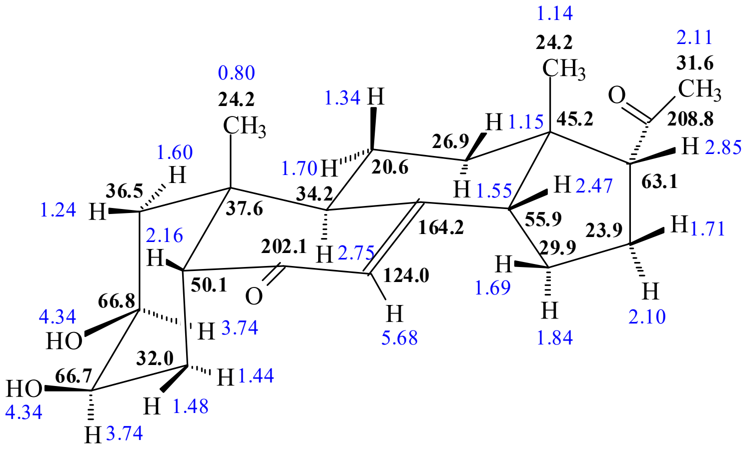

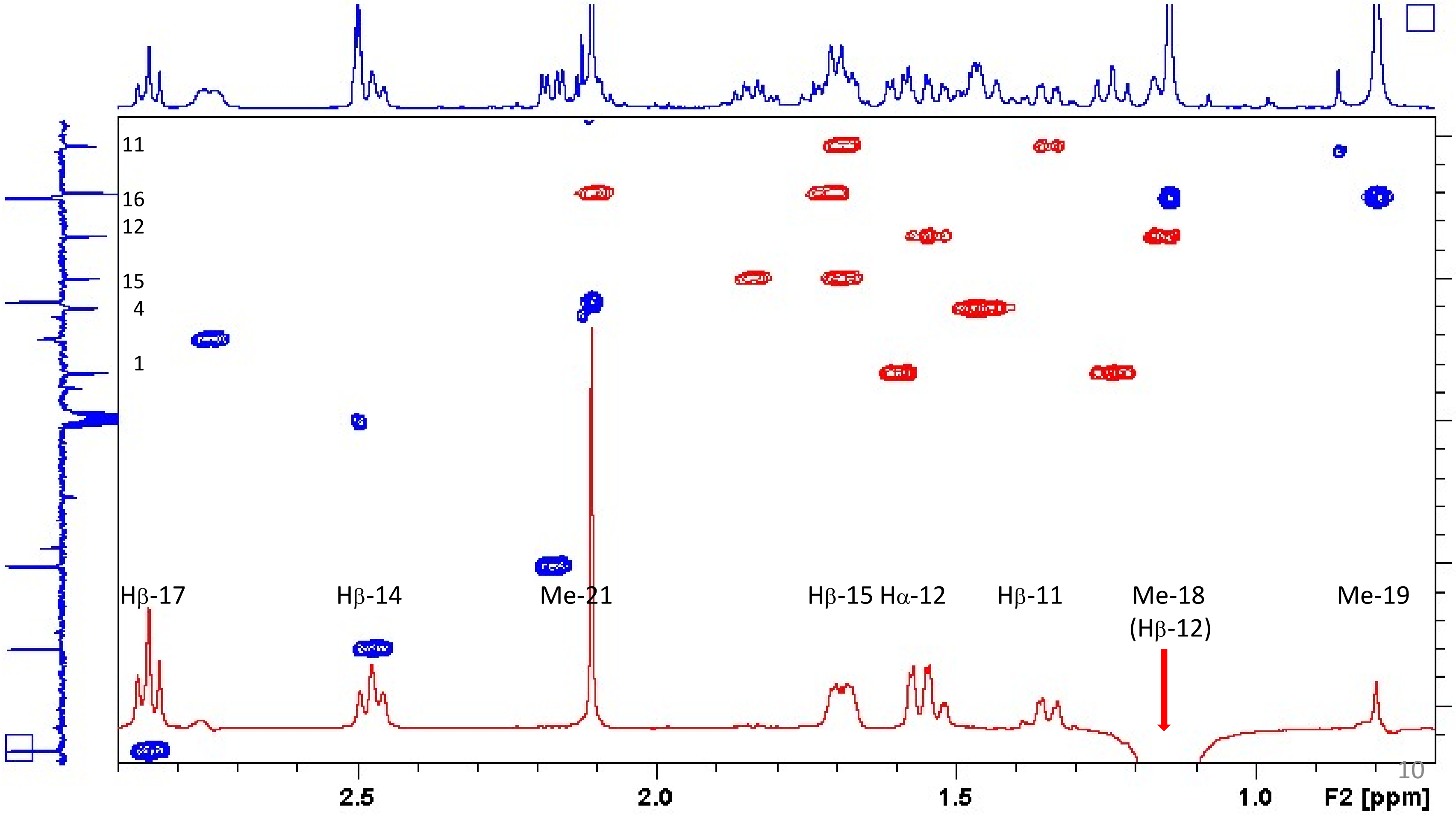

S10 Compound **1**

HMBC

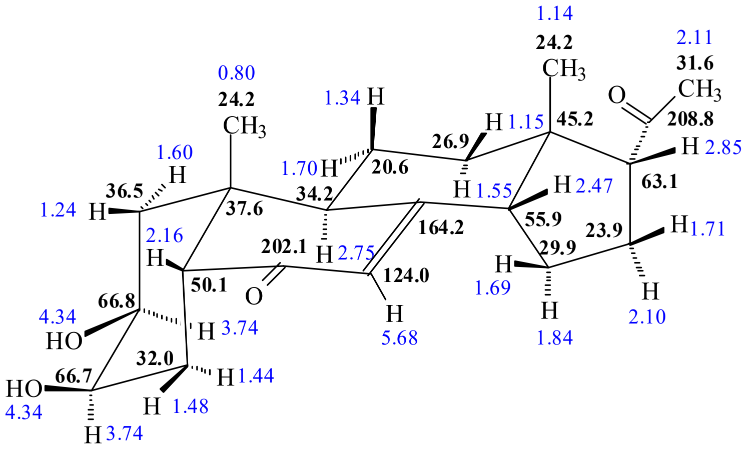

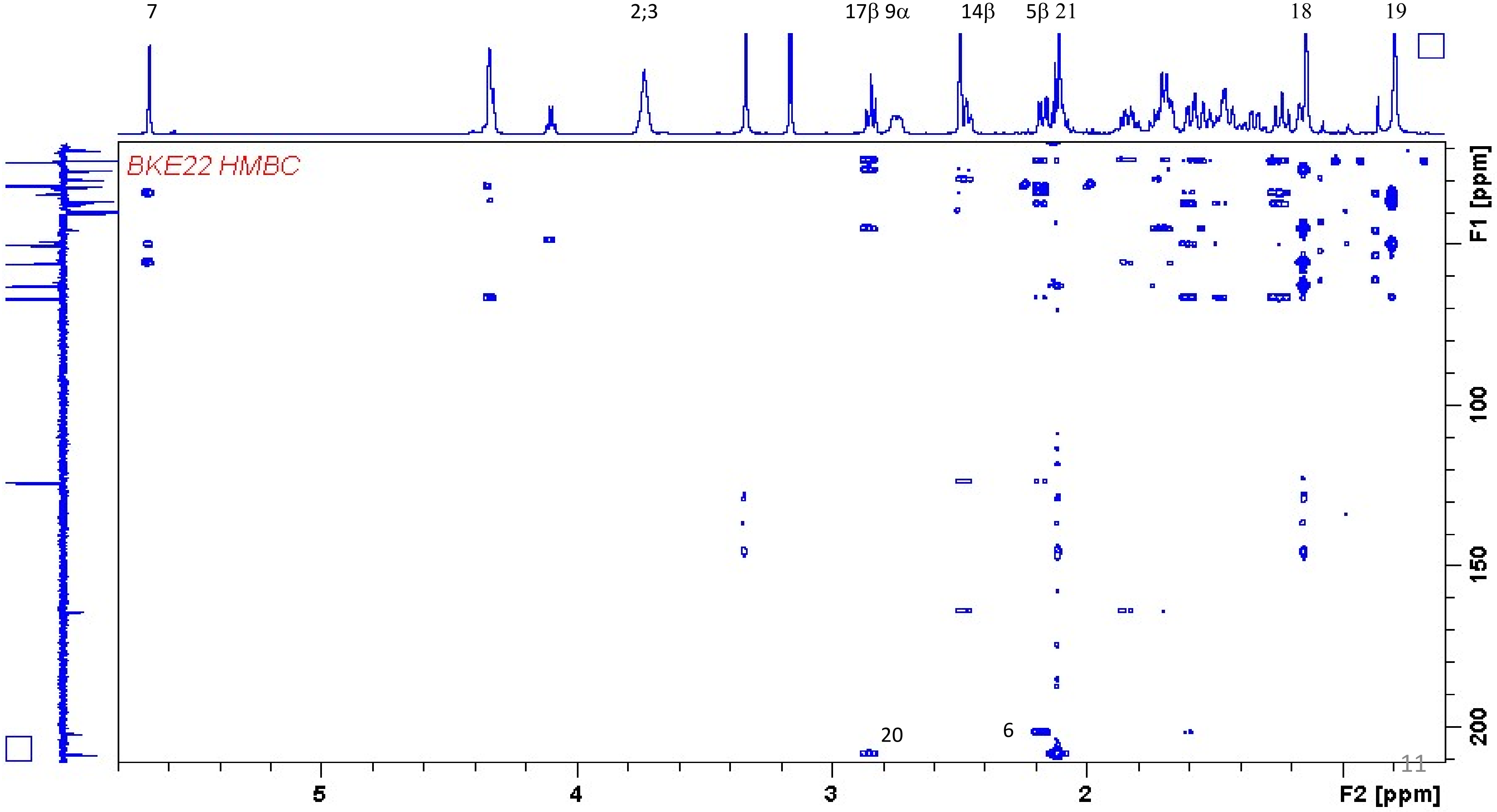

S11 Compound **1**

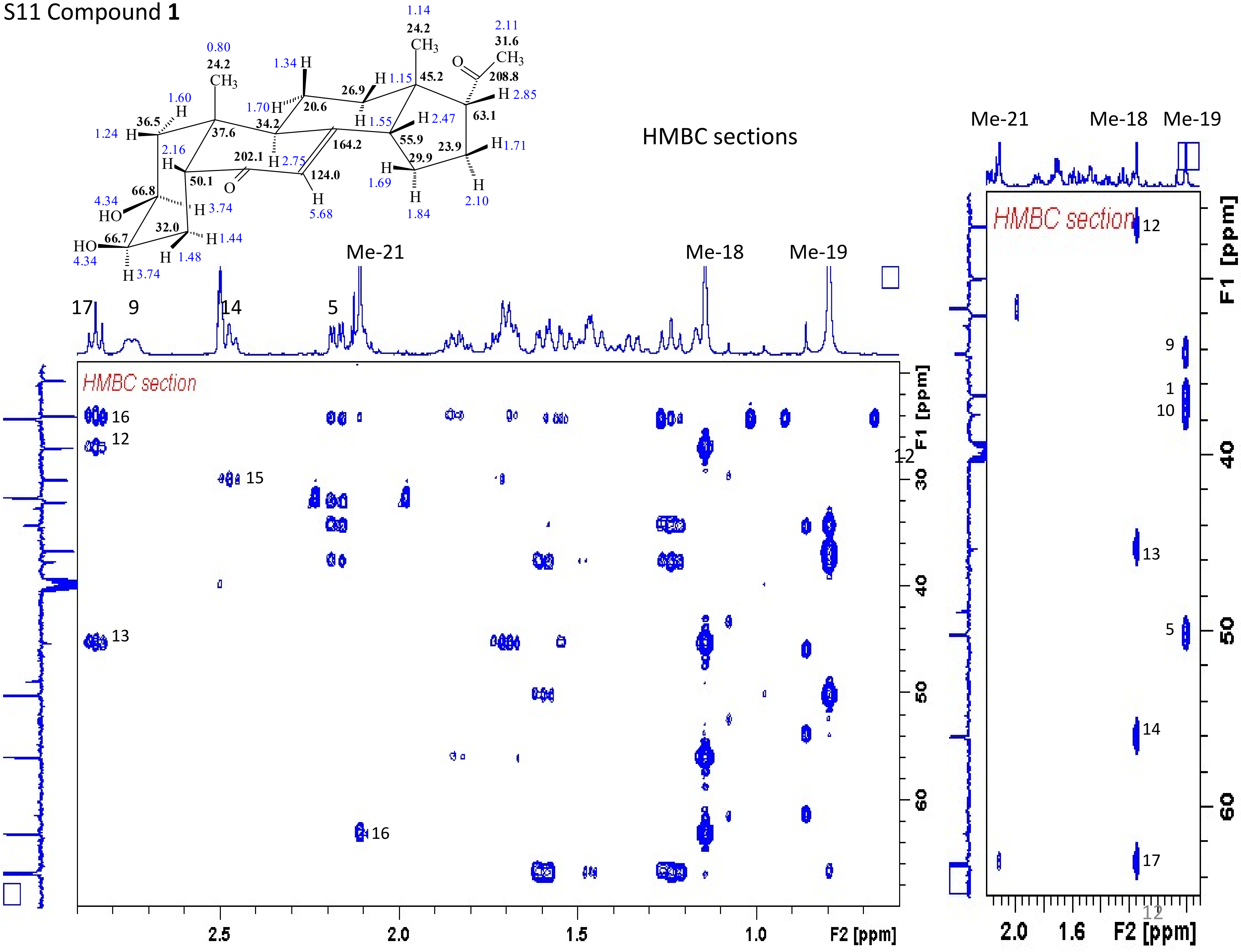

S12 Compound **2**

^{1}H 500 MHz and H-20 signal with decoupling of CH_3-21

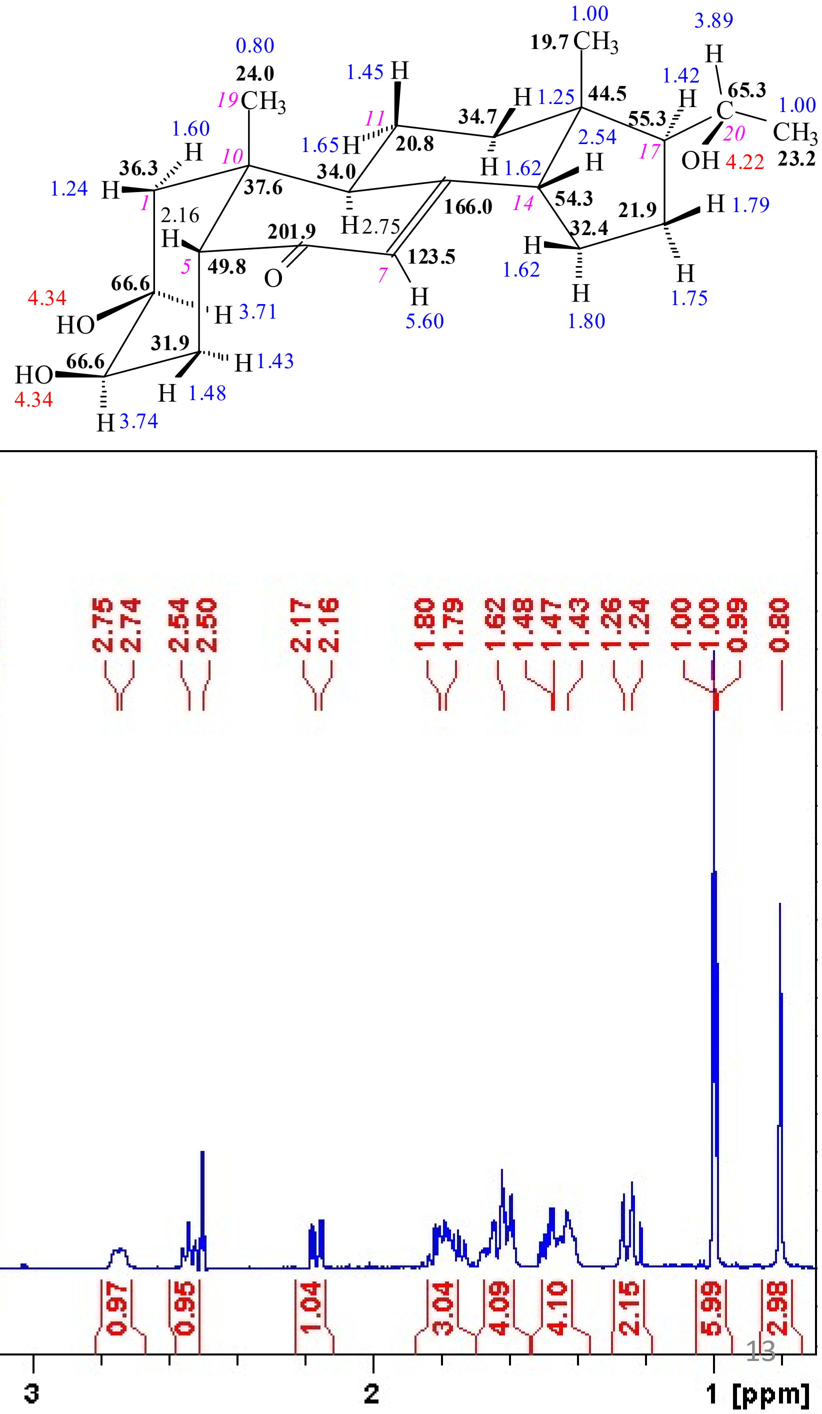

S13 Compound **2**

Identification of spin-systems by selTOCSY on H-5, H-7 and Hβ-14

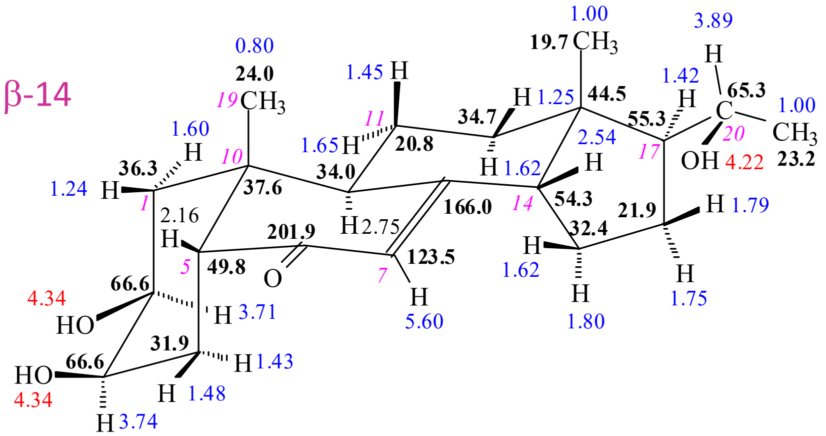

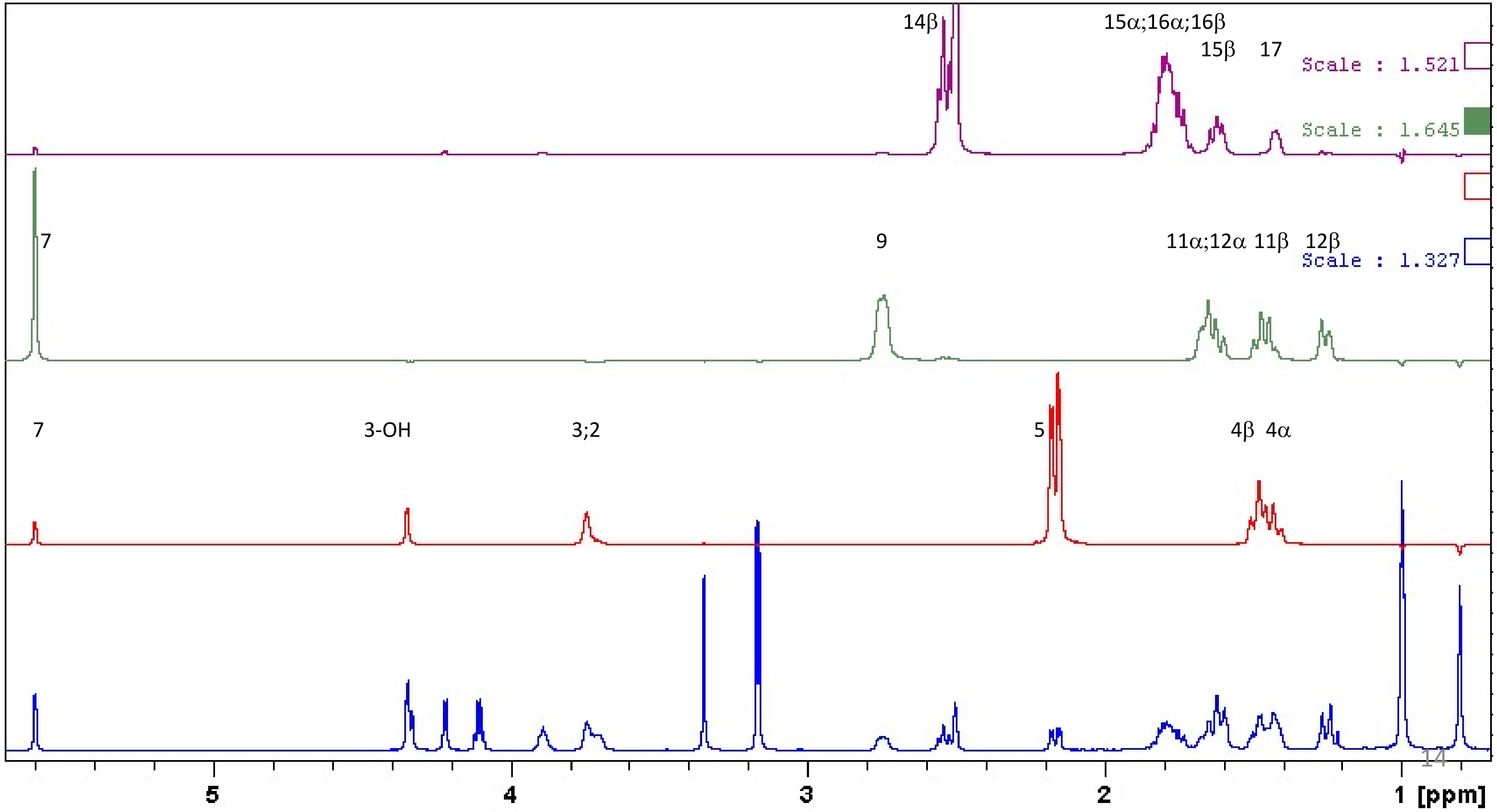

S14 Compound **2**

Steric proximities detected by selROE on H-20, Hβ-14, 20-OH and H-9

Supporting of S configuration at C-20

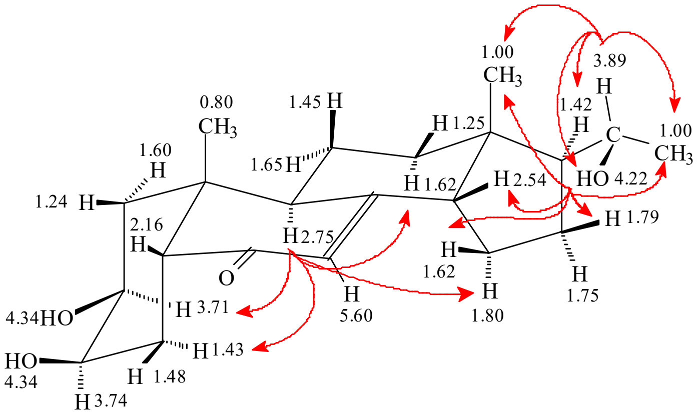

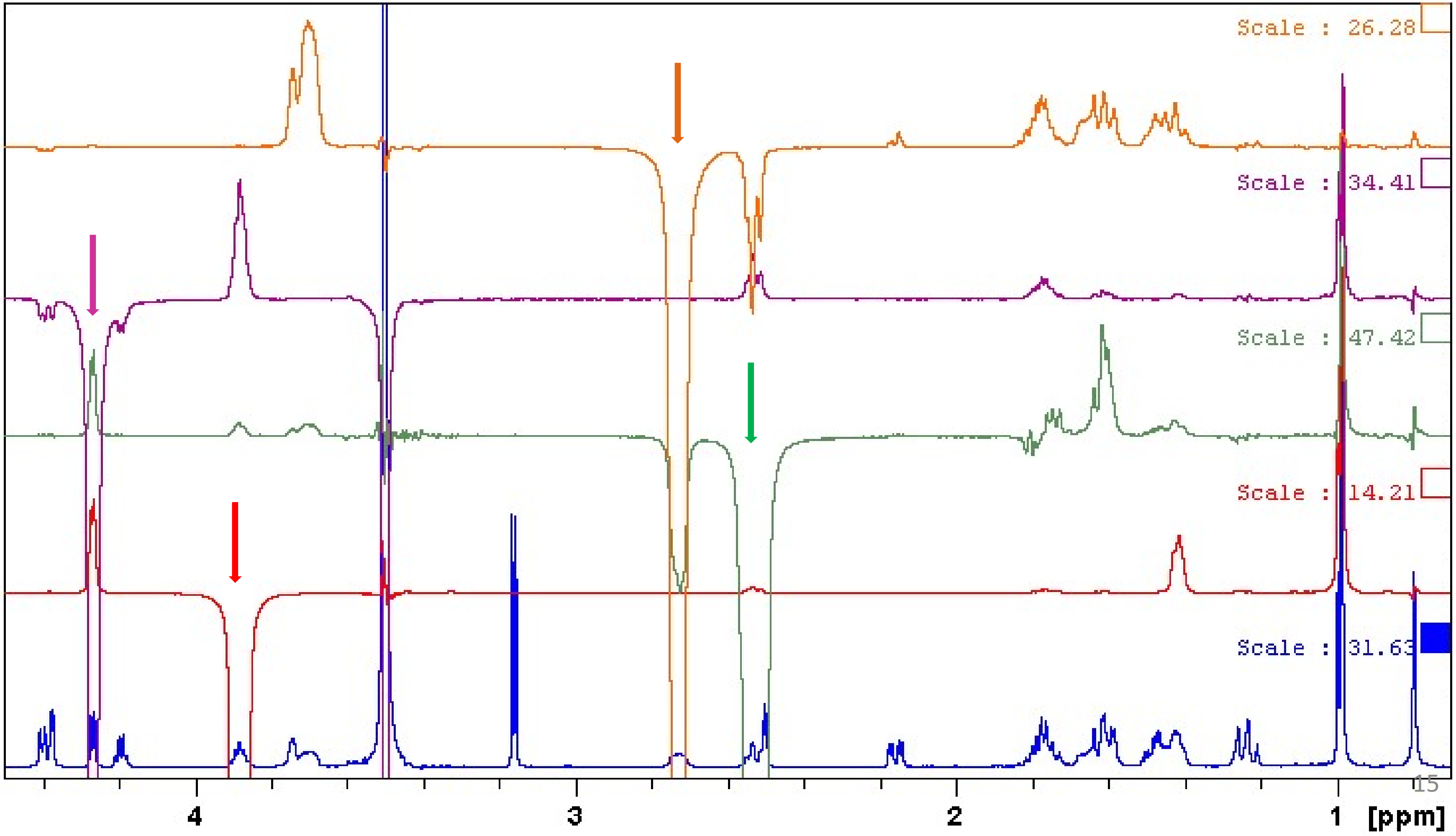

S15 Compound **2**

DeptQ 125 MHz

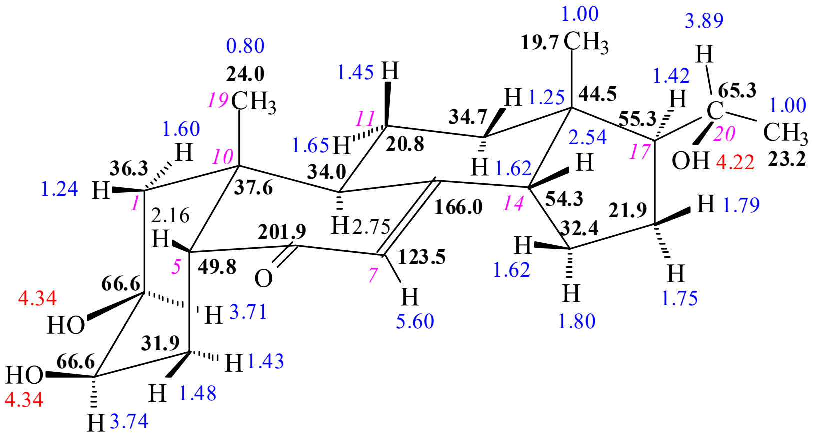

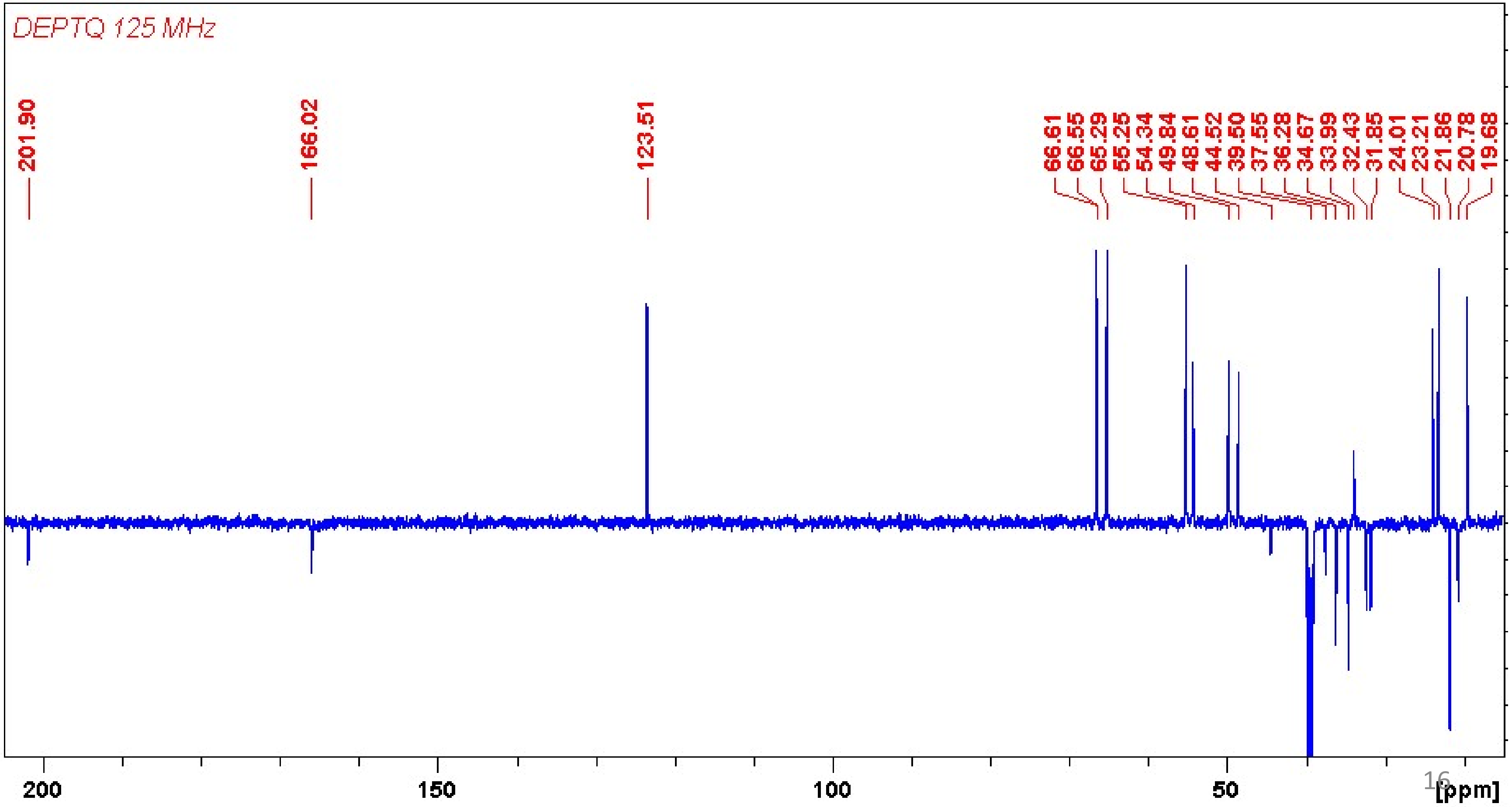

S16 Compound **2**

edHSQC + selROE on Me-19

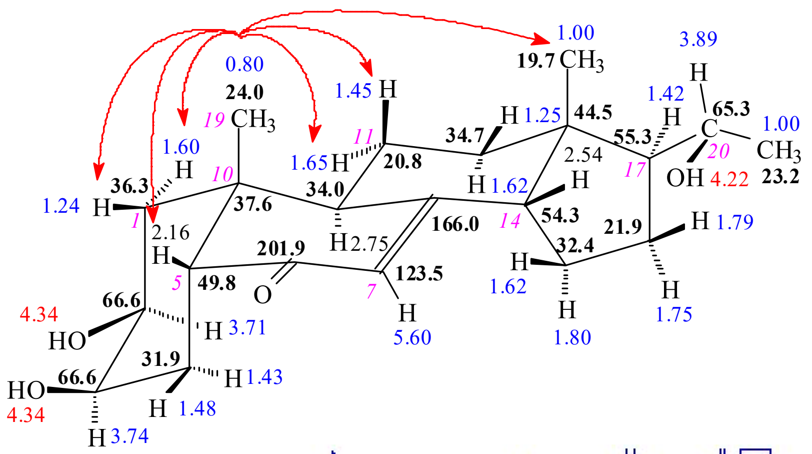

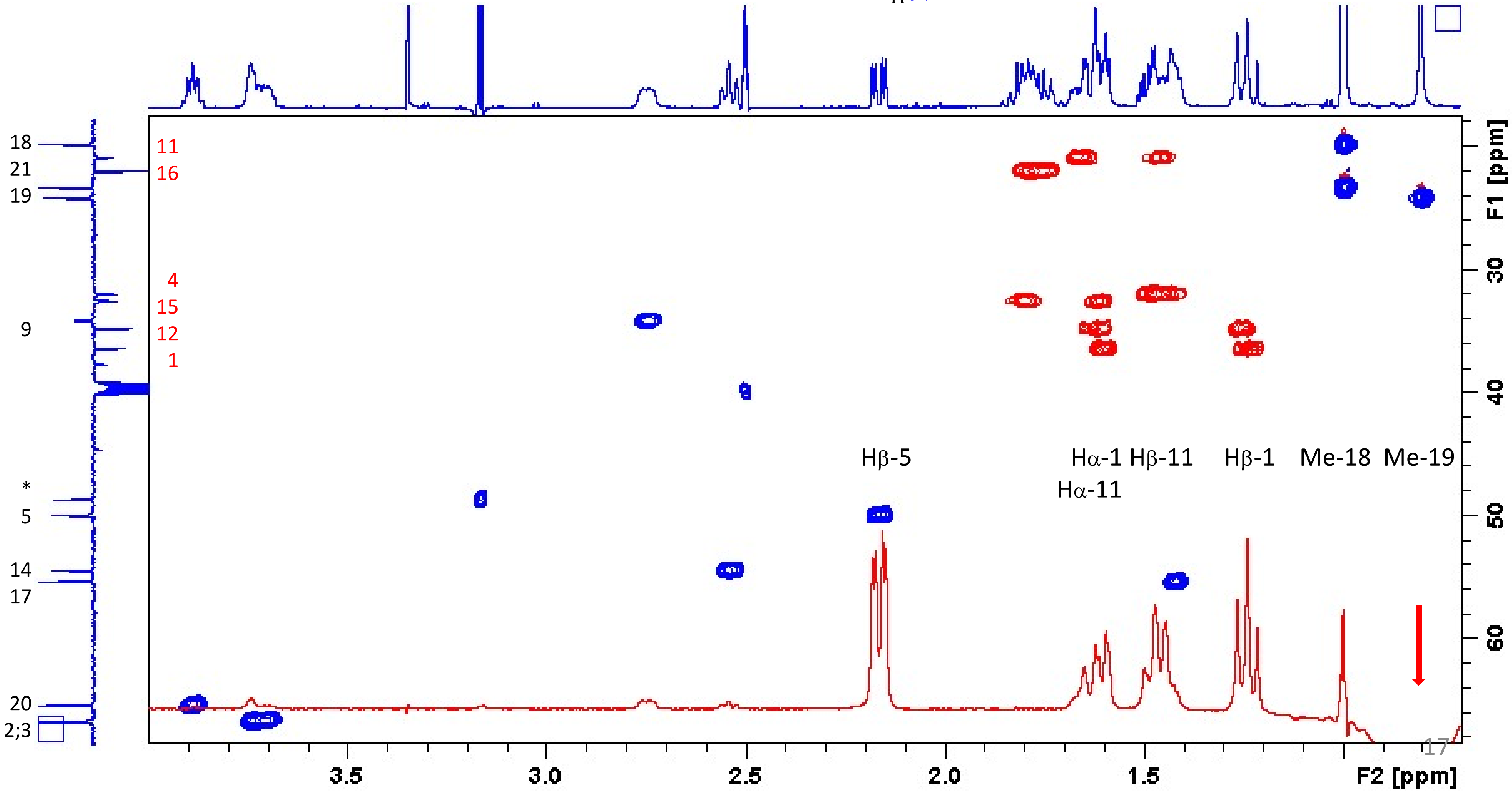

S17 Compound **2**

HMBC

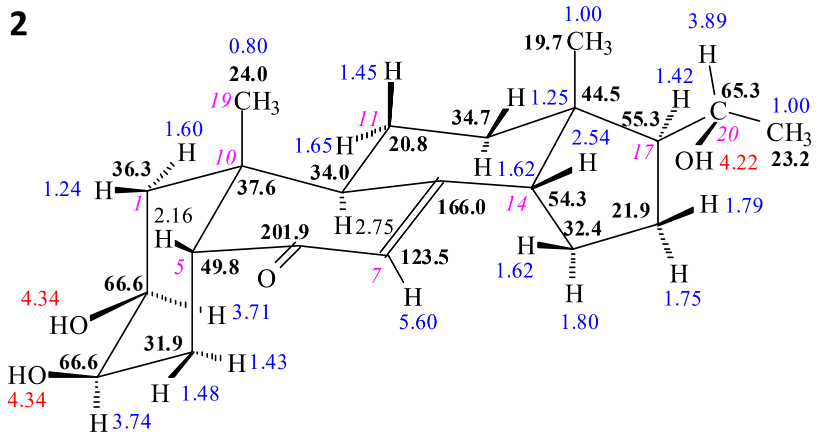

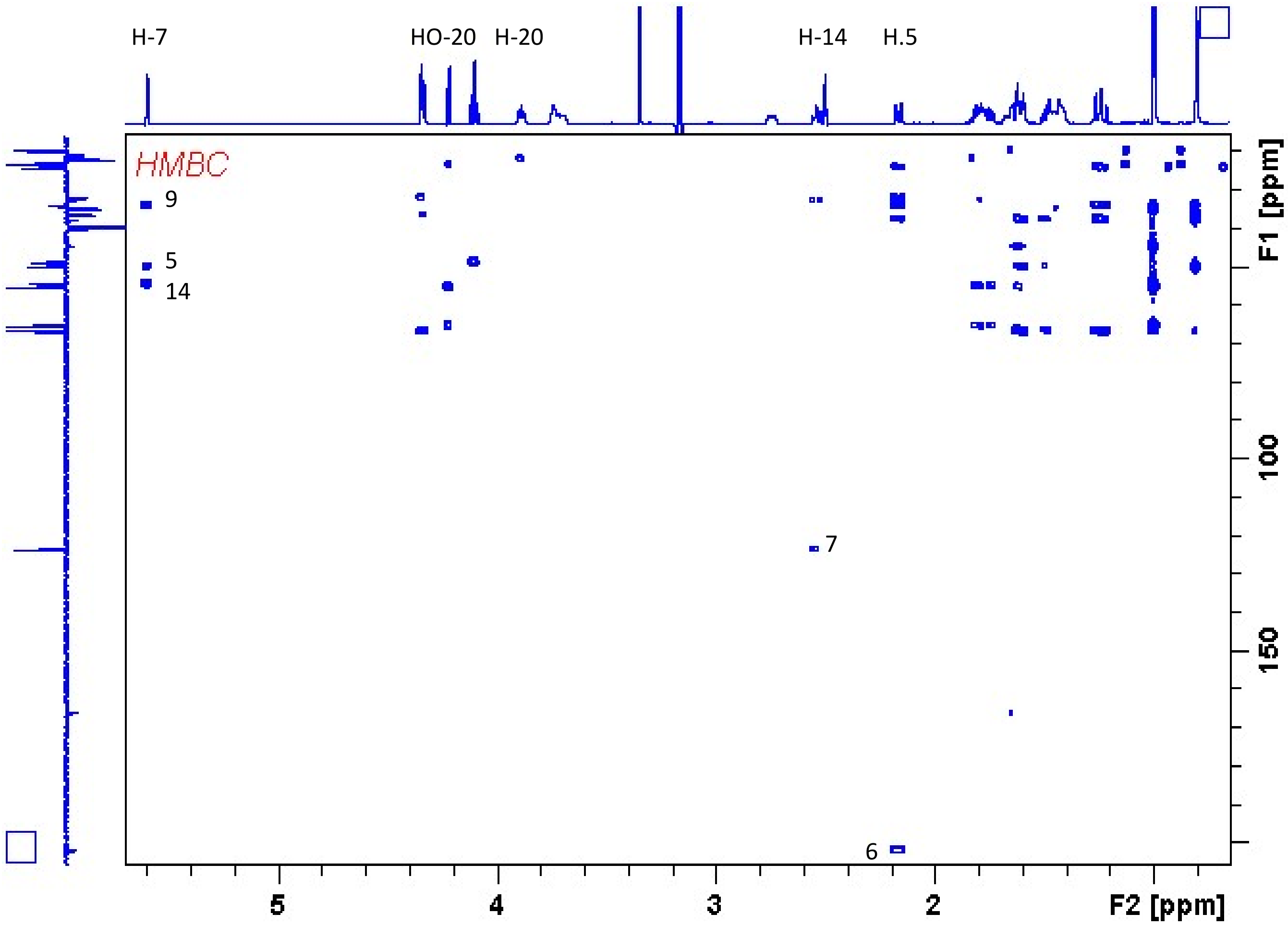

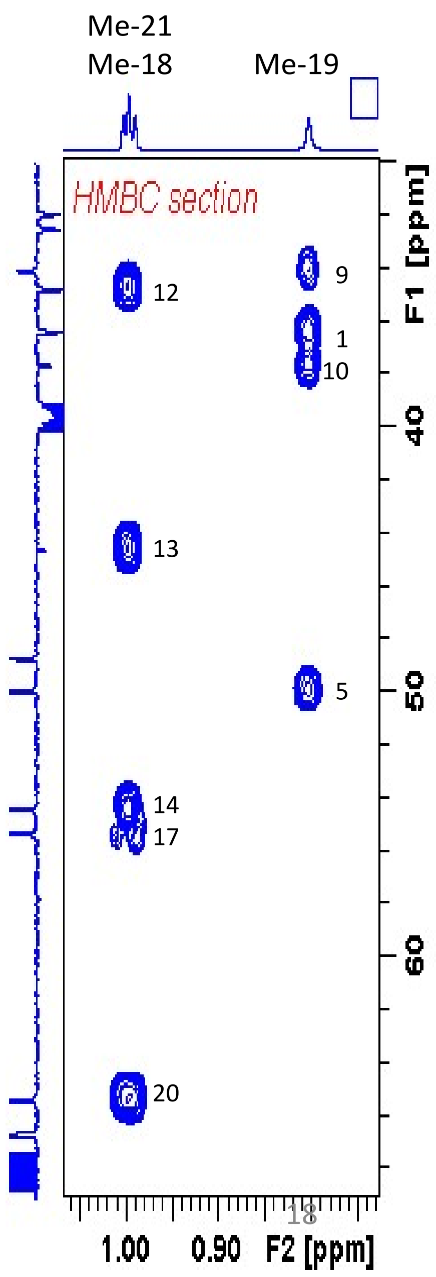

S18 Compound **3**

^{1}H 600 MHz

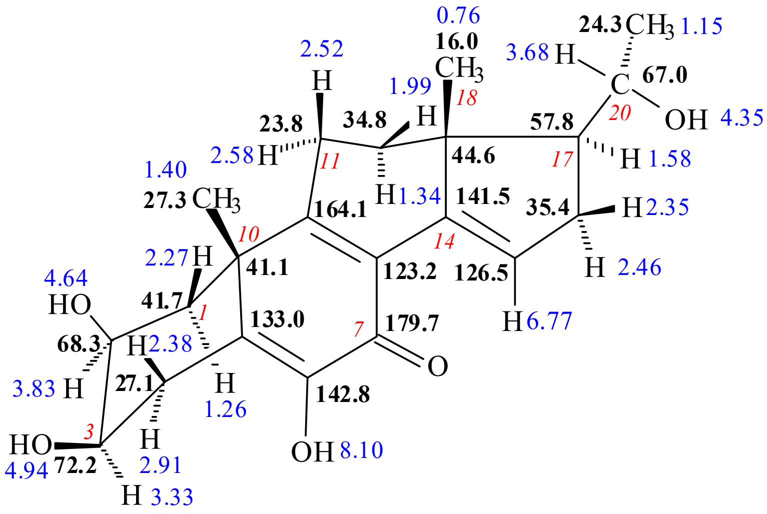

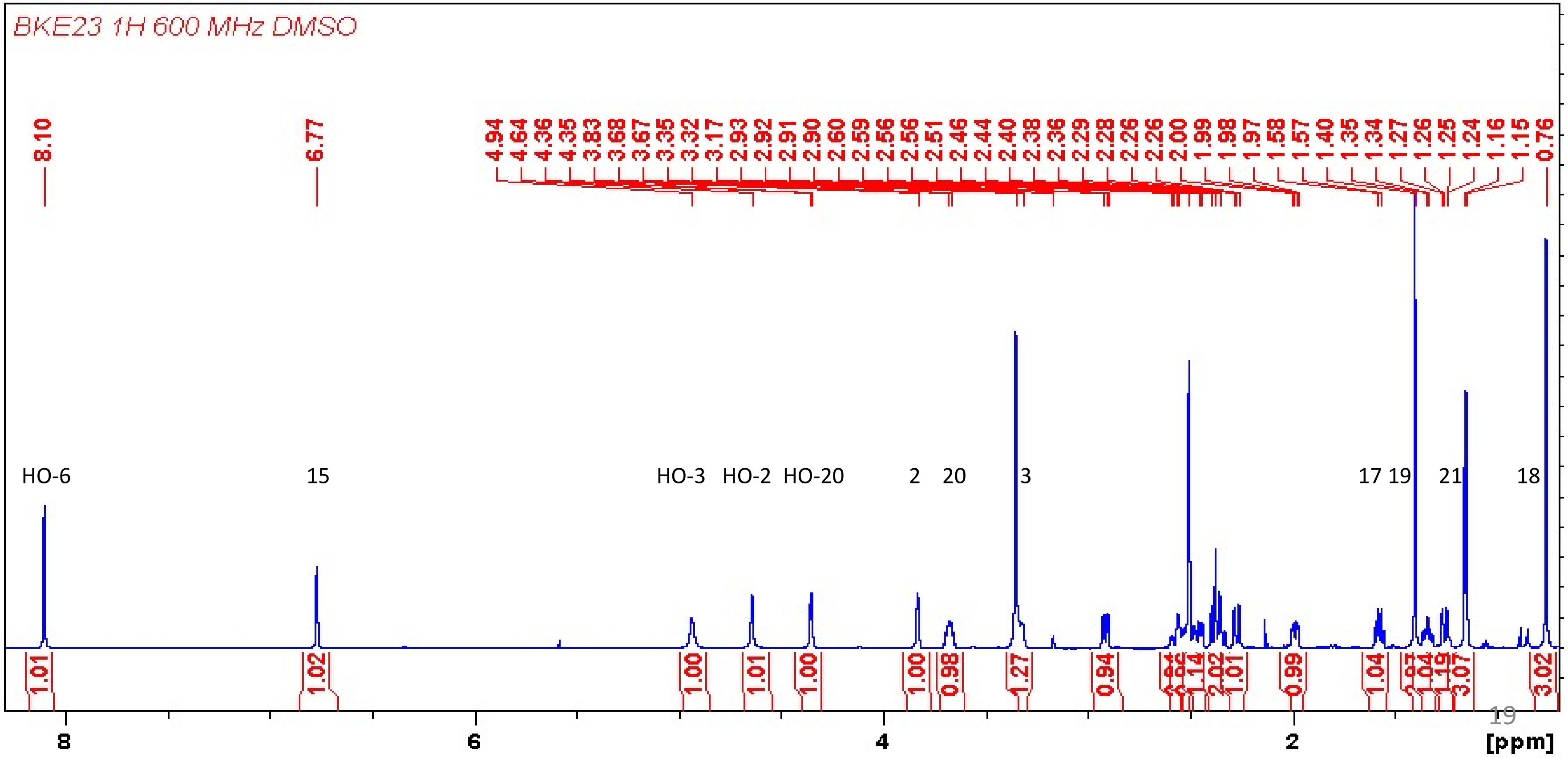

S19 Compound **3**

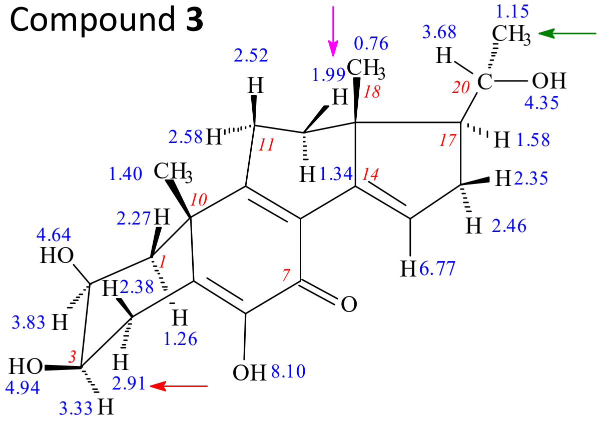

Identification of the spin-systems by selTOCSY on signals:
H-21, Hα-4 and Hβ-12

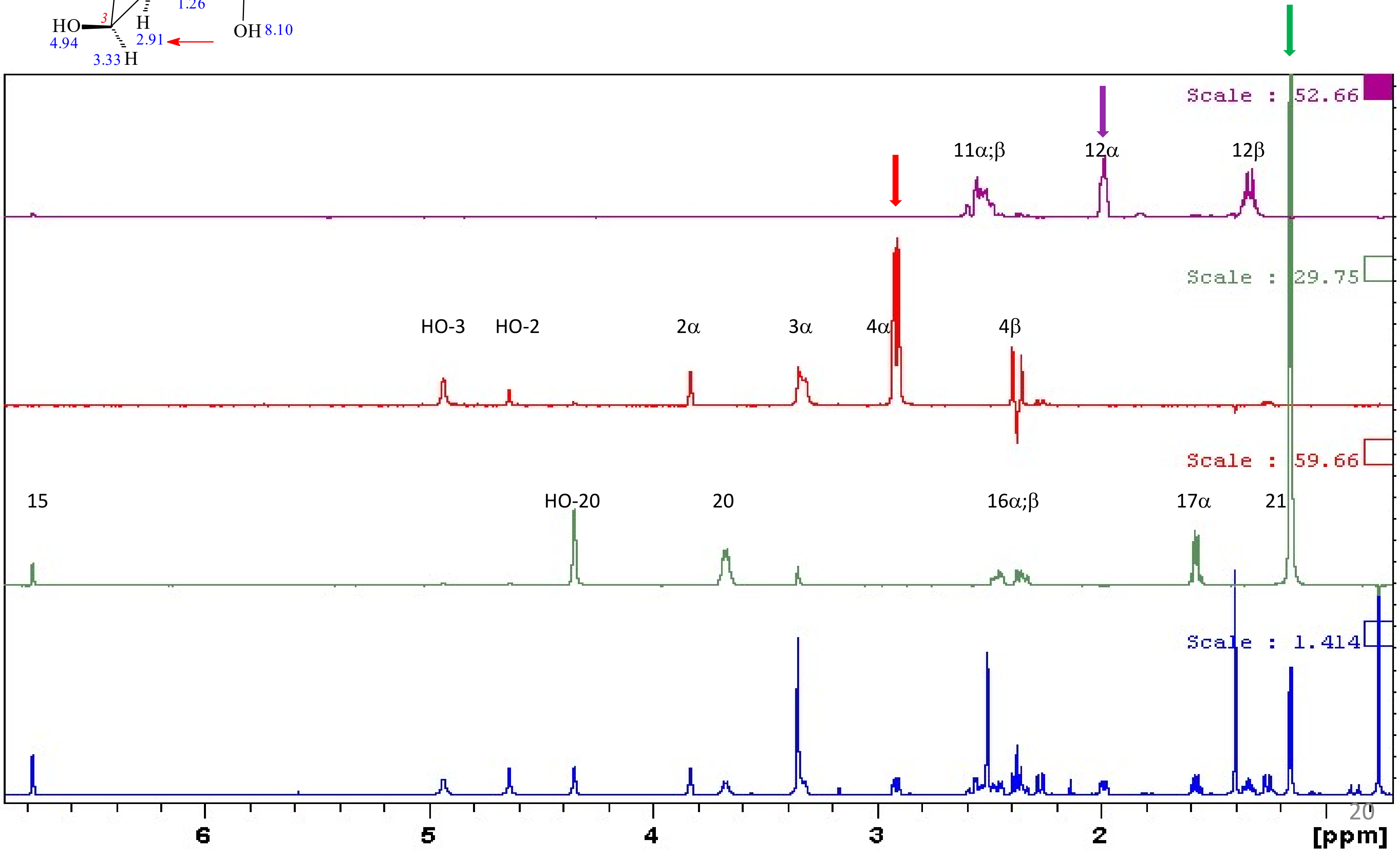

S20 Compound **3**

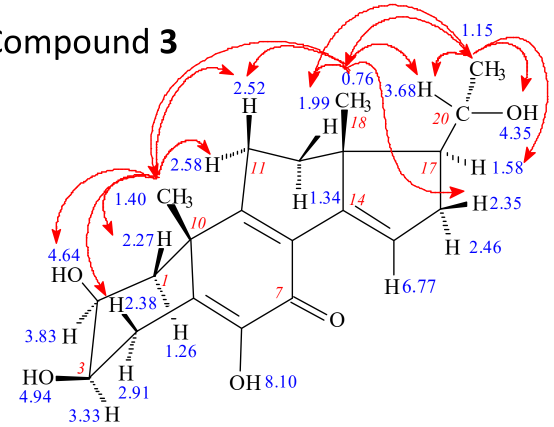

Steric proximities detected by selROE on signals: H-18, H-19 and H-21.

Supporting of *S* configuration at C-20

J(H-17,H-20) ~ 10 Hz

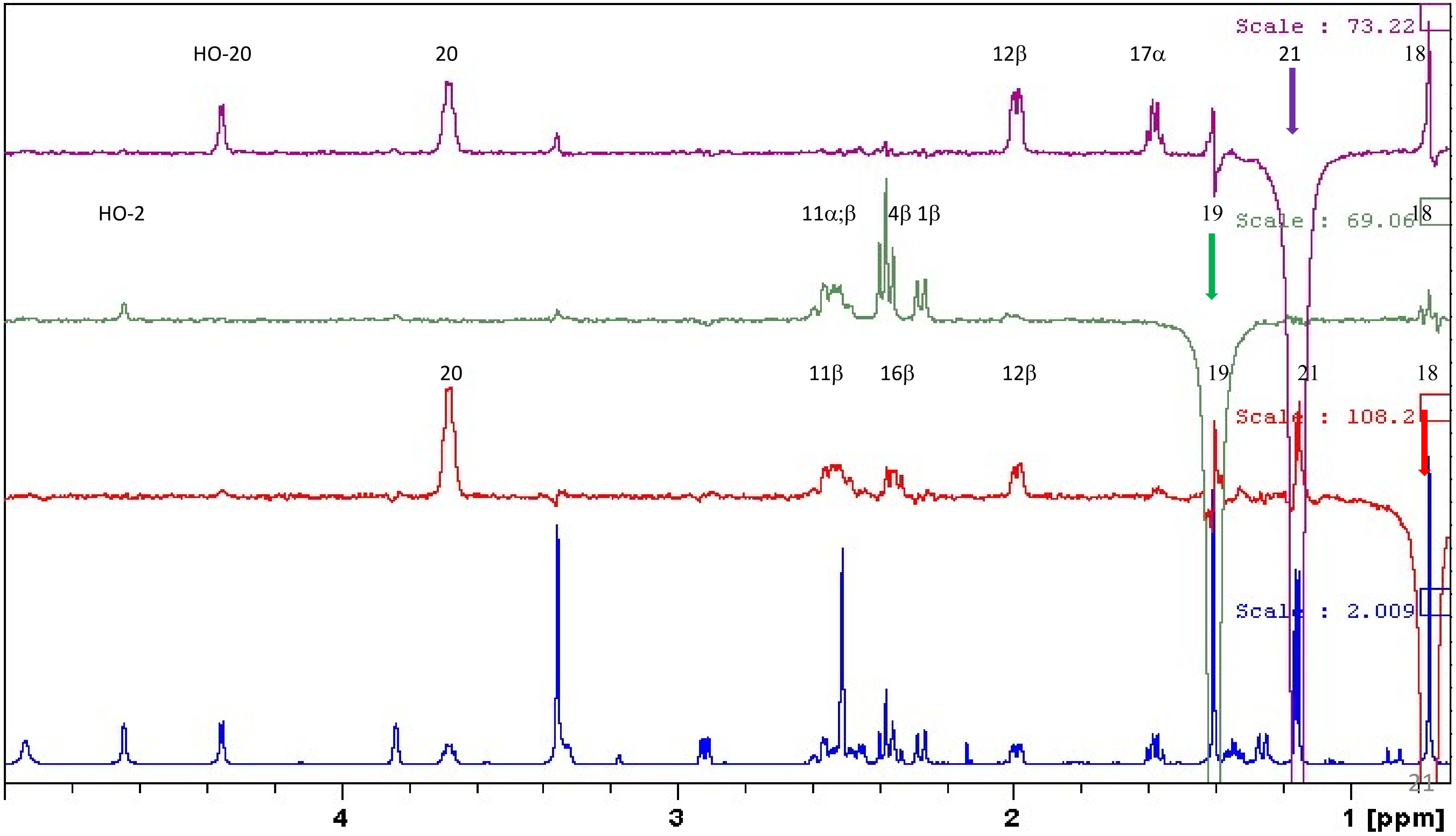

S21 Compound **3**

DEPTQ 150 MHz

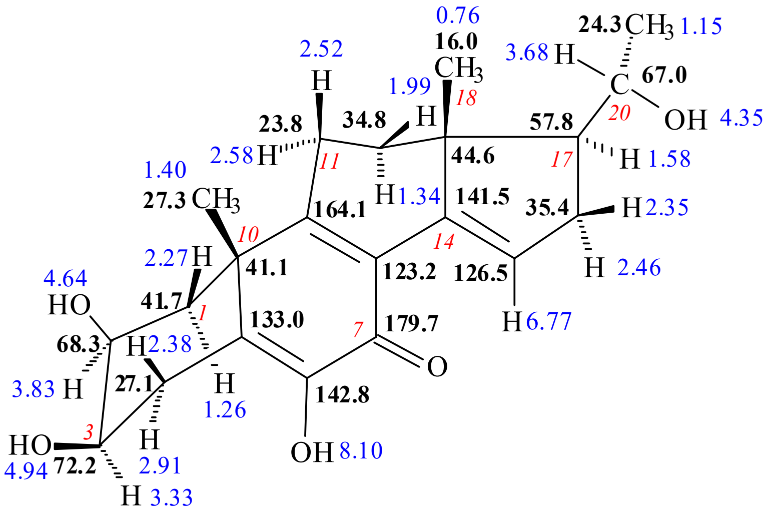

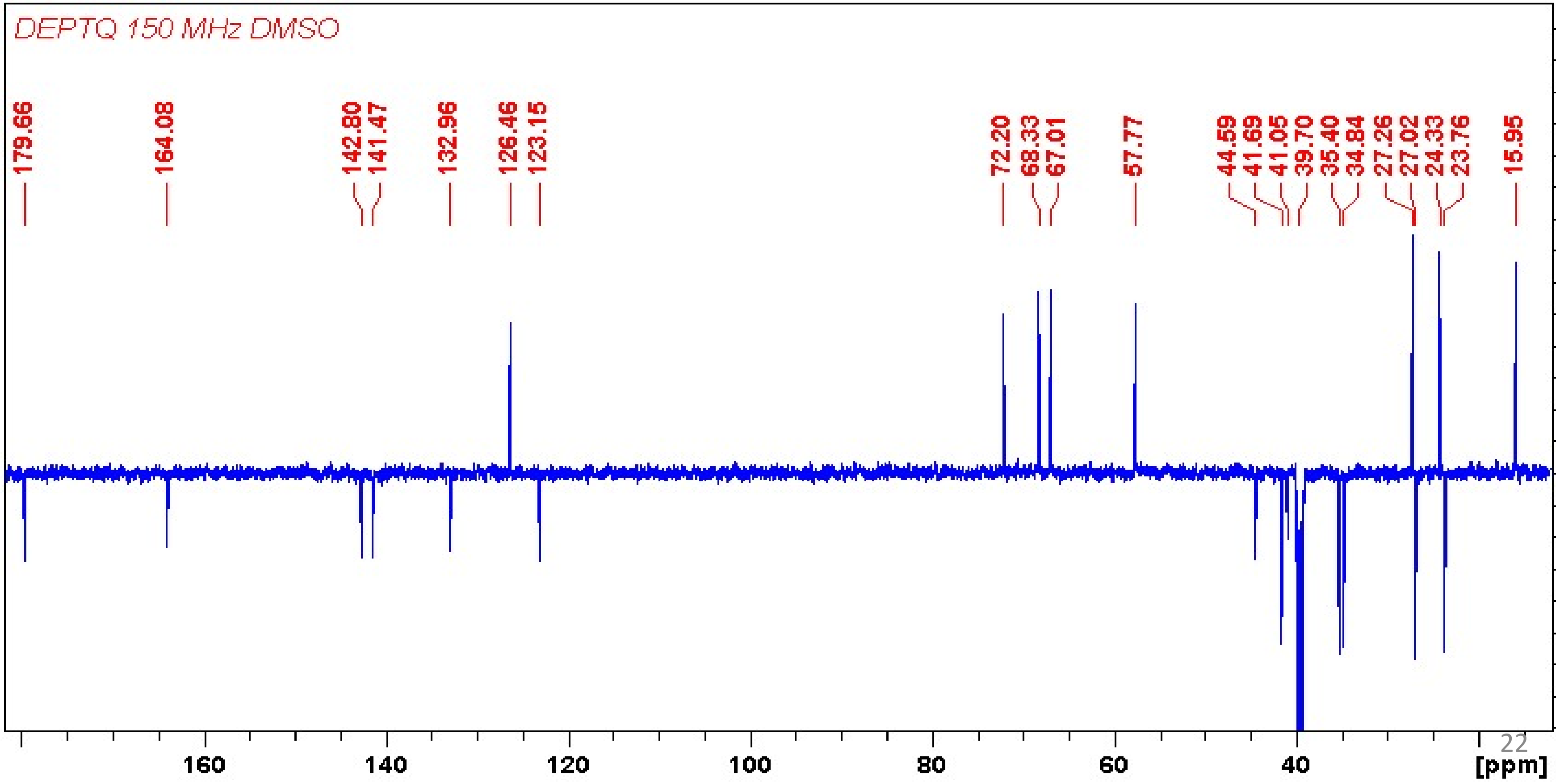

S22 Compound **3**

edHSQC

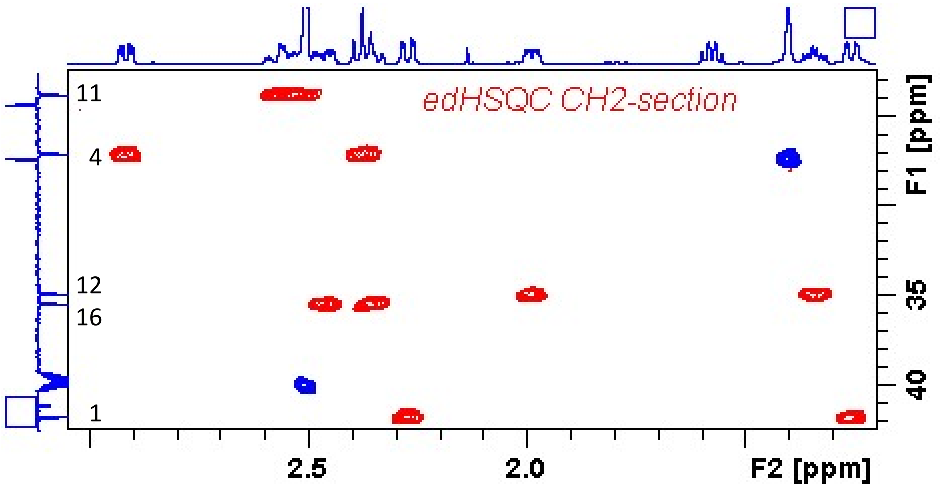

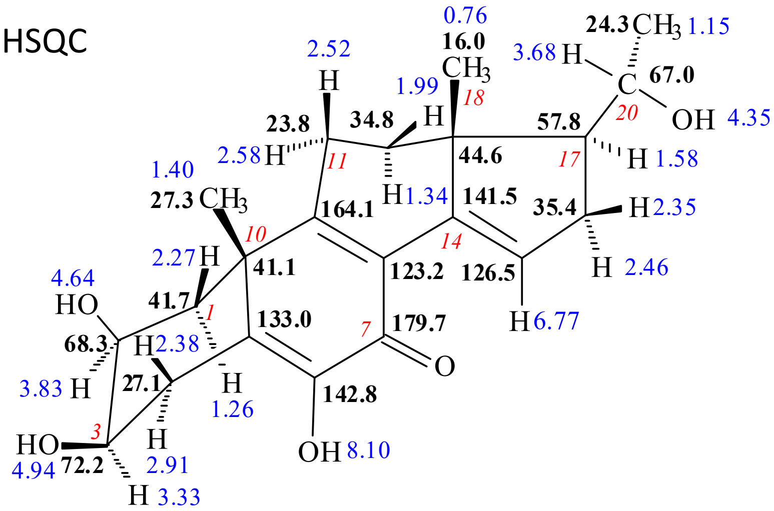

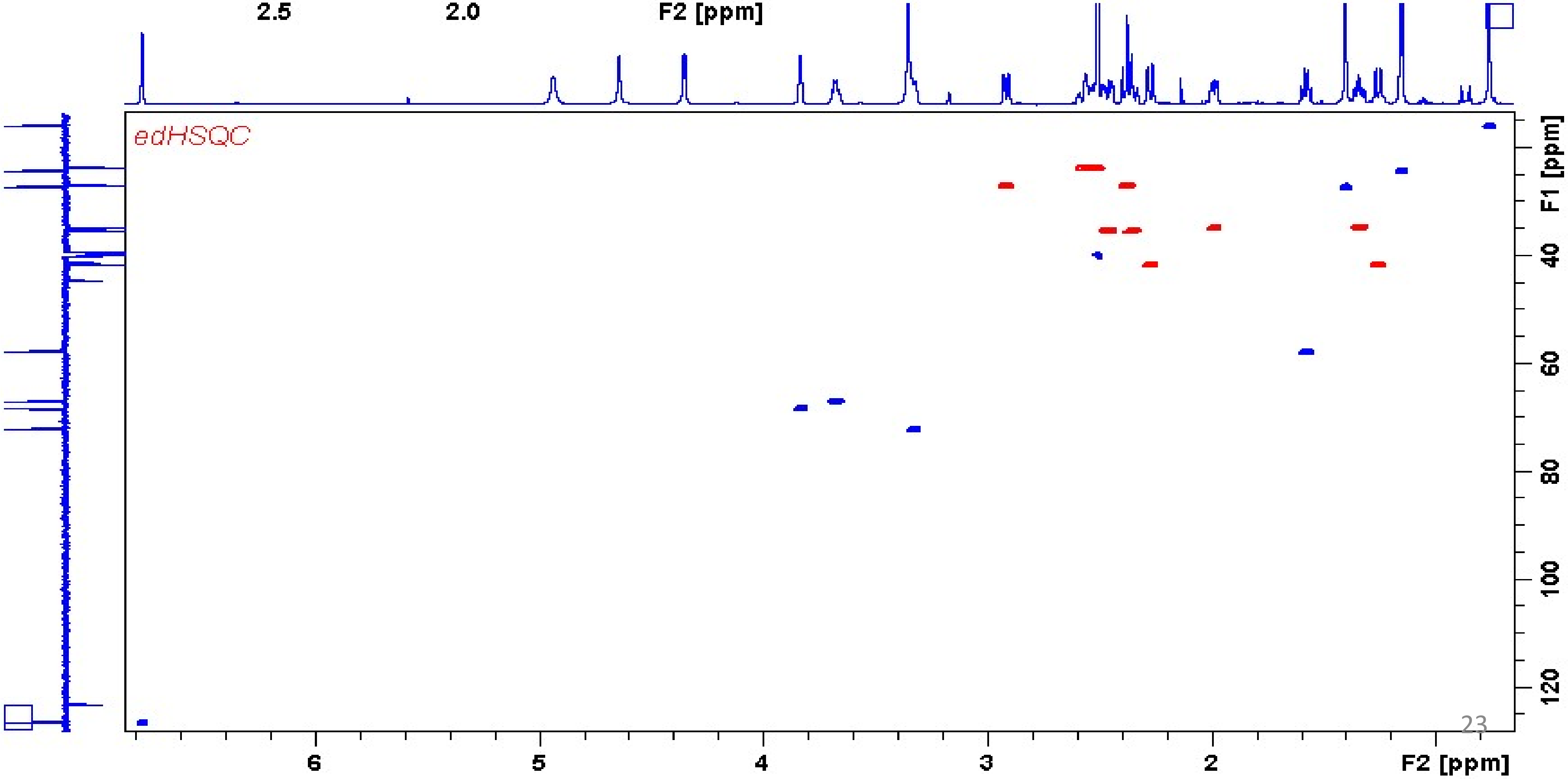

S23 Compound **3**

HMBC

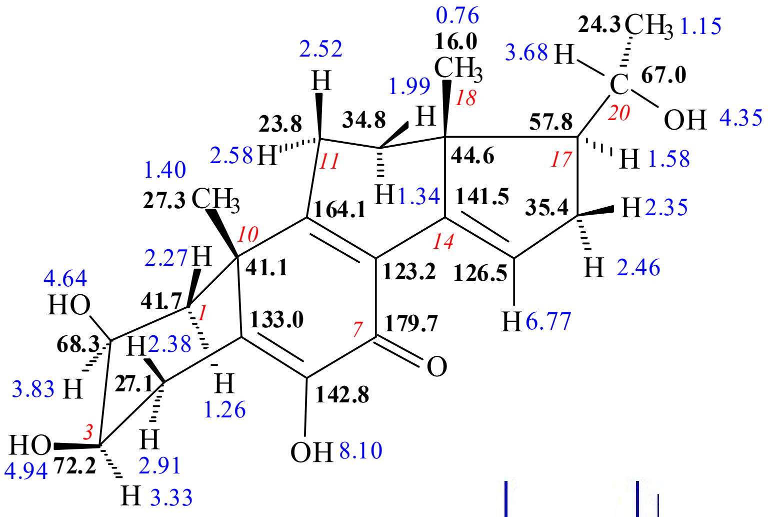

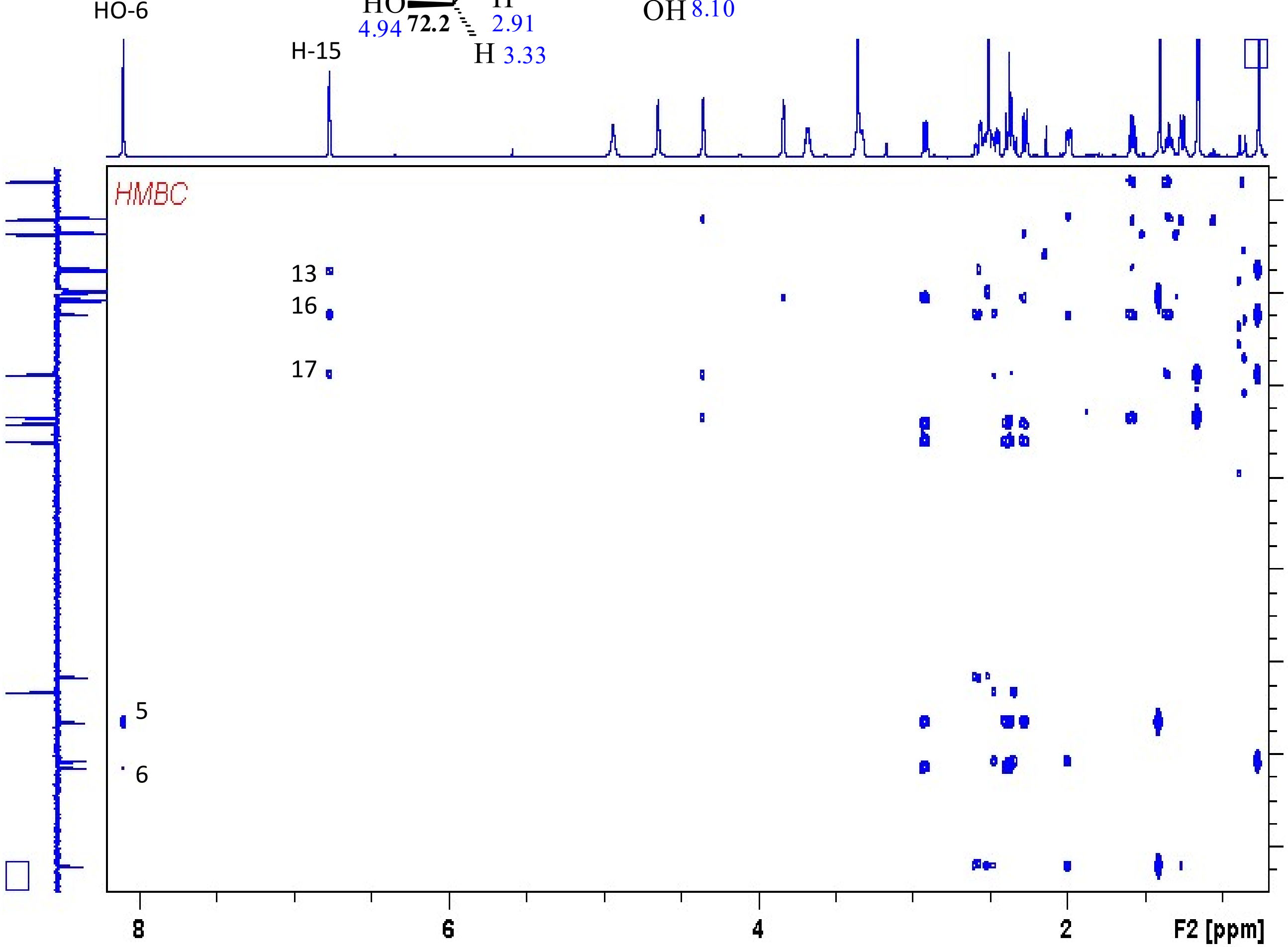

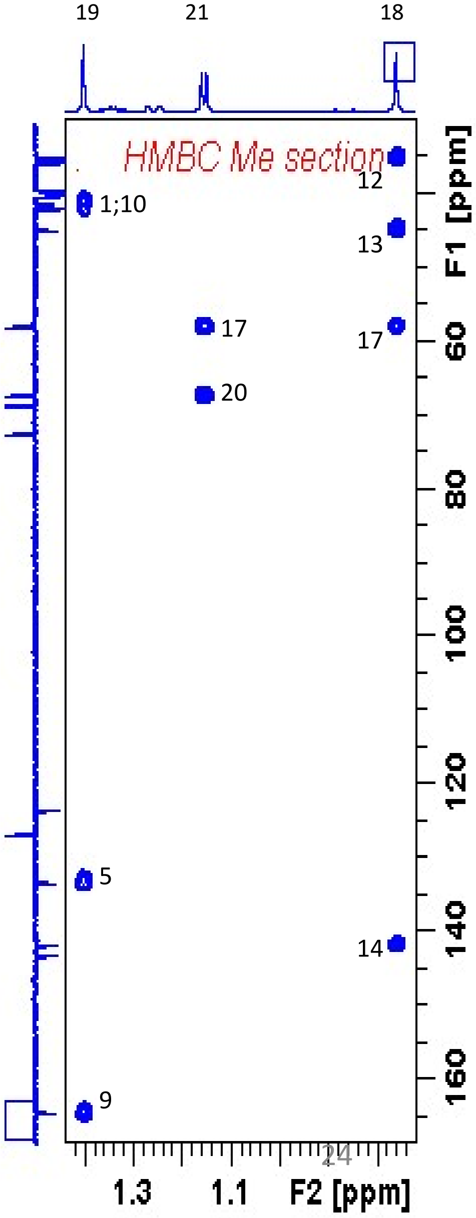

S24 Compound **4**

^{1}H 500 MHz

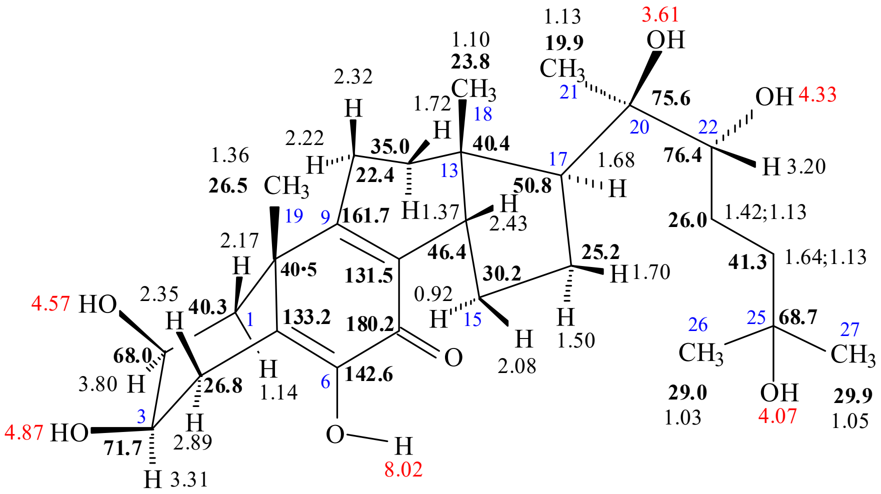

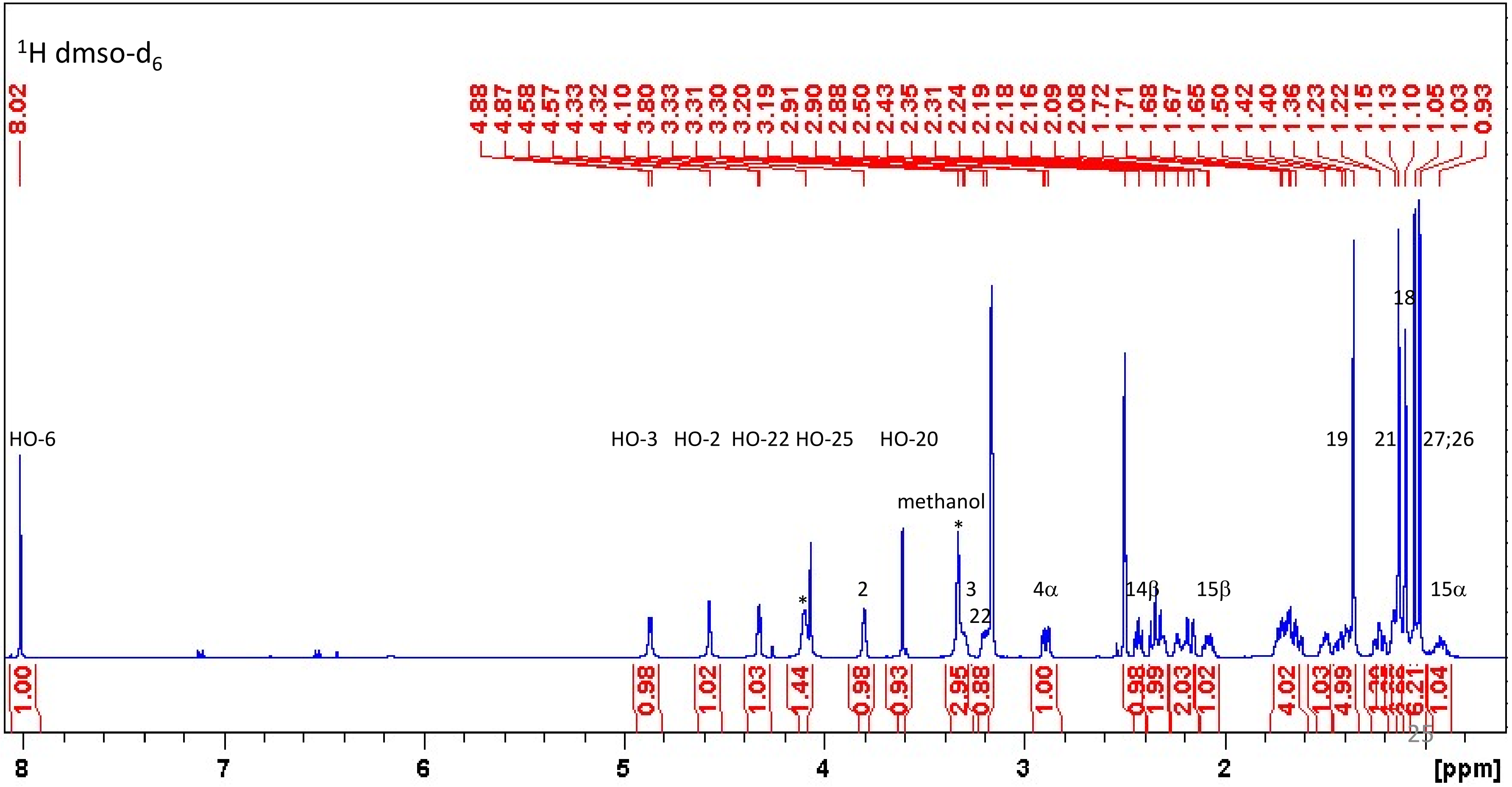

S25 Compound **4**

Steric proximities detected sel-Roesy (τ_{mix}: 300 ms) on Me-18 and Hβ-14

Identification of spin-system by selTOCSY on Hβ-14

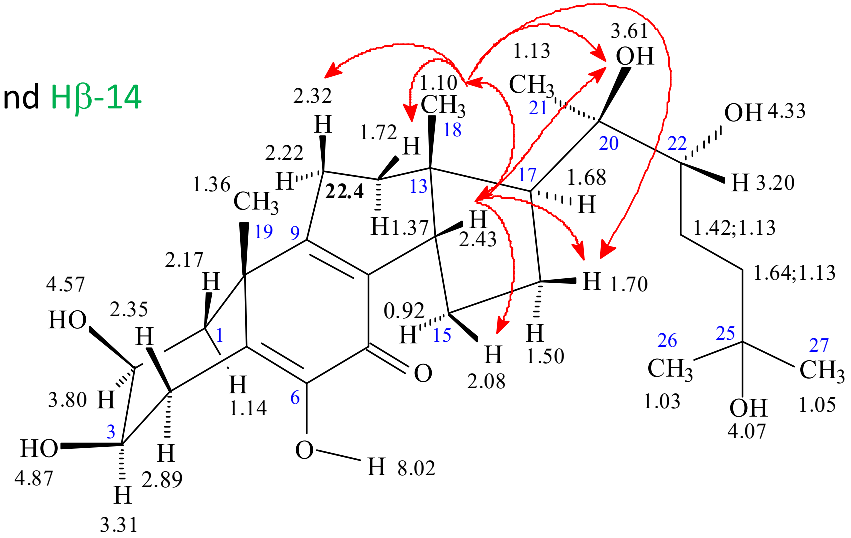

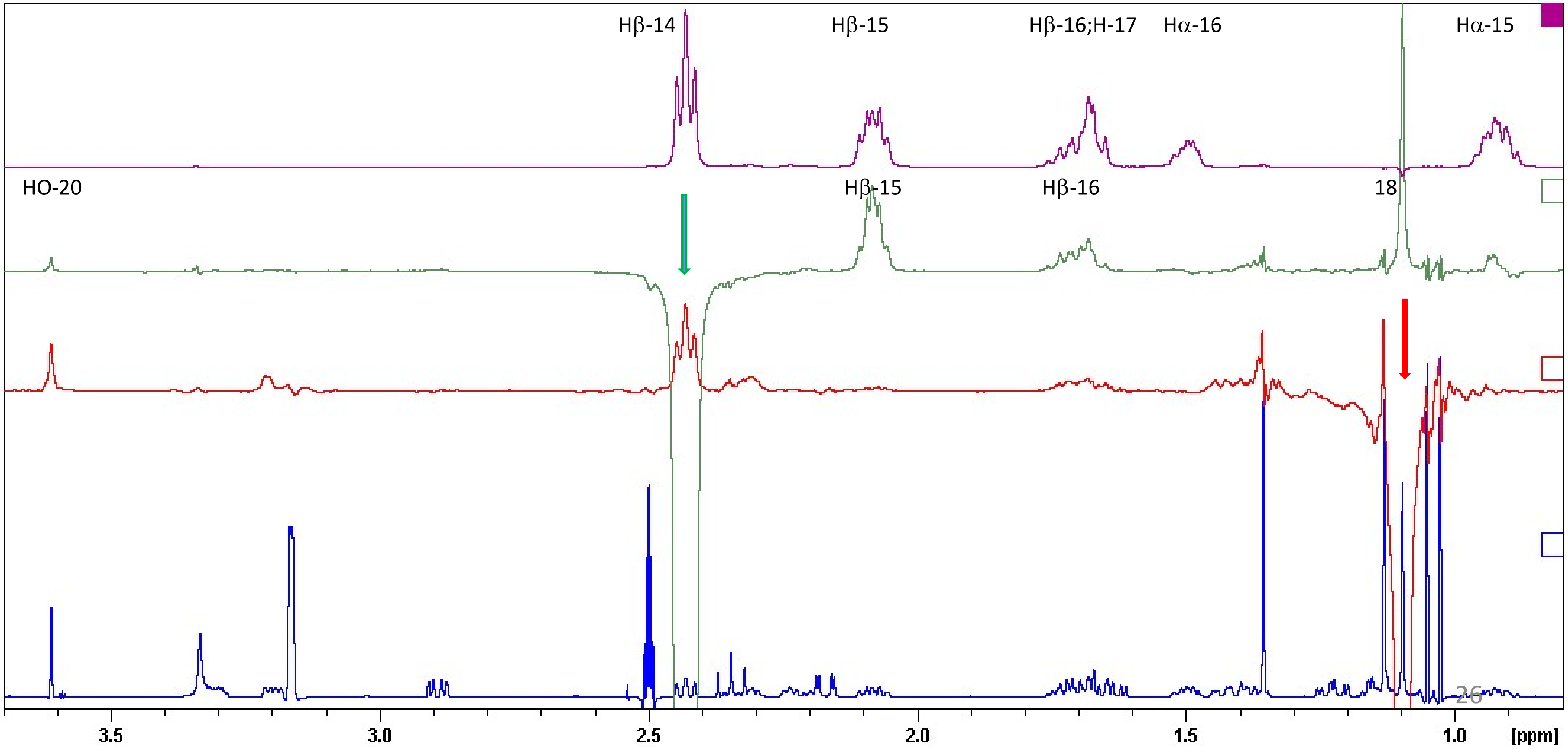

S26 Compound **4**

DeptQ 125 MHz

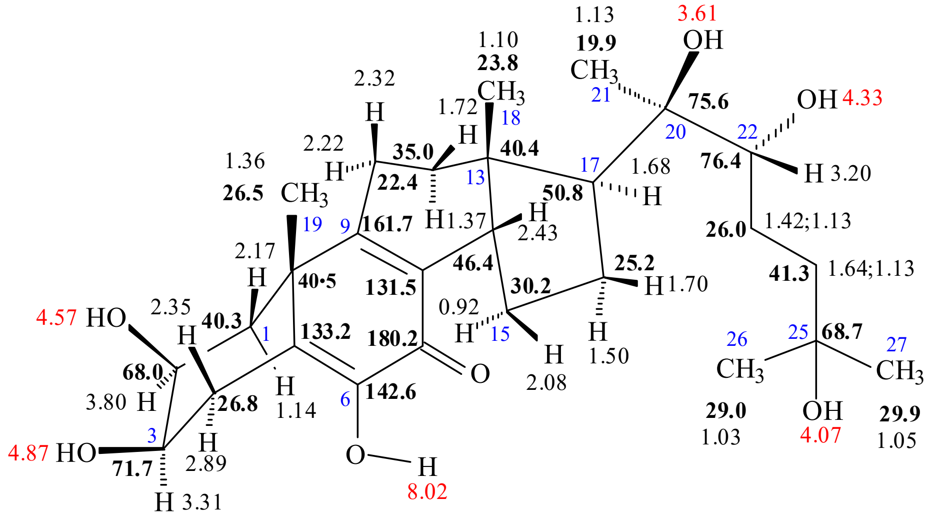

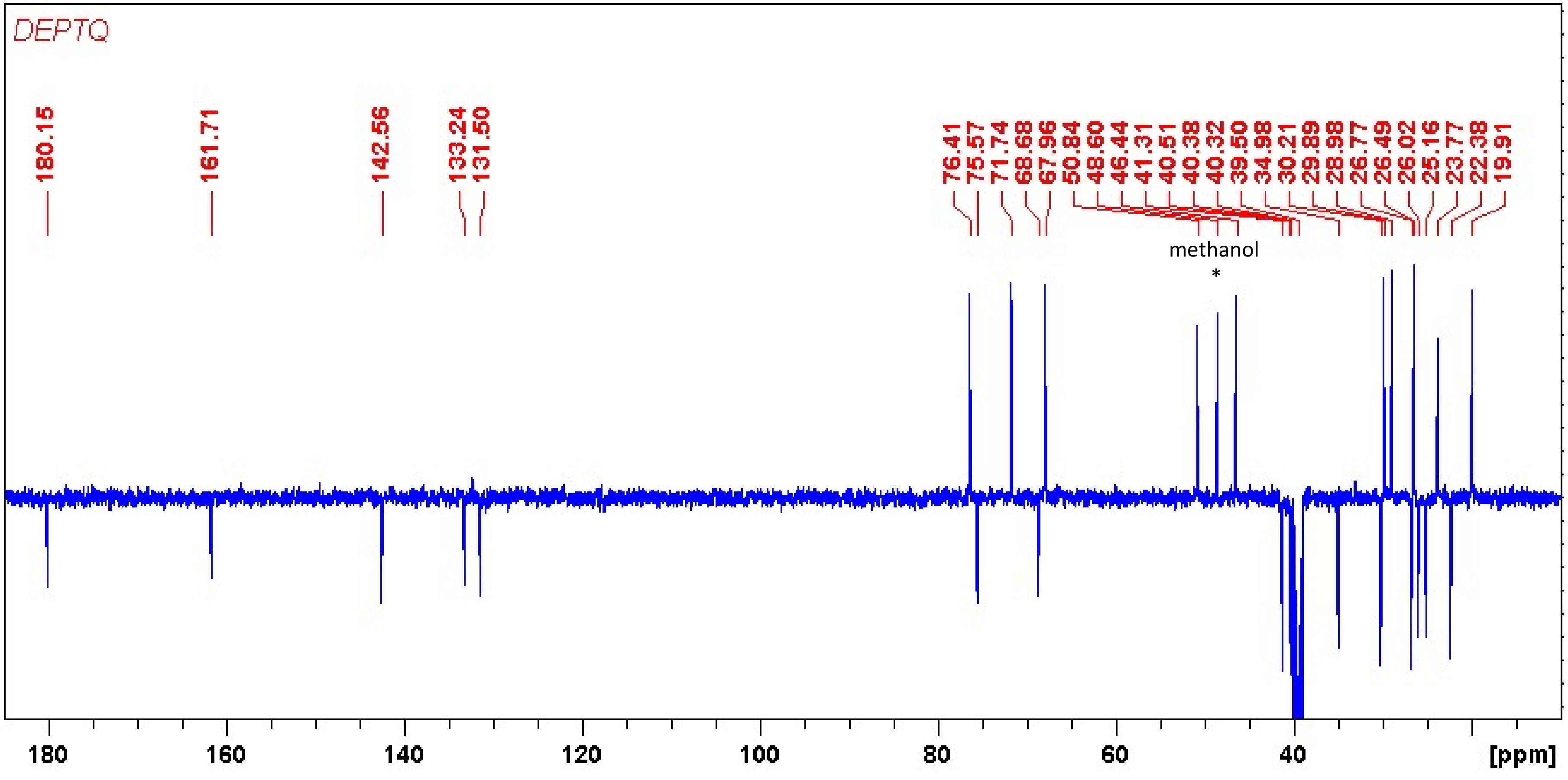

S27 Compound **4**

edHSQC

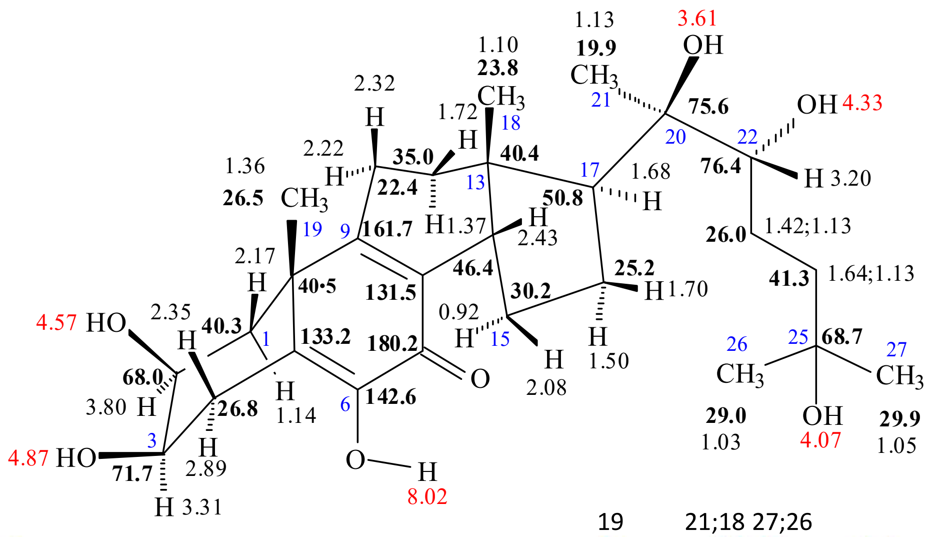

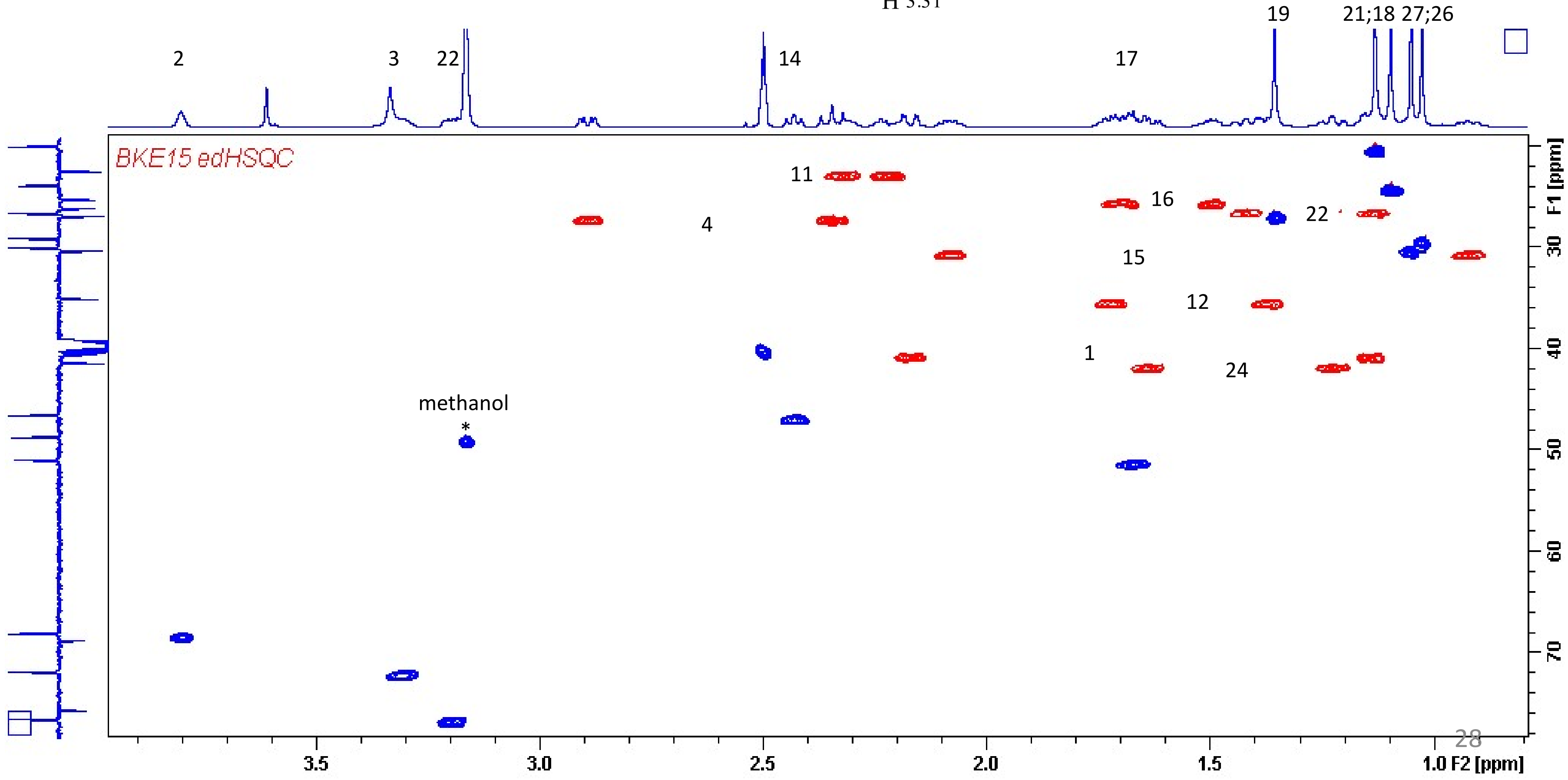

S28 Compound **4**

HMBC + selHMBC

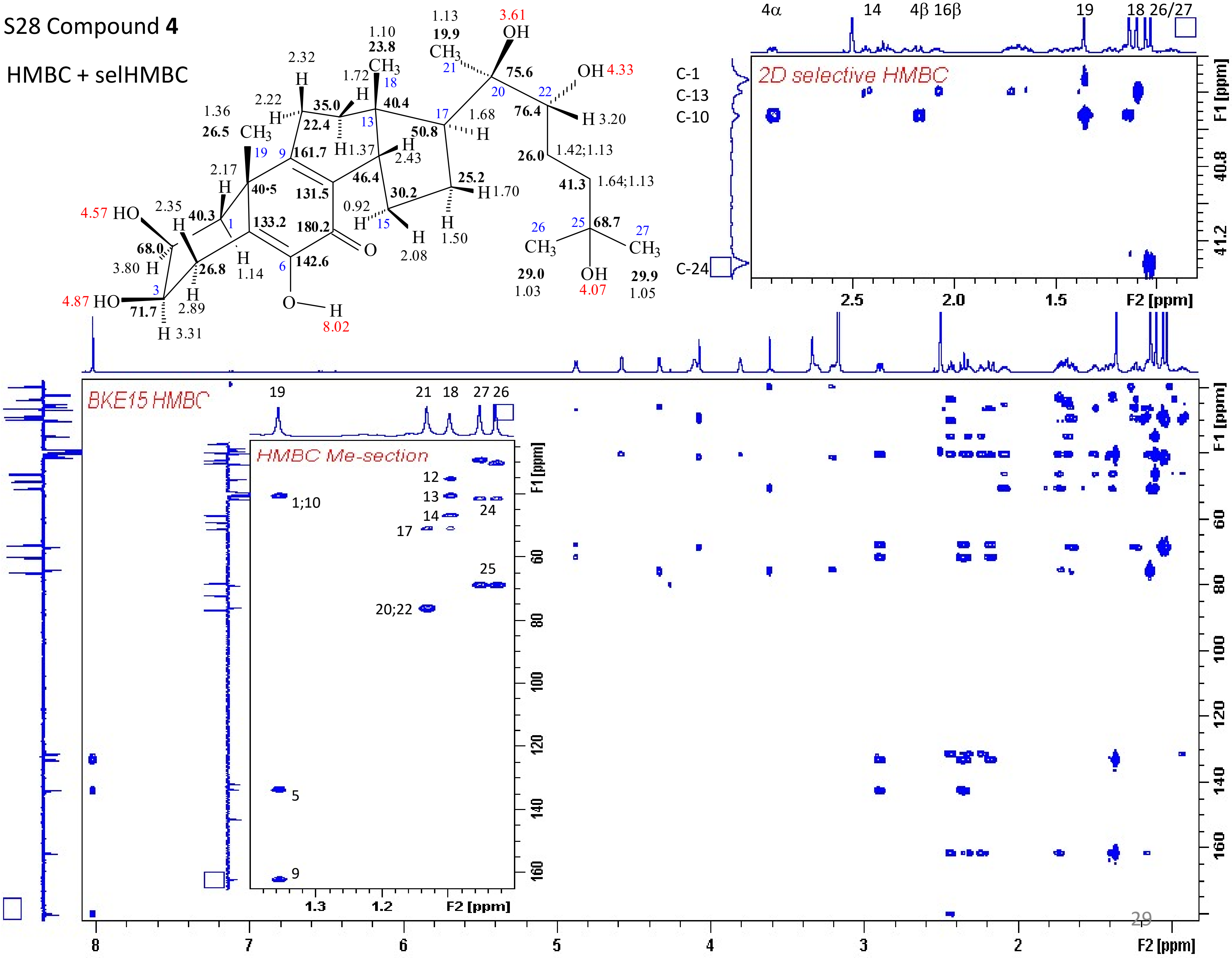

S29 Compound **5**

^{1}H 500 MHz CD_3OD

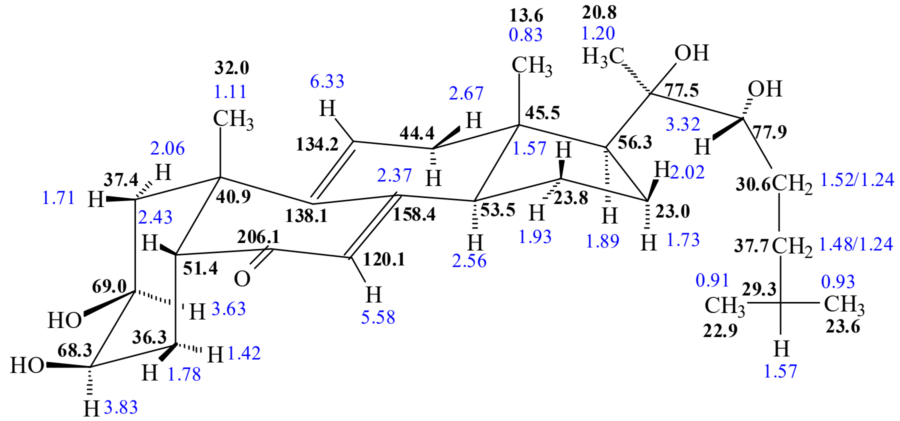

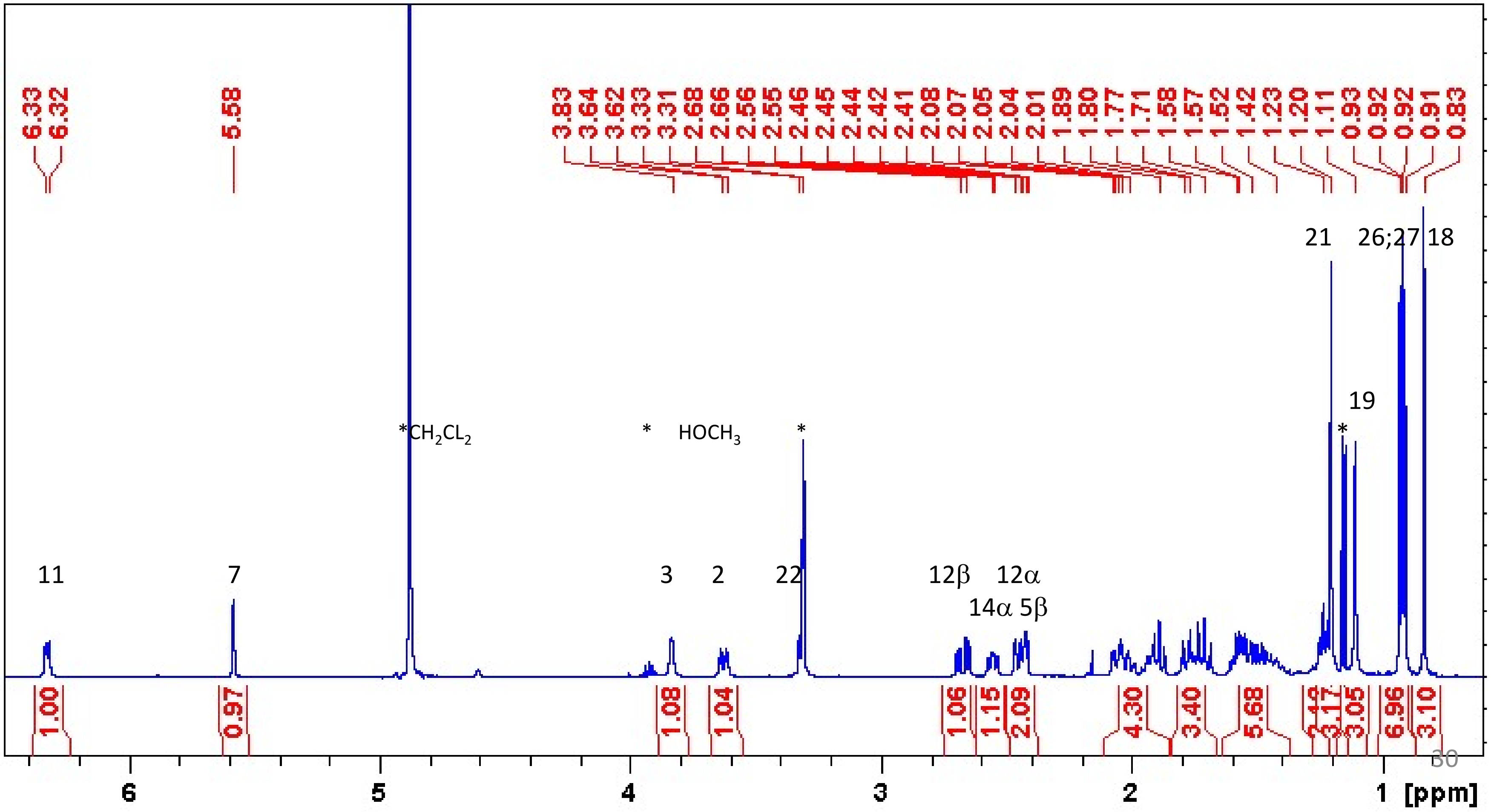

S30 Compound **5**

Identification of spin-systems by selTOCSY on H-11, H-2, Hα-14 and H-22

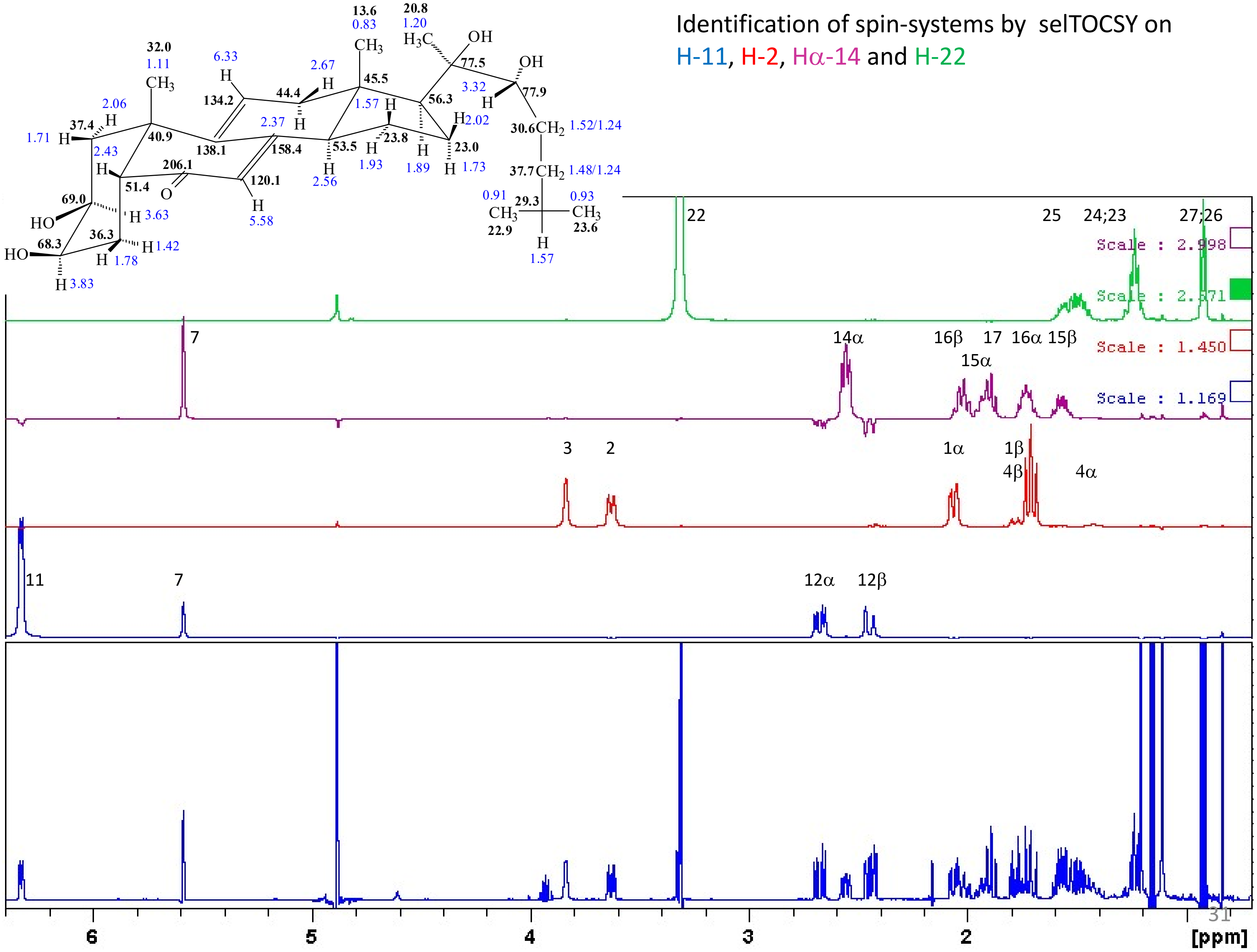

S31 Compound **5**

Steric proximities detected by selROE on Me-21, Me-19 and Me-18

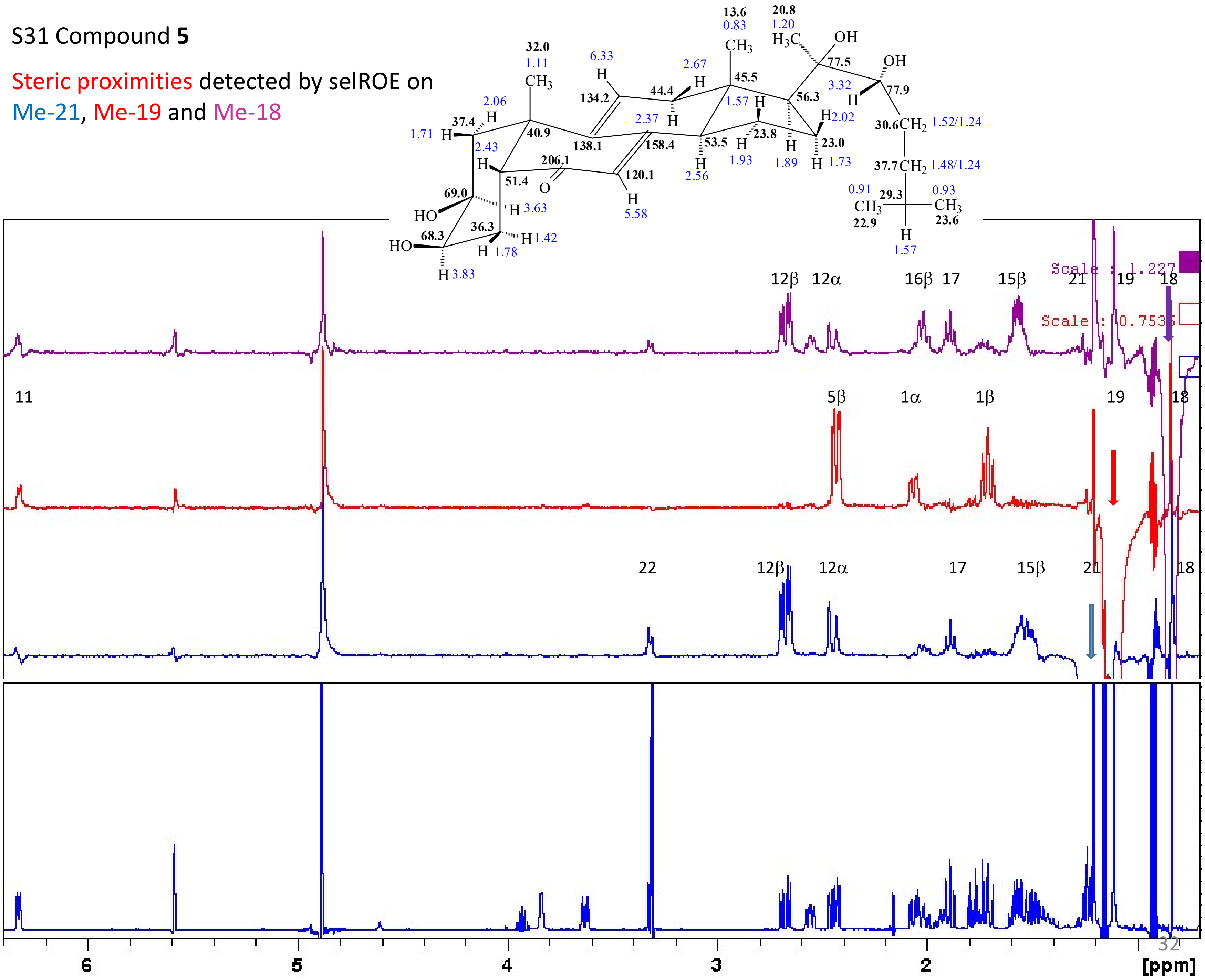

S32 Compound **5**

DEPTQ 125 MHz

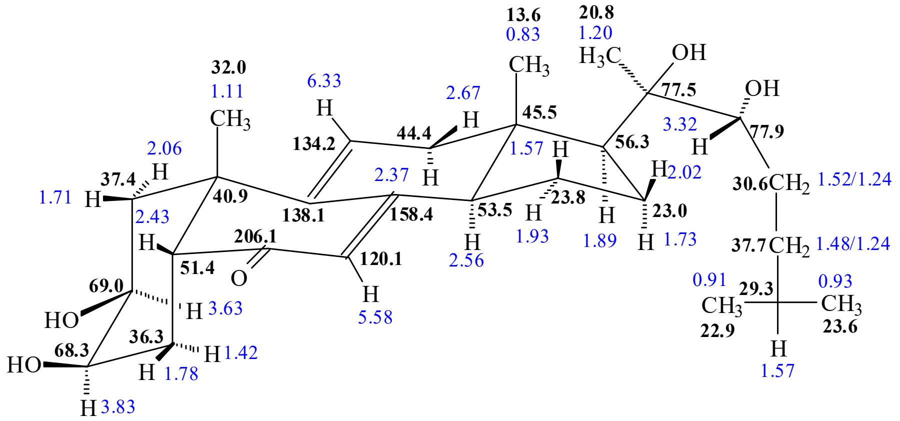

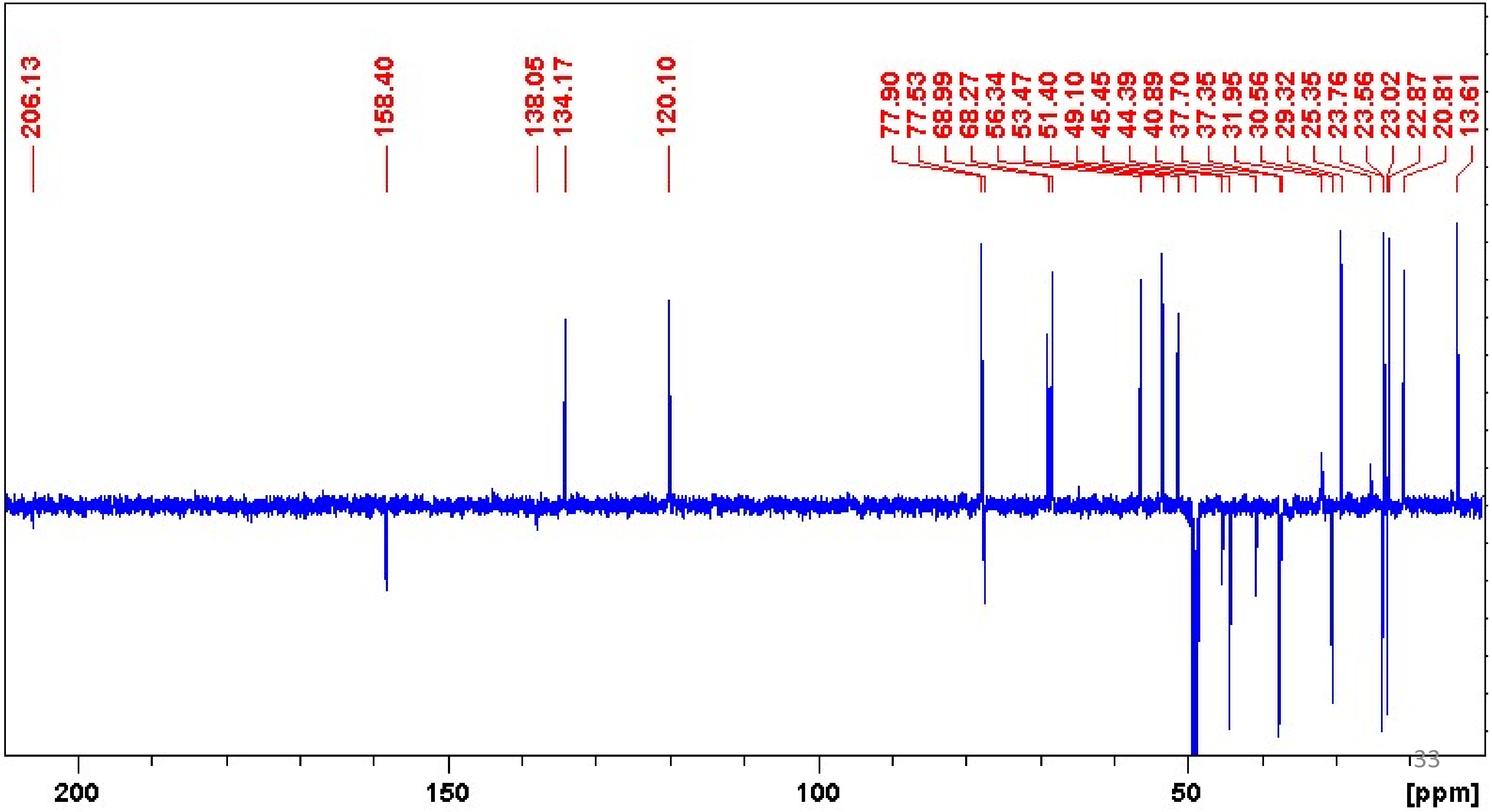

S33 Compound **5**

HSQC

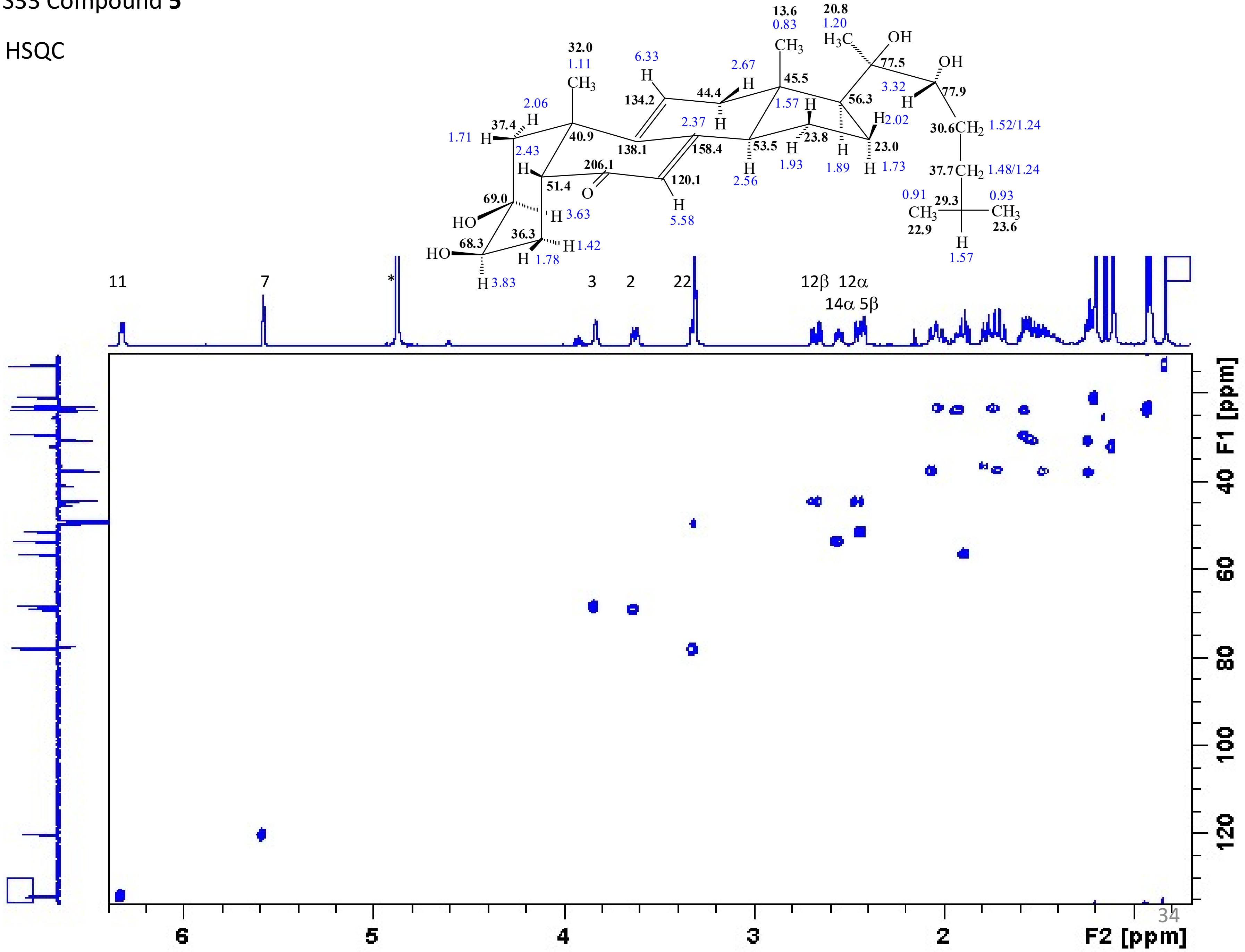

S34 Compound **5**

edHSQC CH_2 section

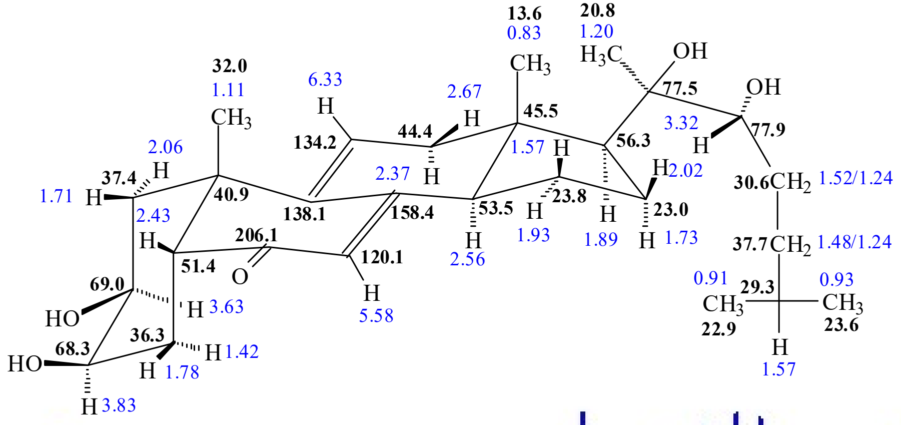

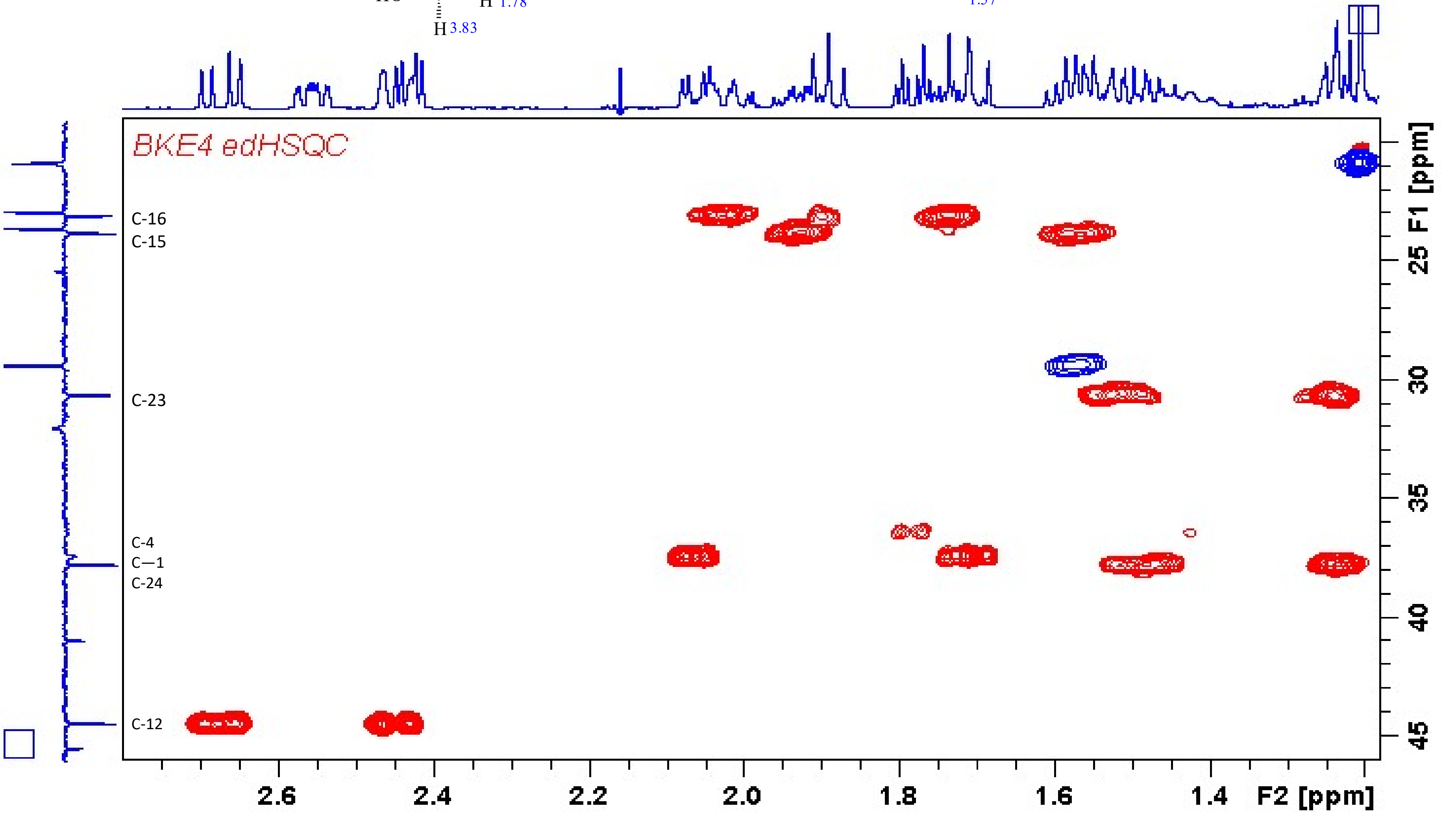

S35 Compound **5**

HMBC

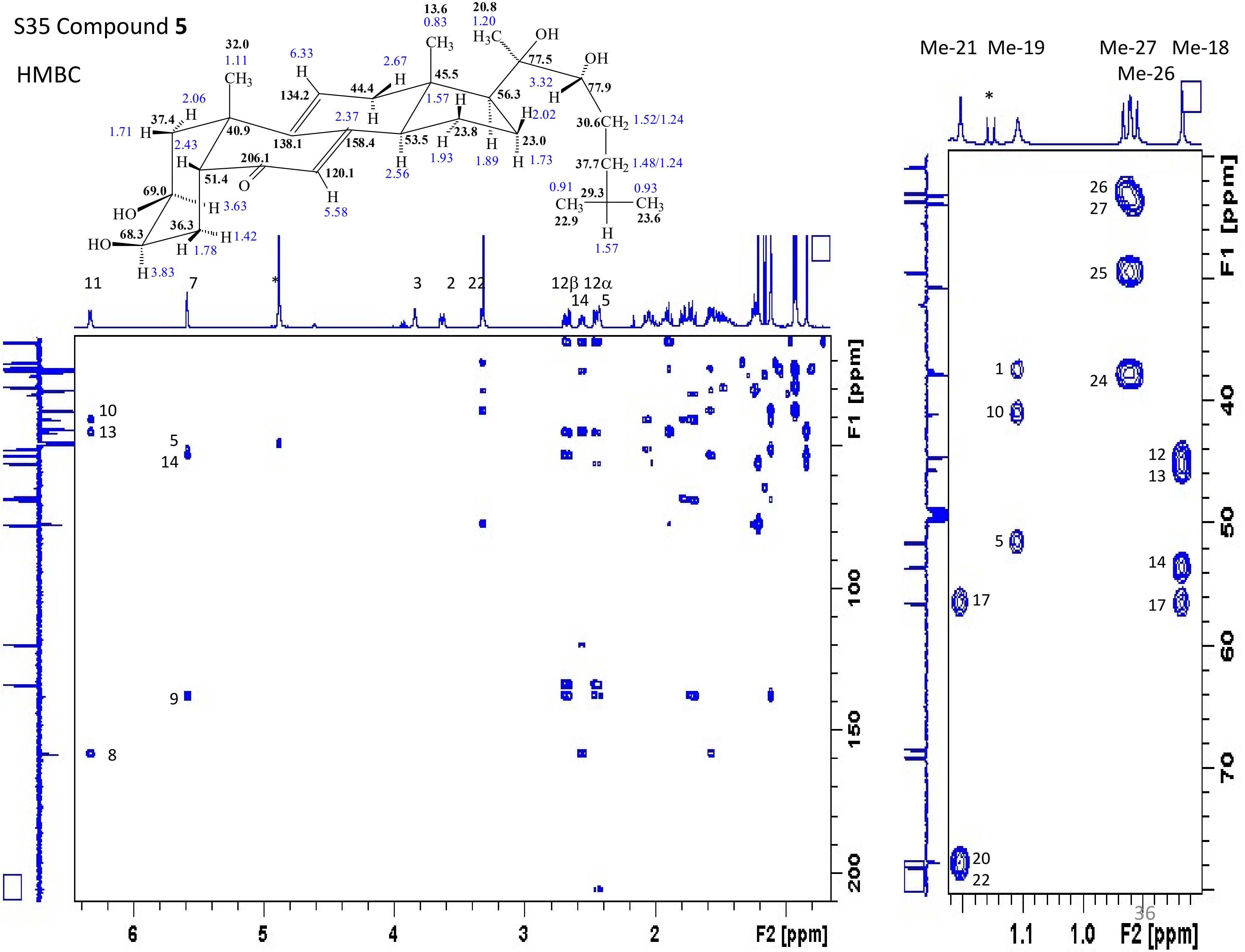

S36 Compound **6**

^{1}H 500 MHz CD_3OD

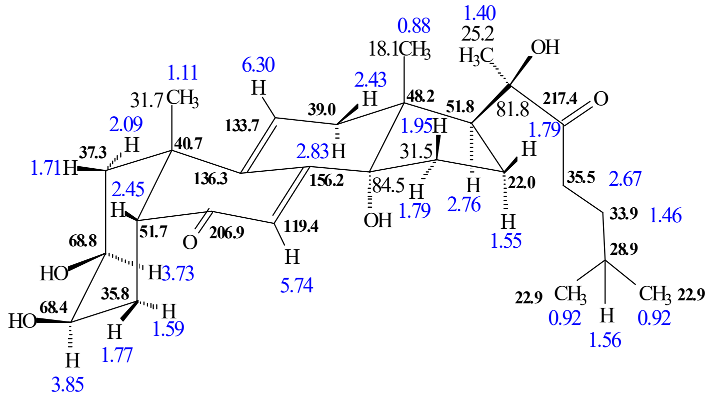

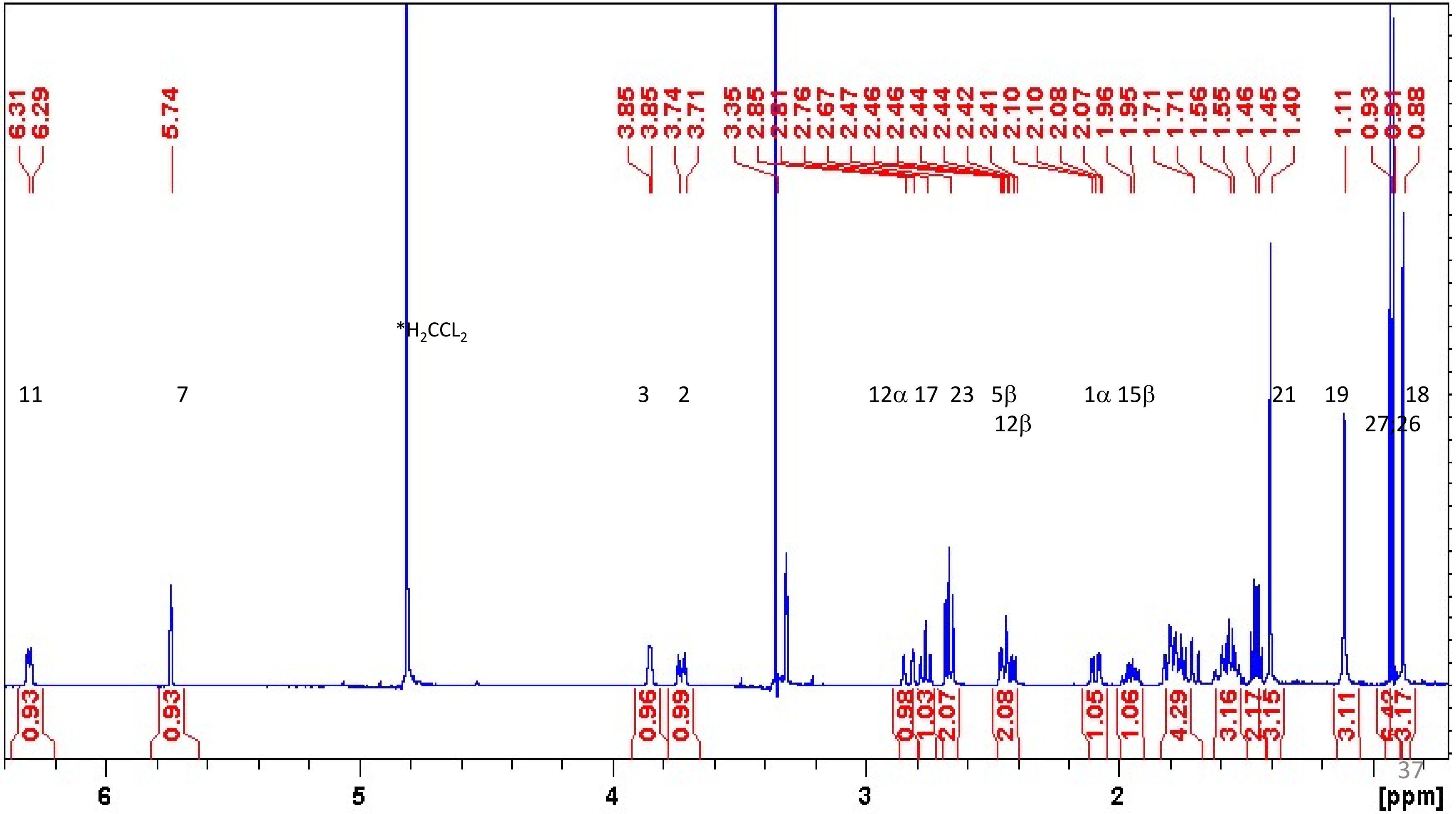

S37 Compound **6**

^{13}C + Dept-135 125 MHz

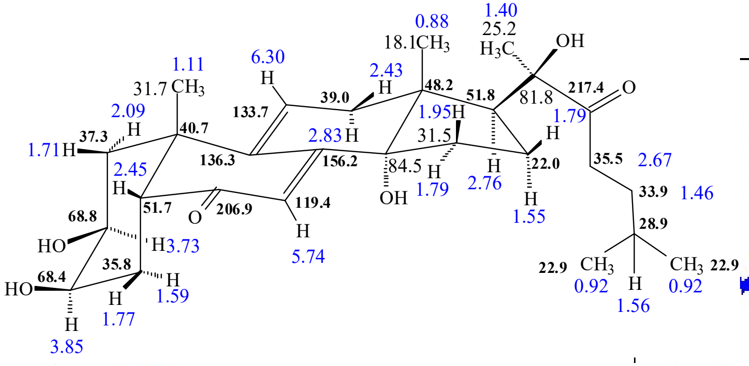

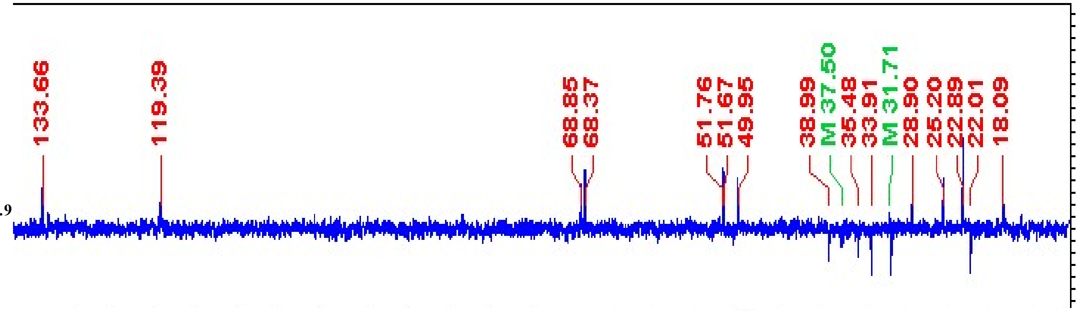

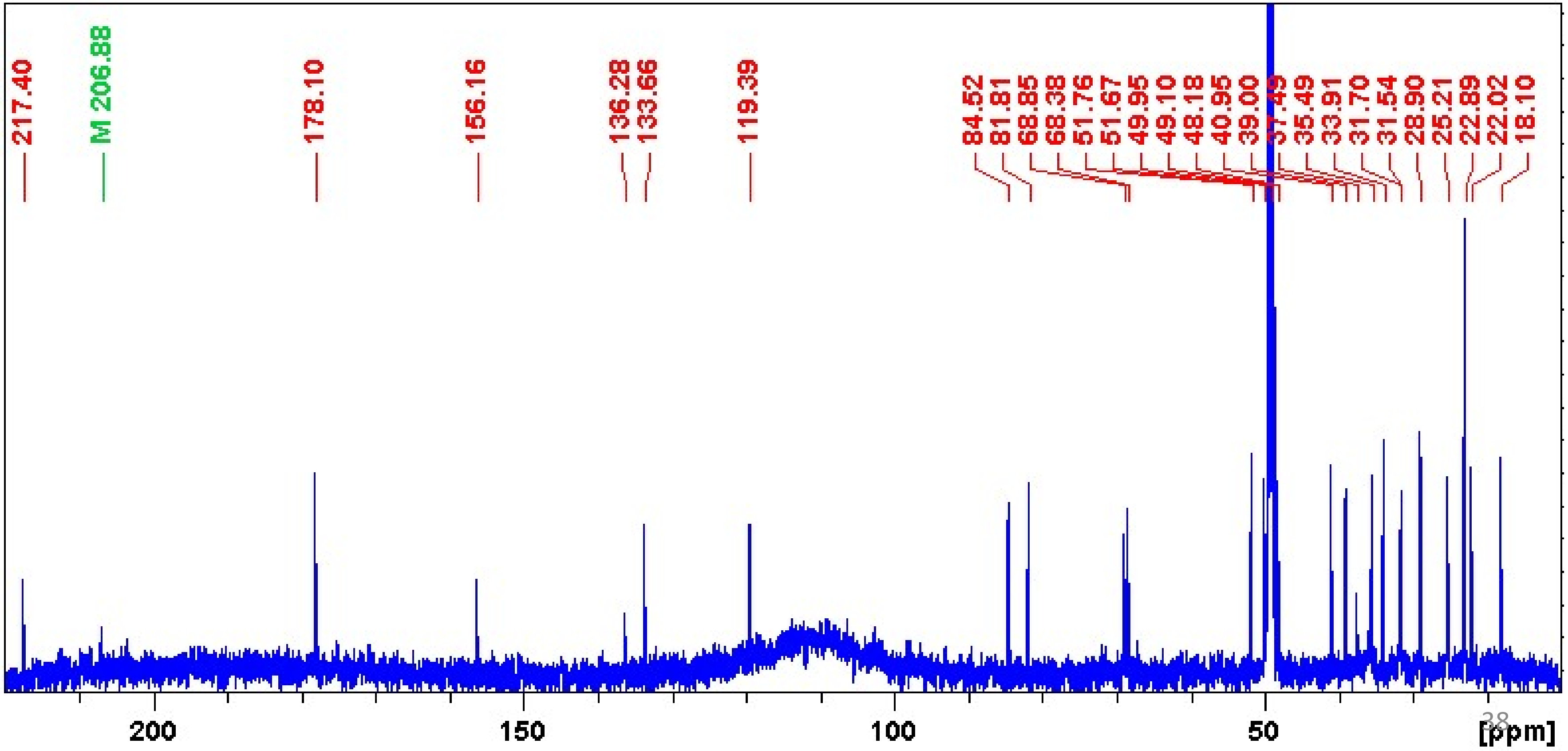

S38 Compound **6**

edHSQC + edHSQC CH_2 section

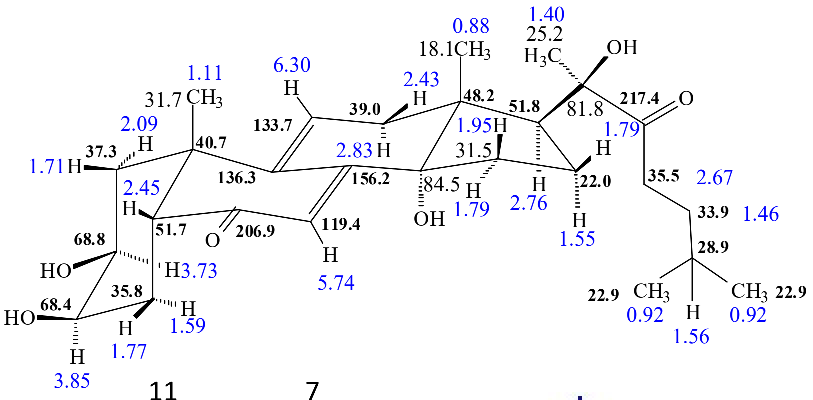

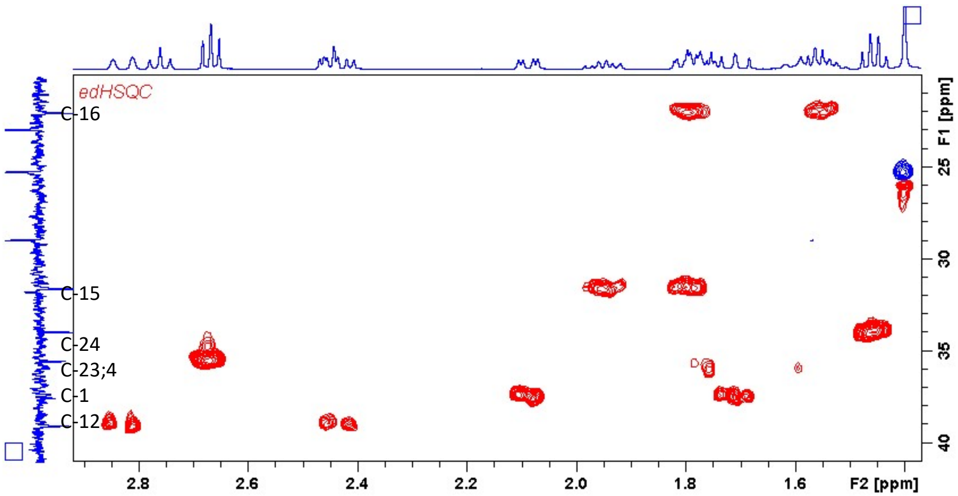

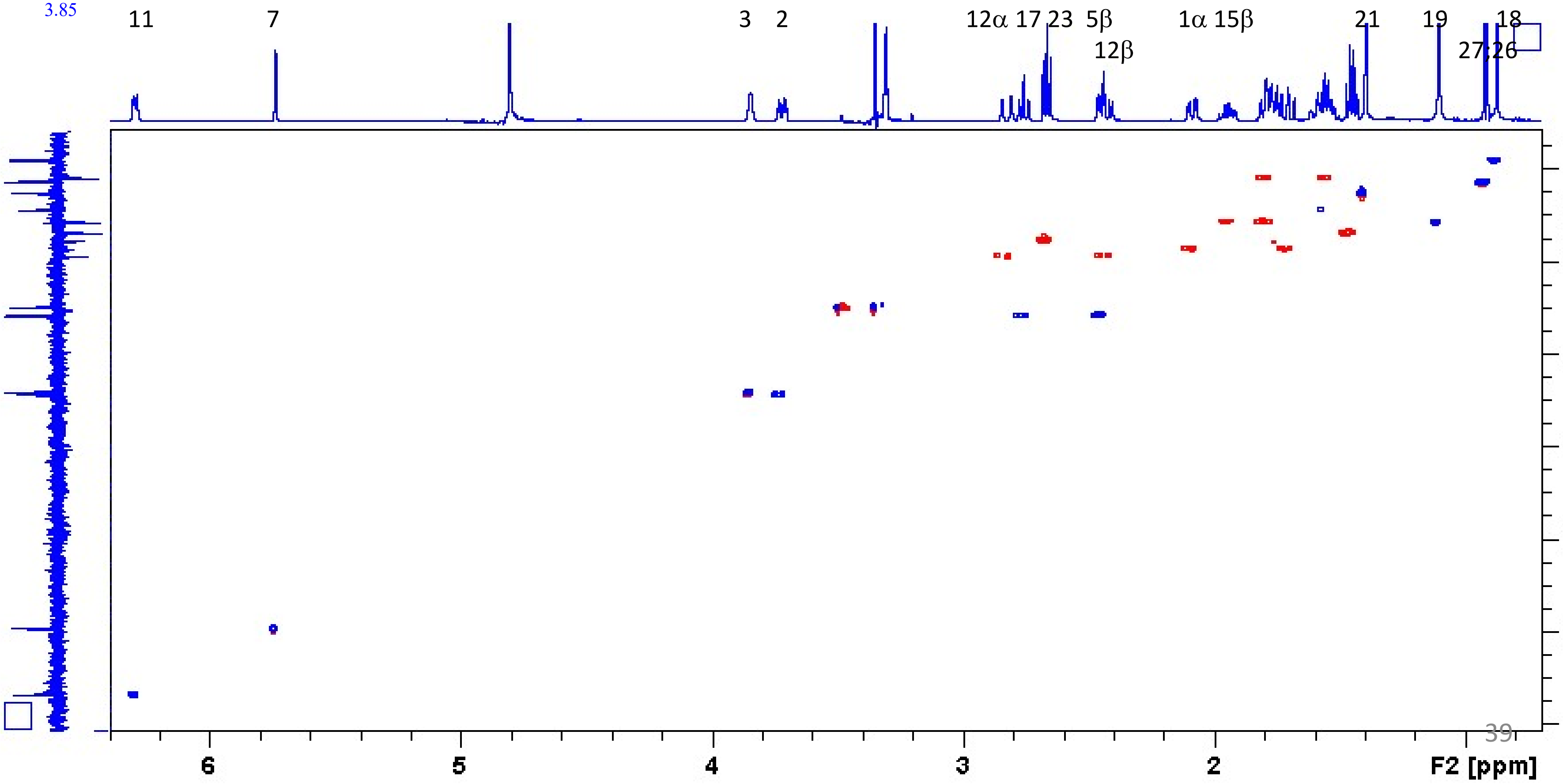

S39 Compound **6**

HMBC

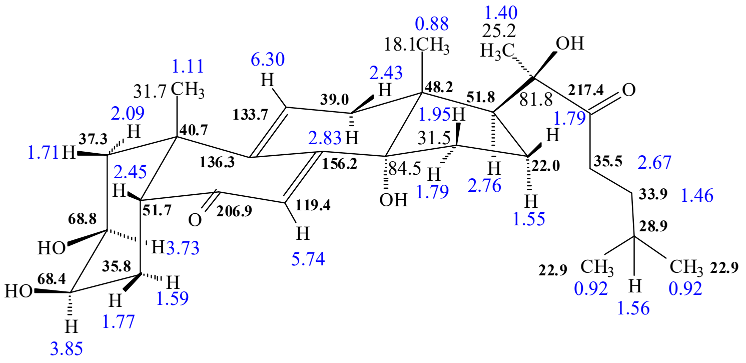

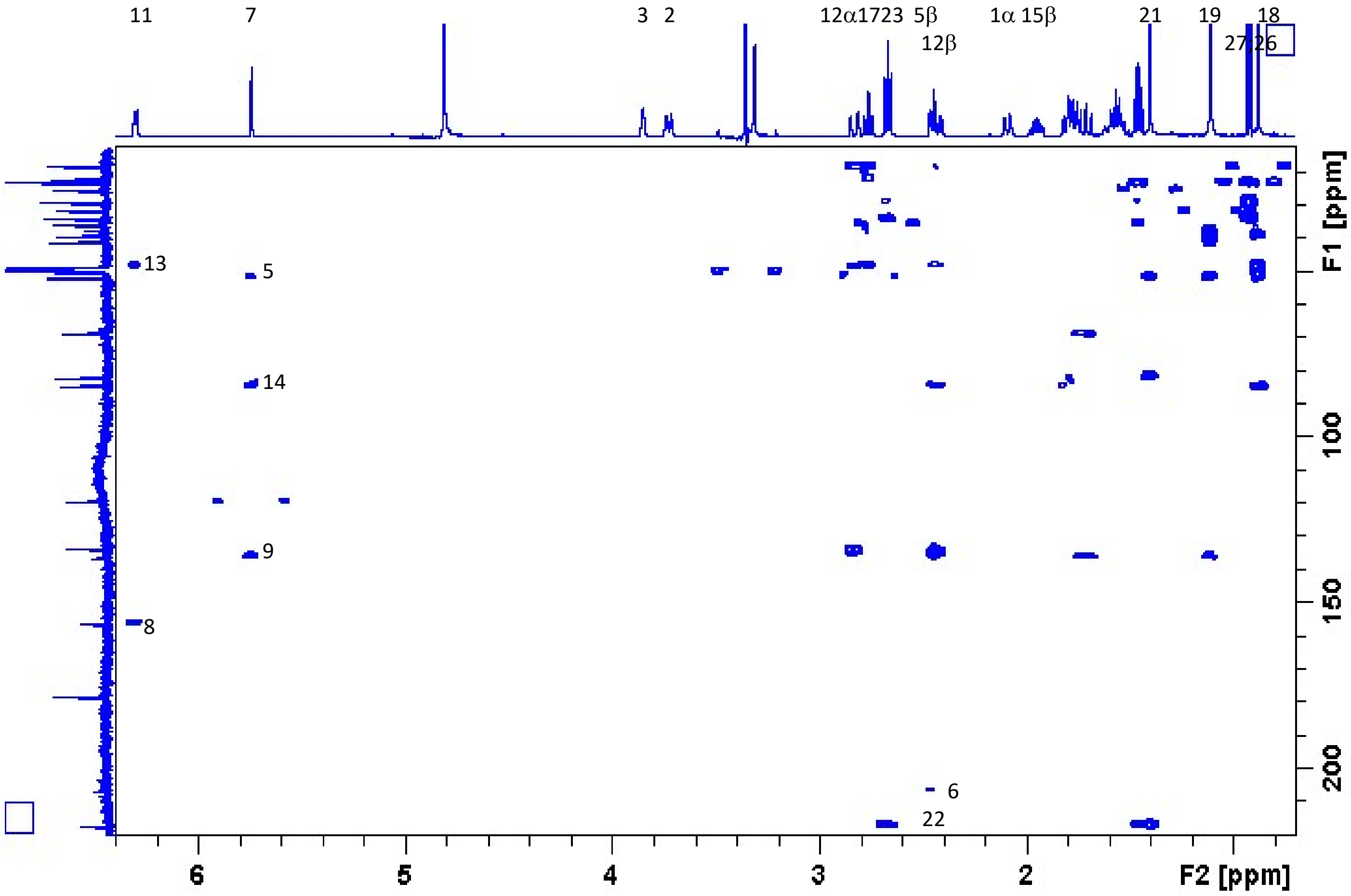

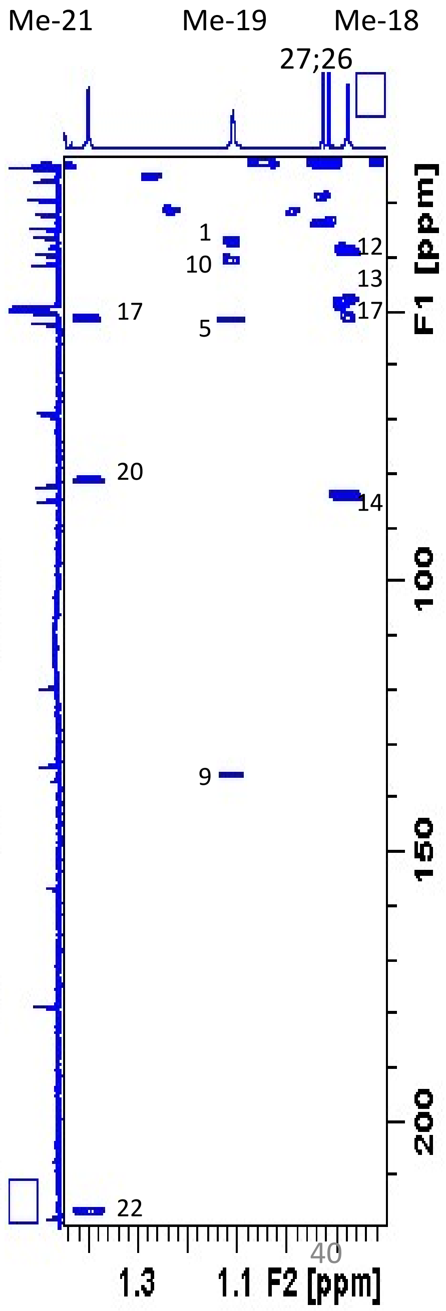

S40 Compound **6**

2D-NOESY

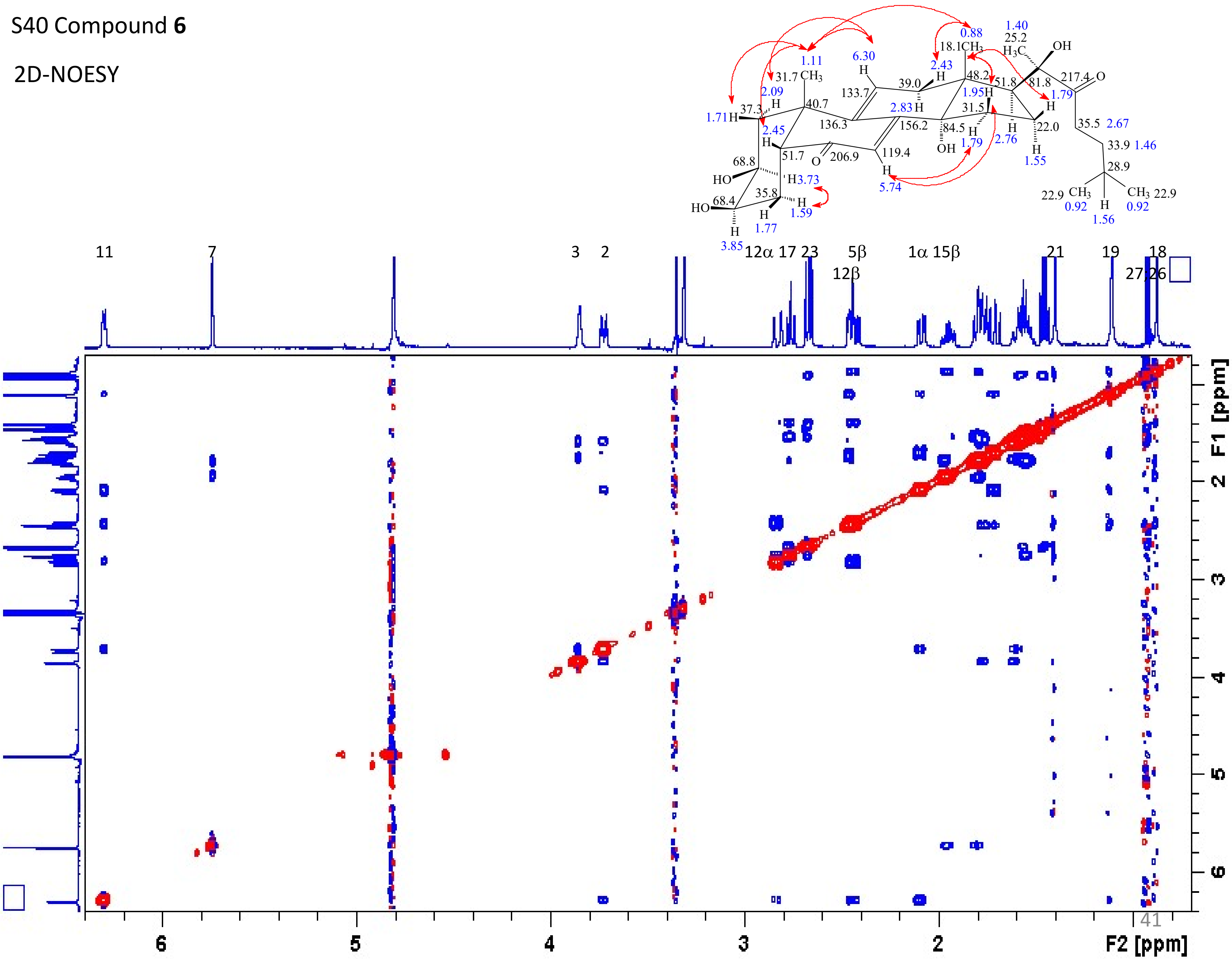

S41 Compound **7**

^{1}H 500 MHz

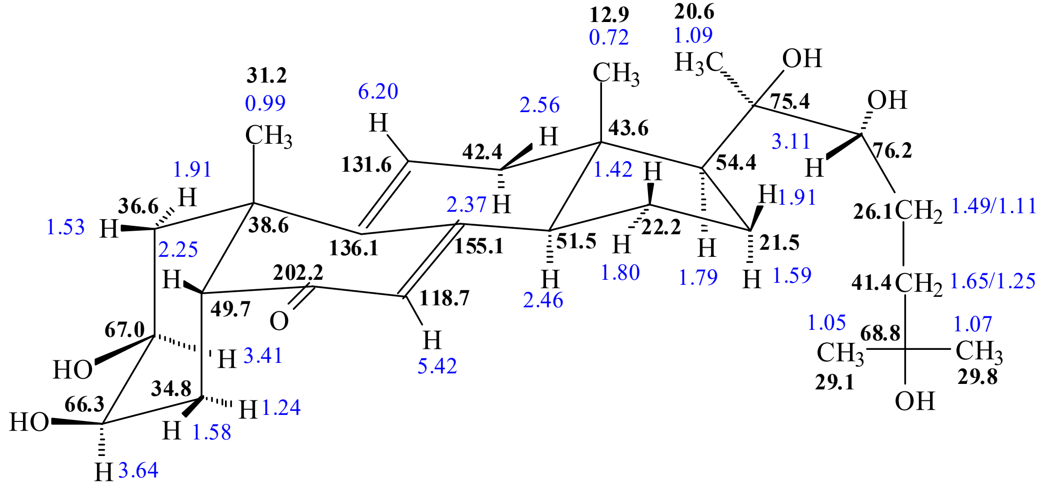

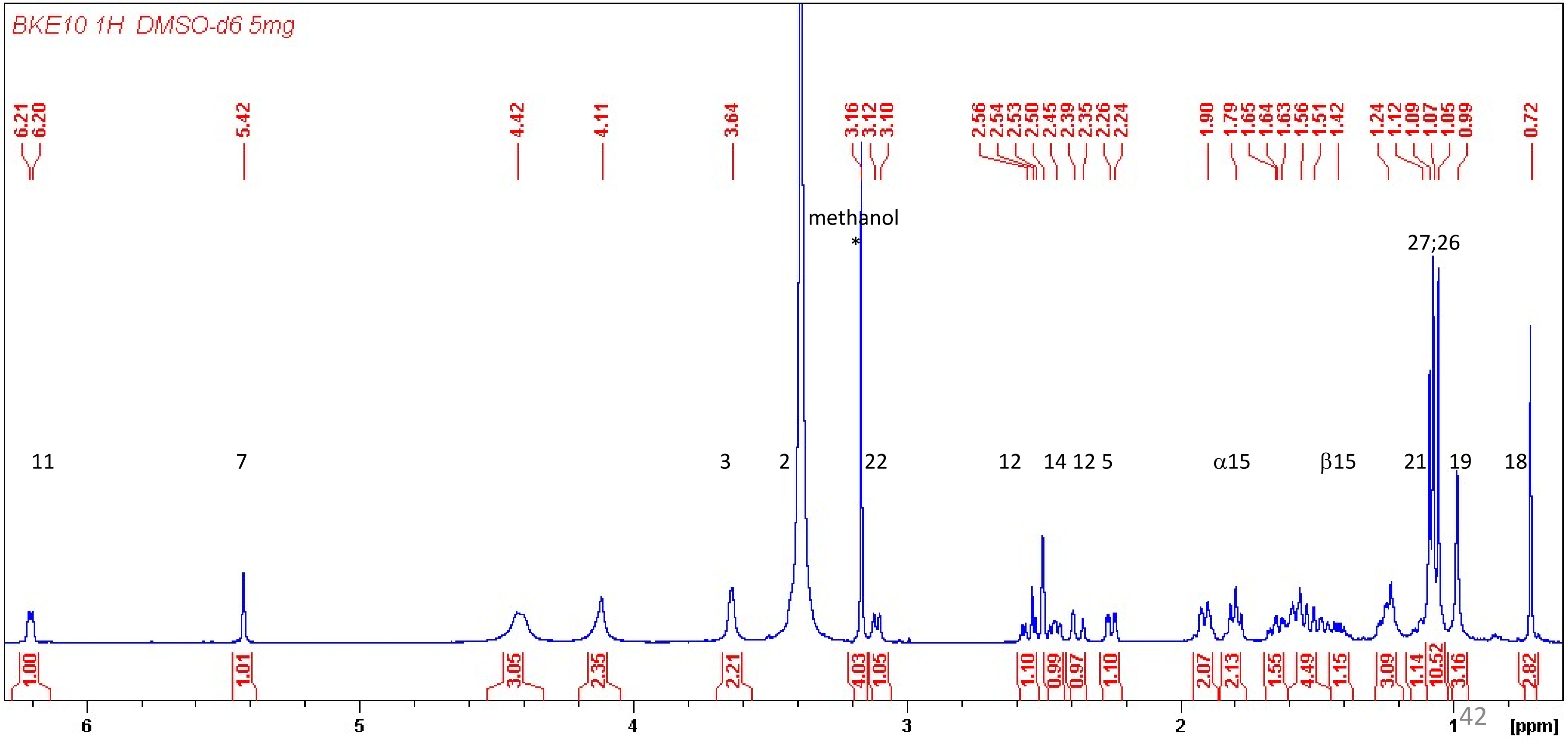

S42 Compound **7**

^{1}H,^{1}H-COSY 500 MHz

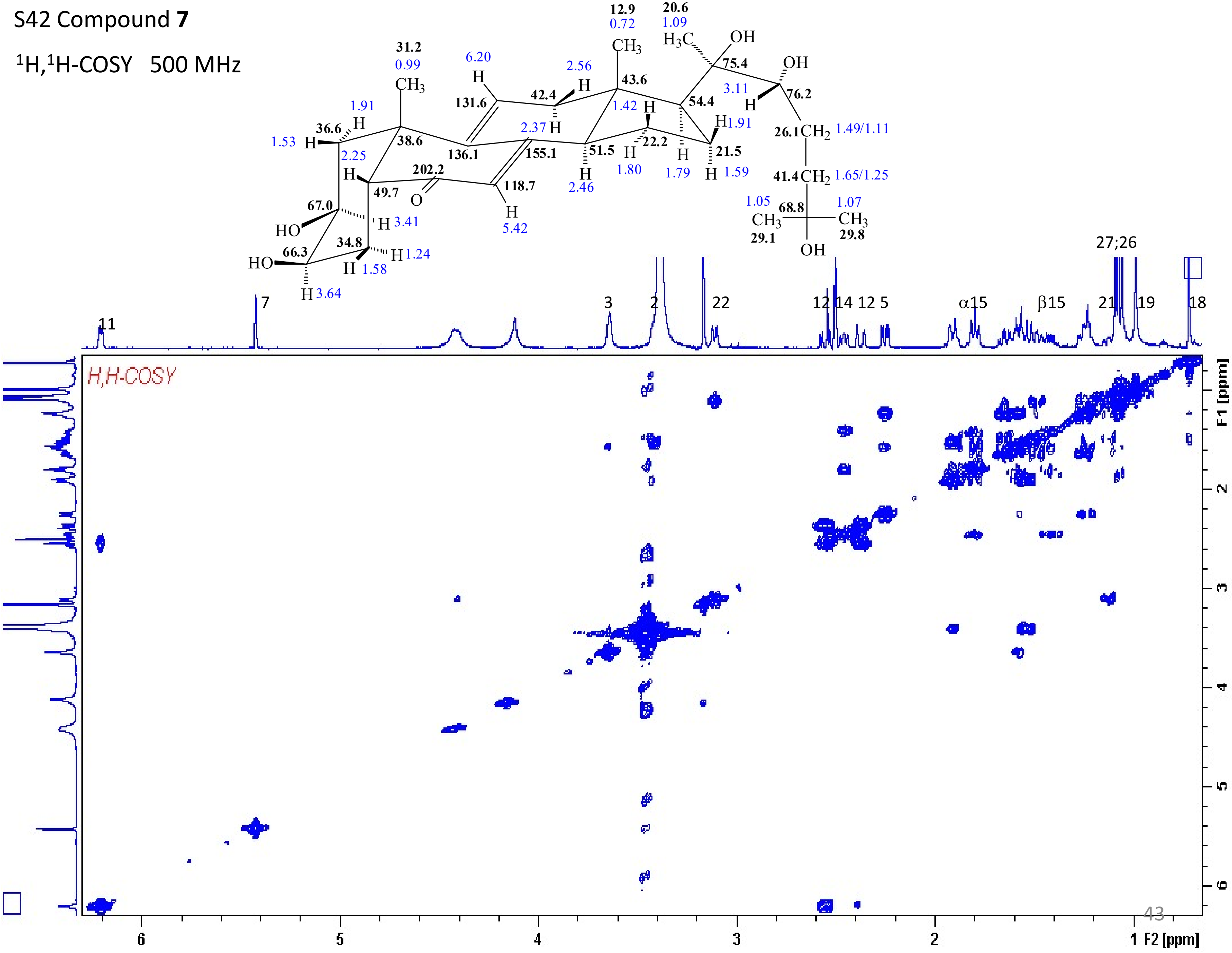

S43 Compound **7**

Steric proximities detected by sel-Roesy (τ_{mix}: 300 ms) on Me-19 and Me-18

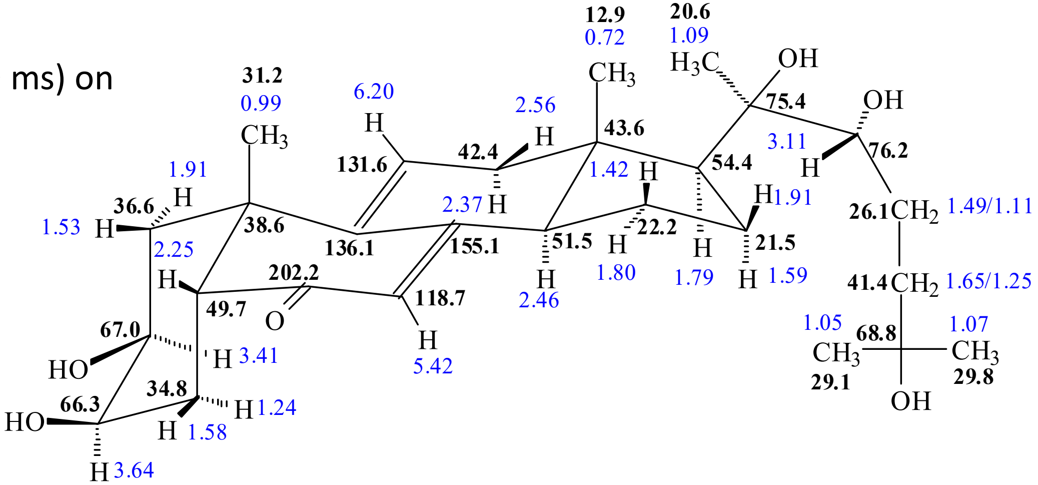

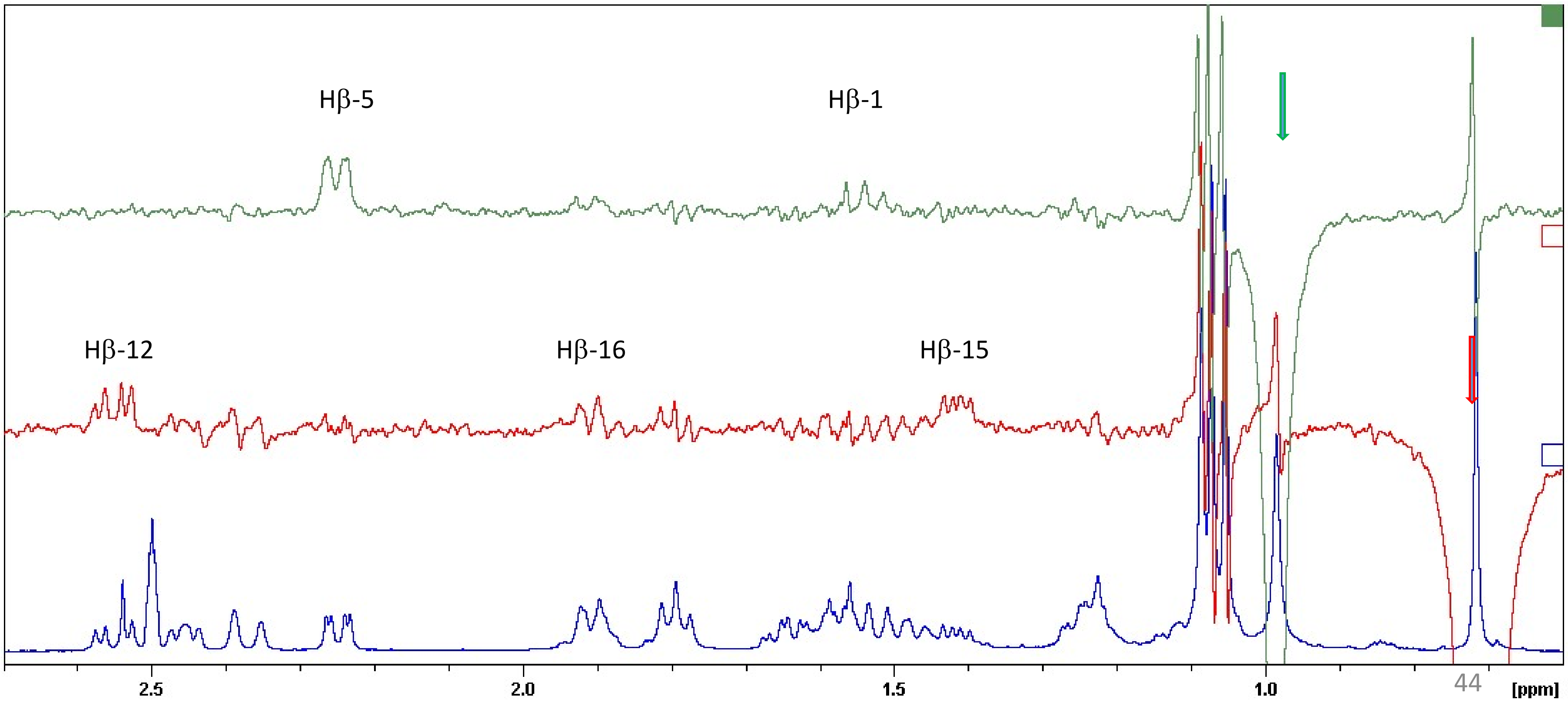

S44 Compound **7**

^{13}C 125 MHz

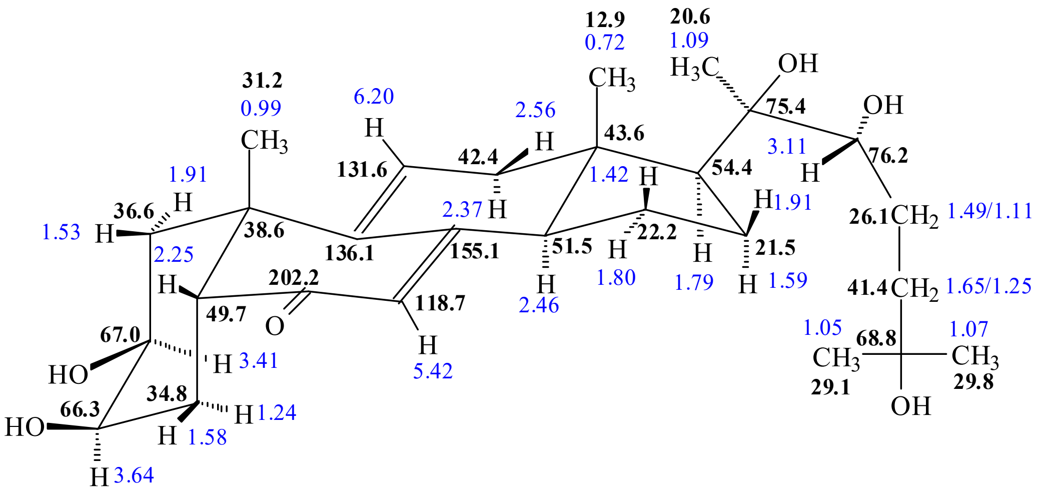

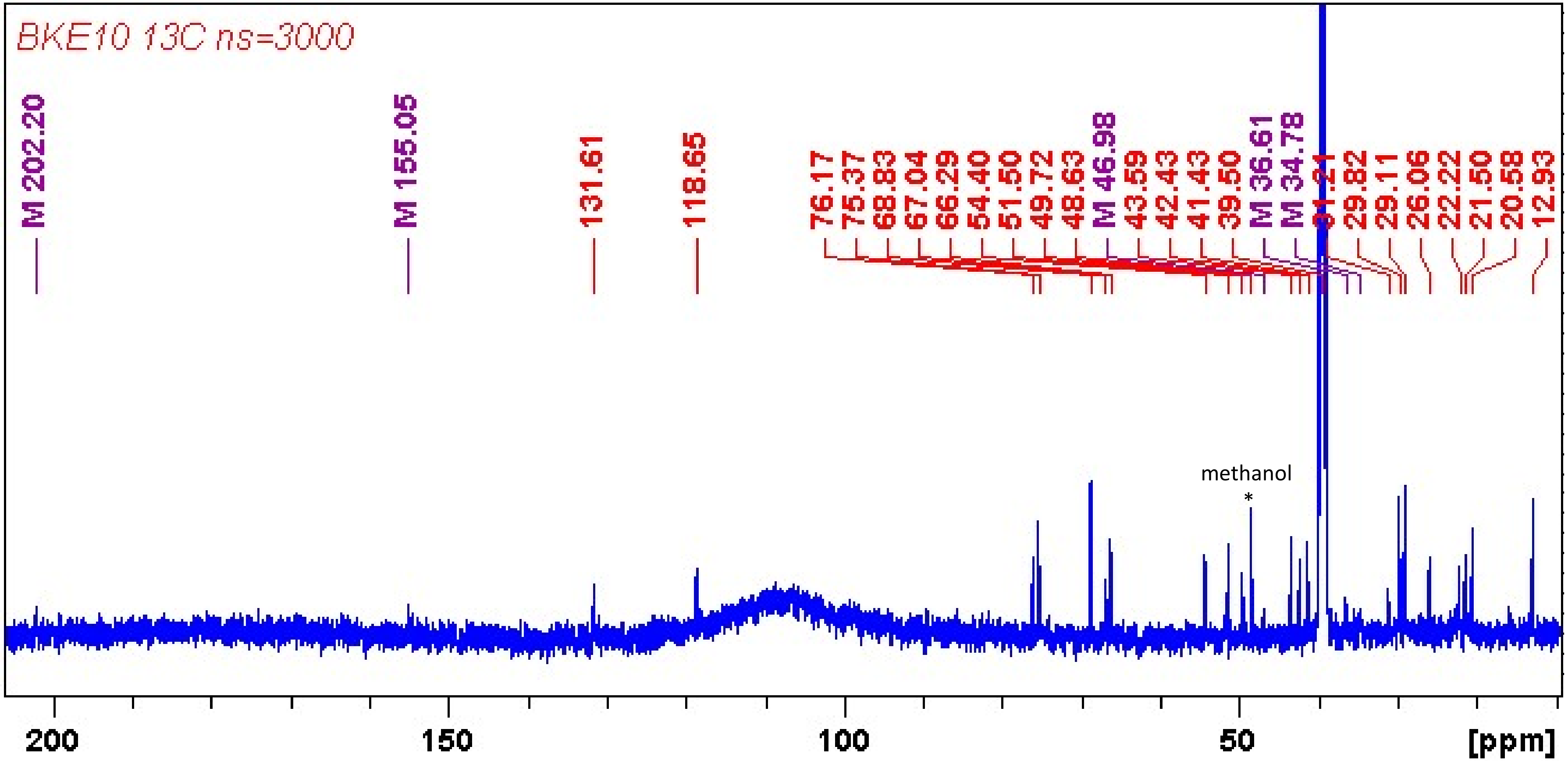

S45 Compound **7**

edHSQC

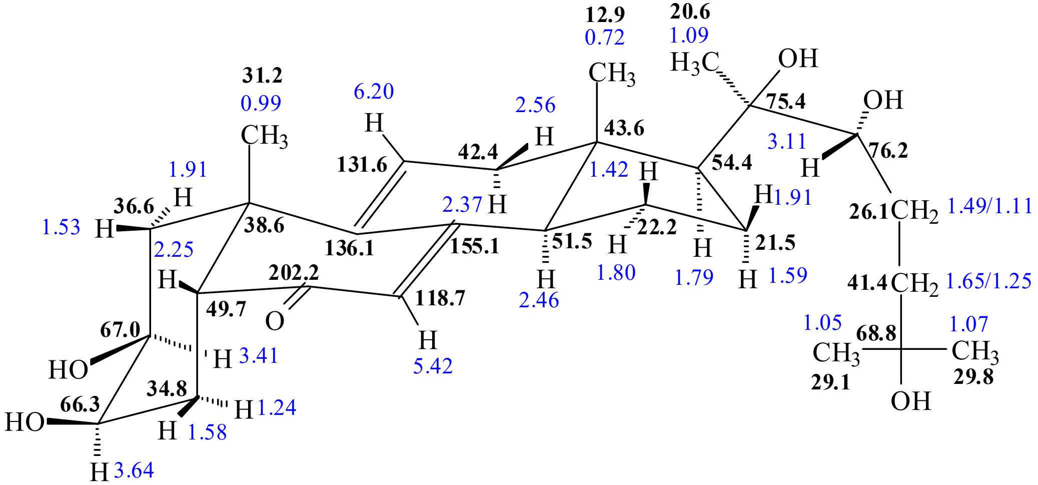

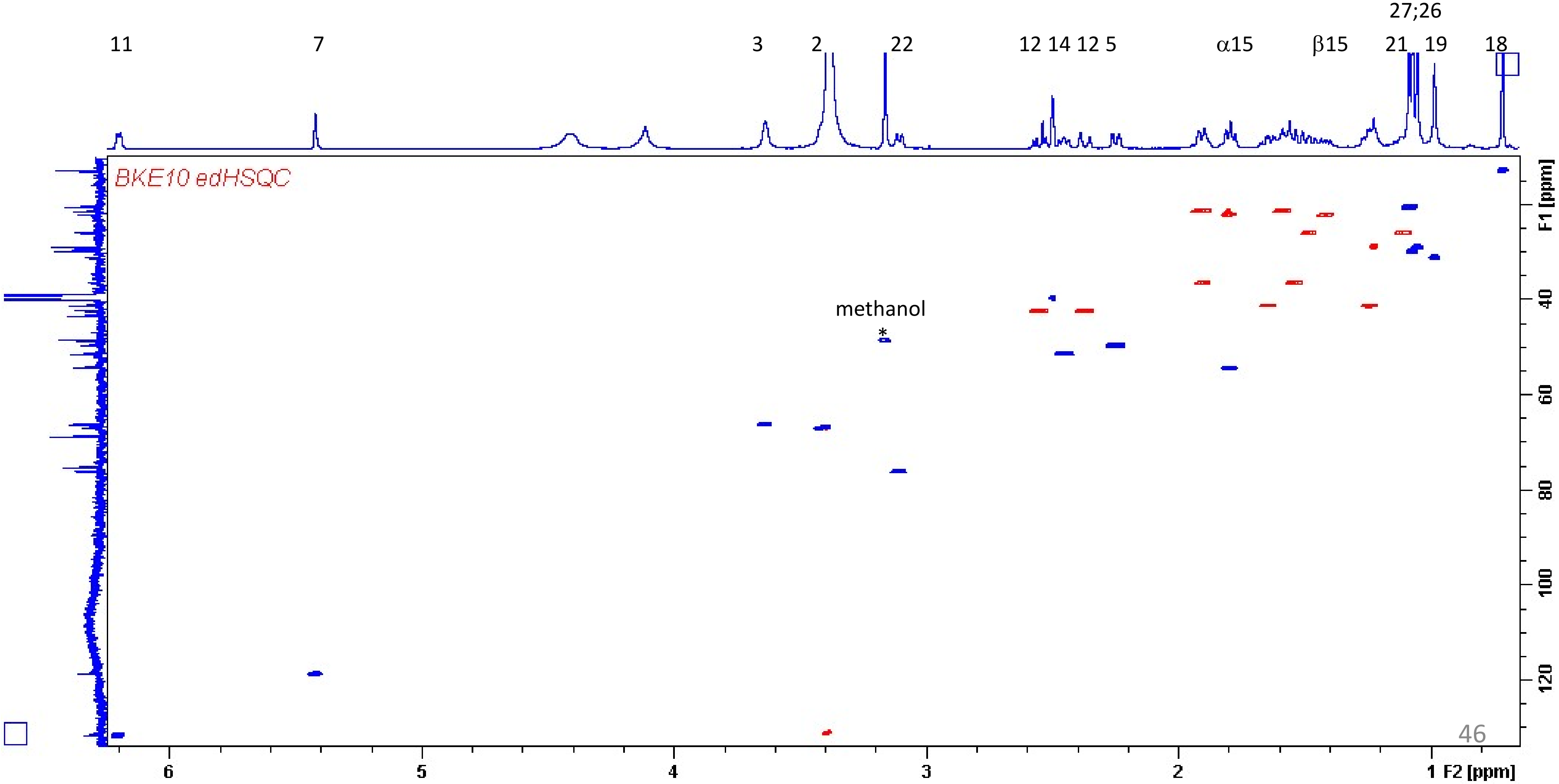

S46 Compound **7**

edHSQC sections: ^{1}H 2.6-0.94; ^{13}C 55-19 and 37-30 ppm

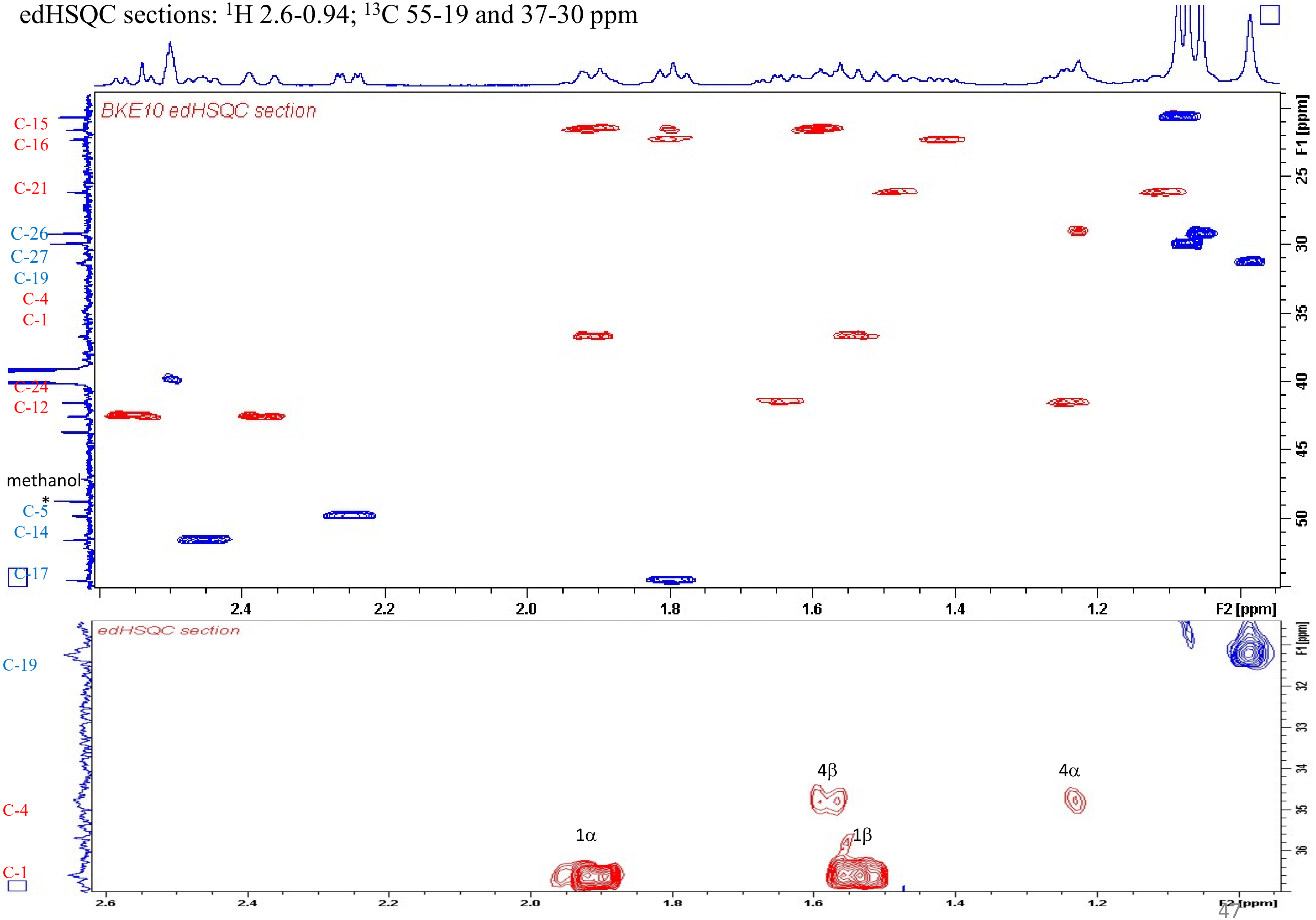

S47 Compound **7**

HMBC + Me-section

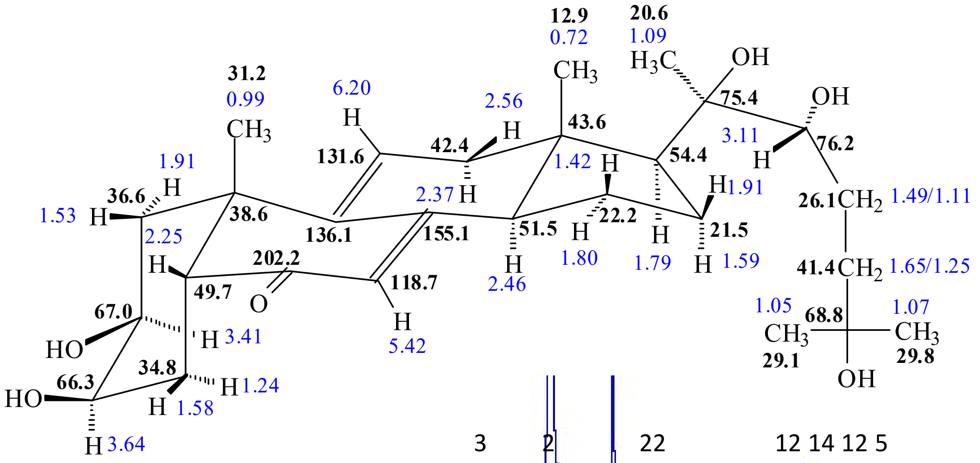

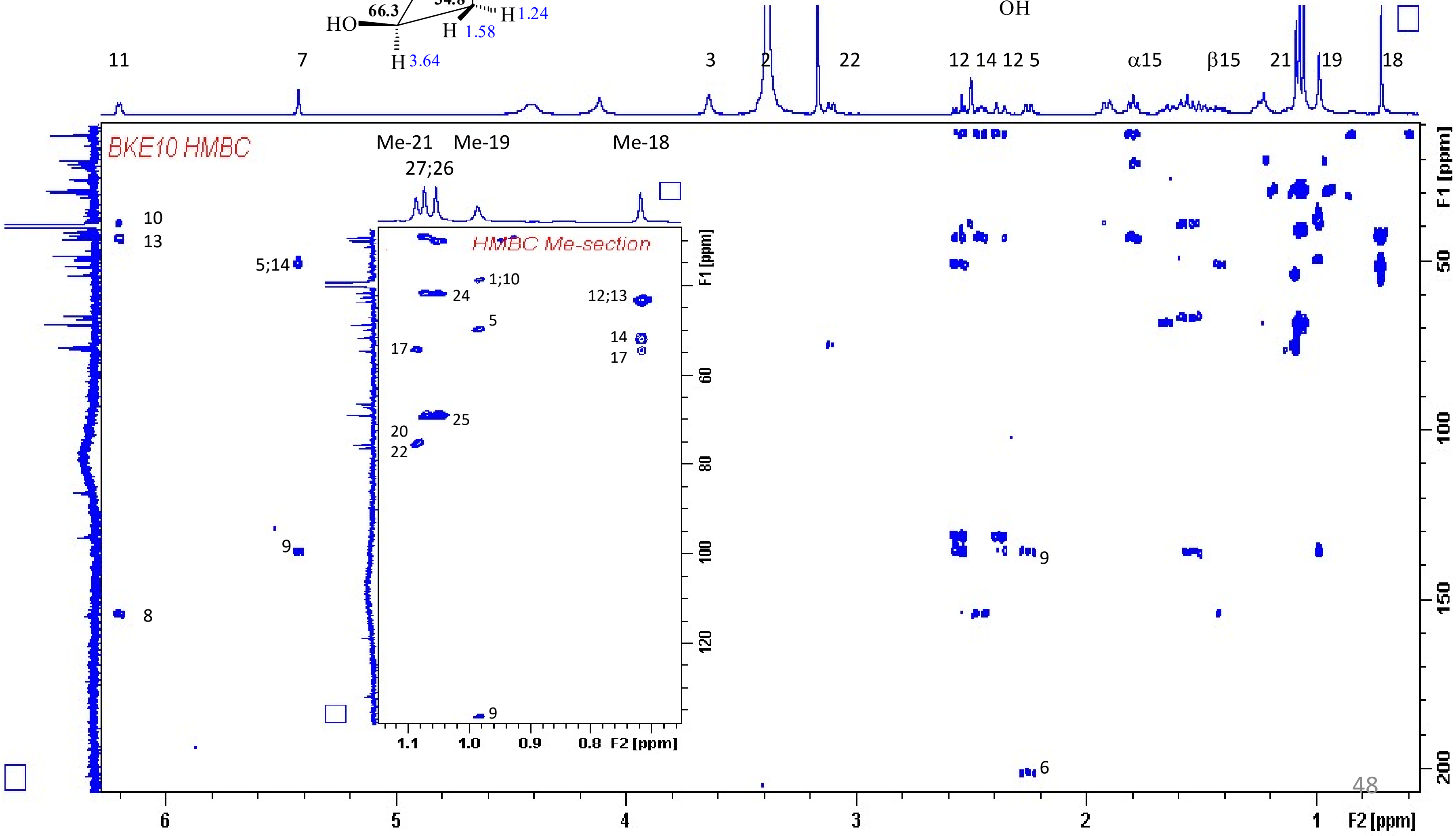

S48 Compound **8**

^{1}H 500 MHz

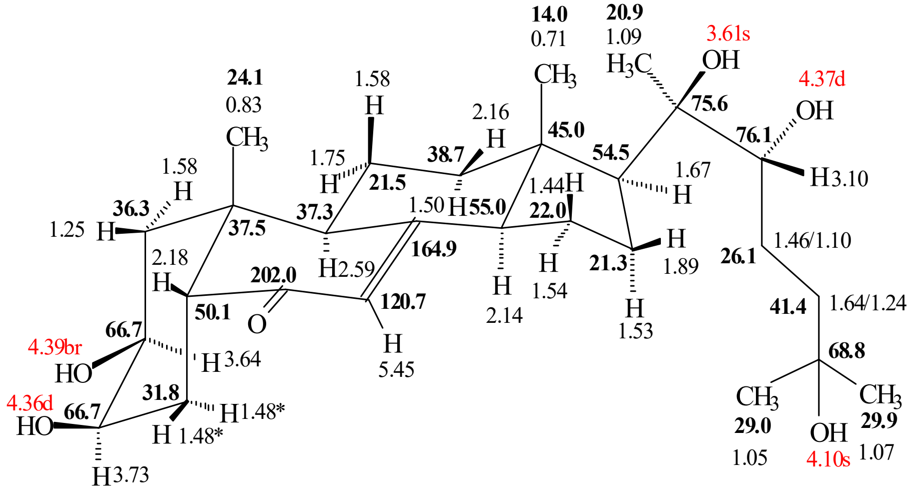

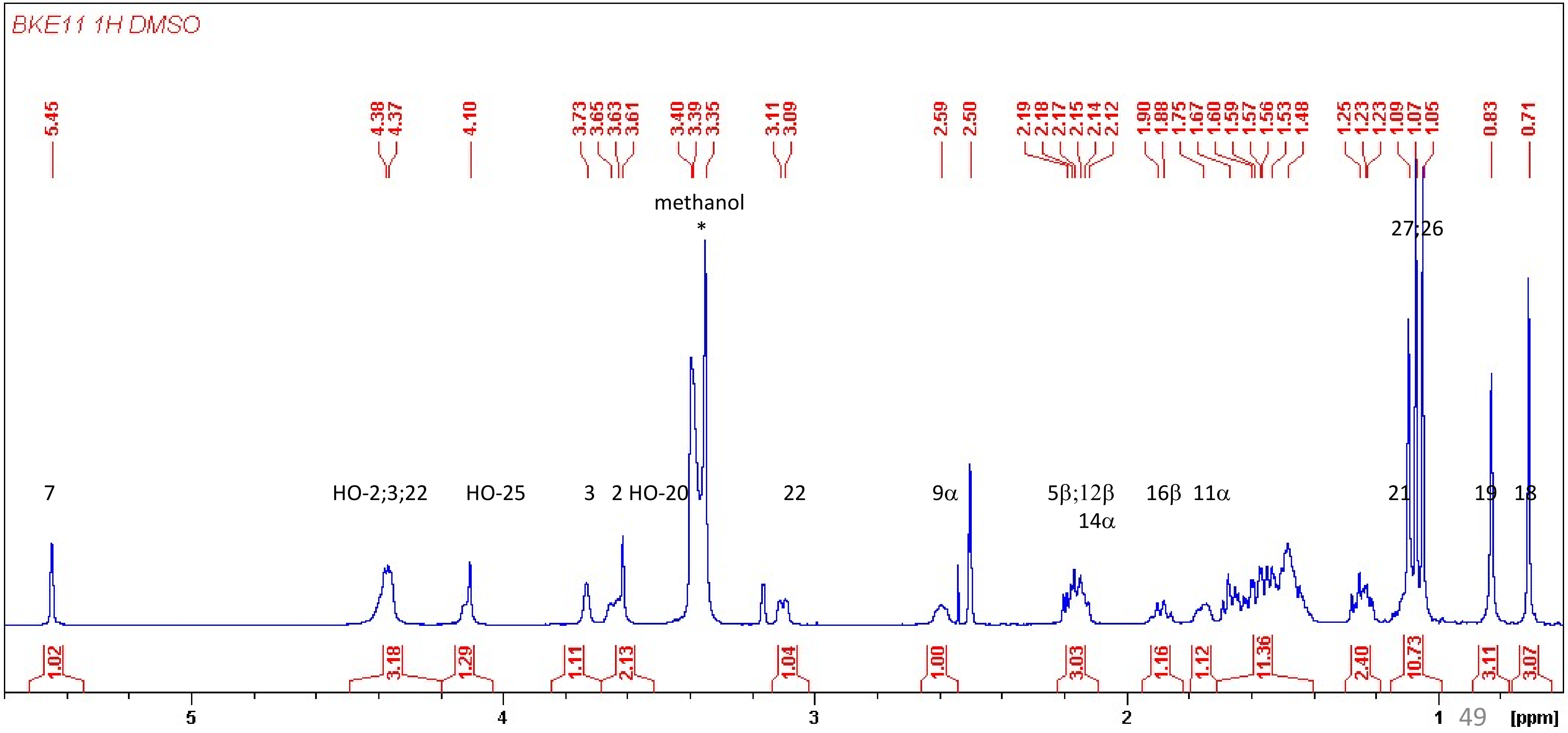

S49 Compound **8**

Identification of spin-systems by selTOCSY on Hα-11 and Hβ-16

Steric proximities detected by selROE on Me-18 and Me-19

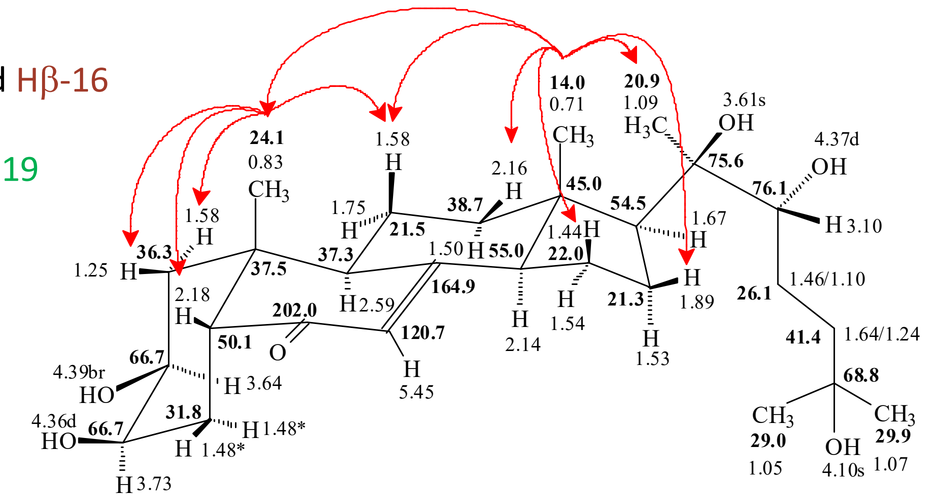

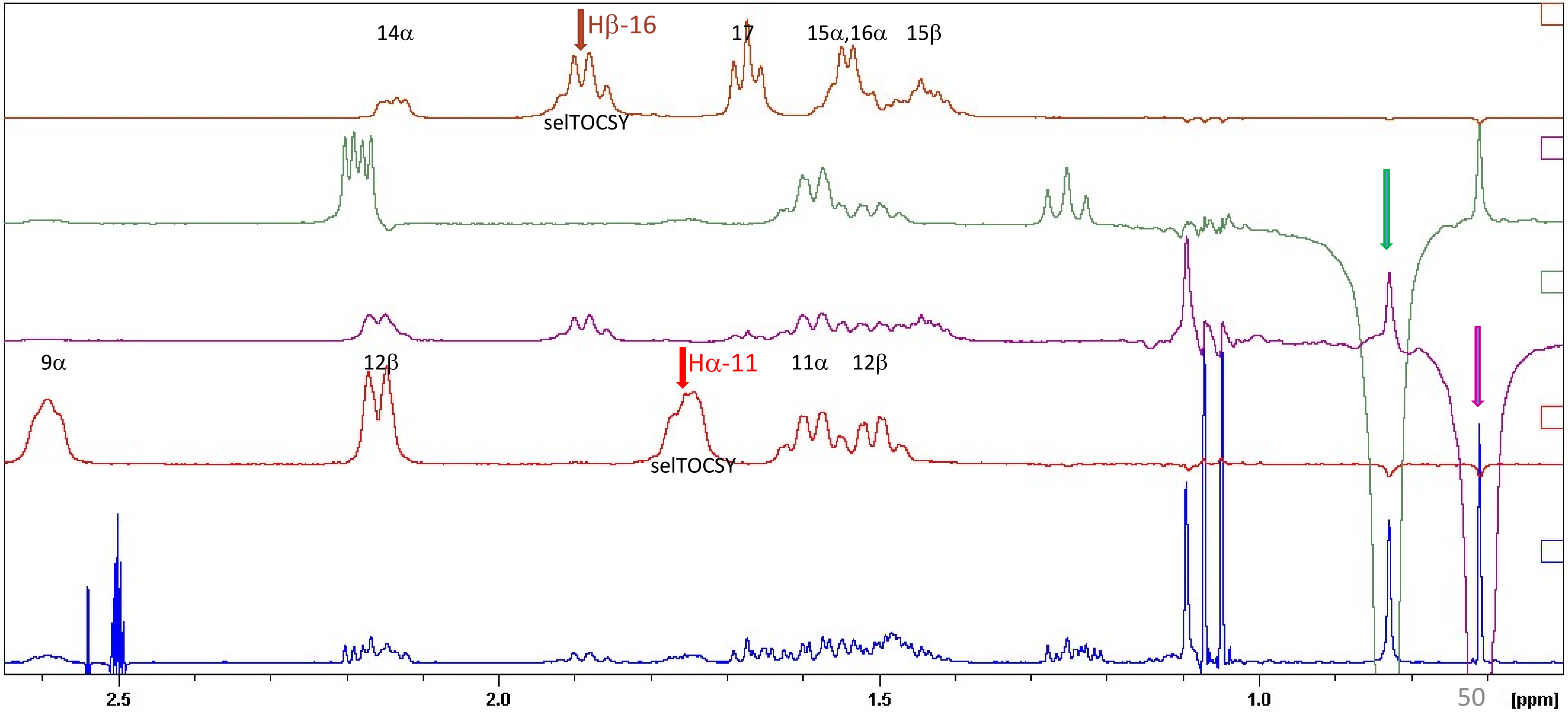

S50 Compound **8**

DEPTQ 125 MHz

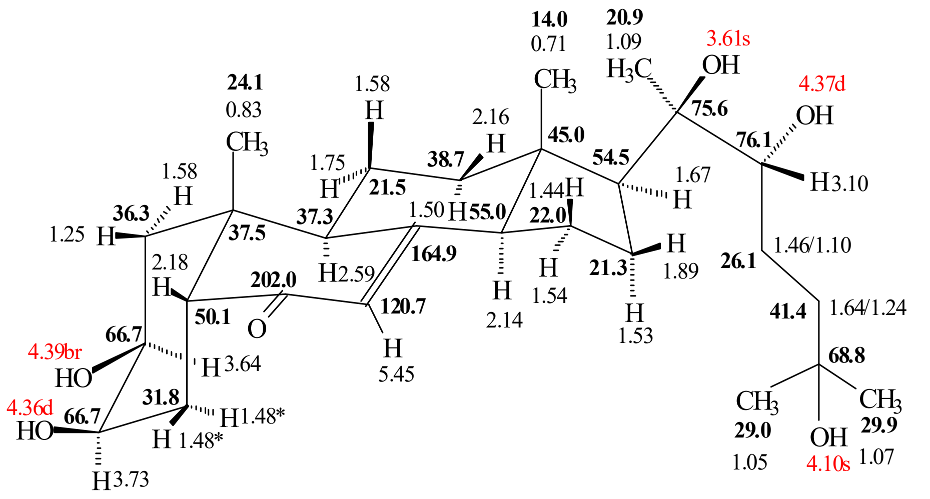

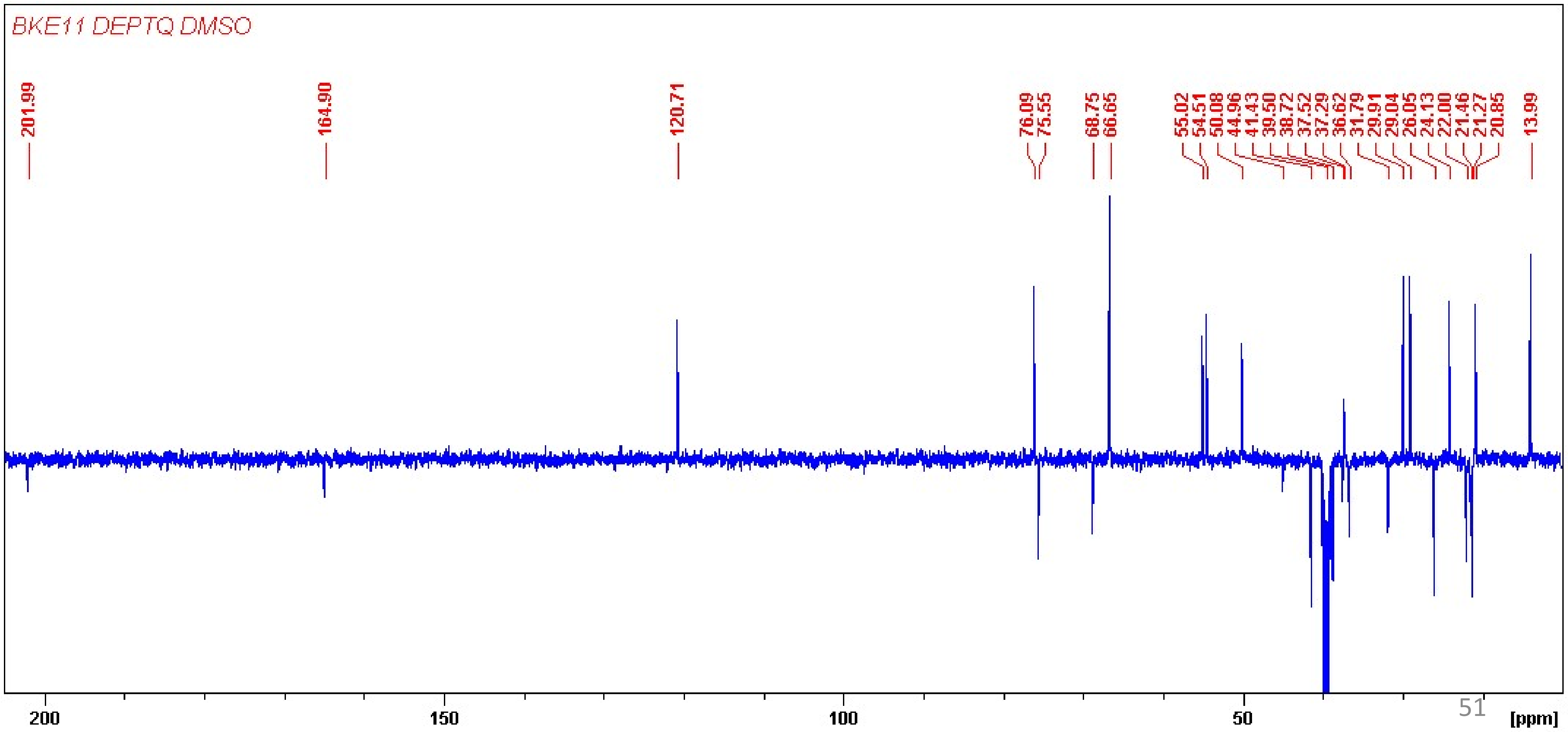

S51 Compound **8**

HSQC

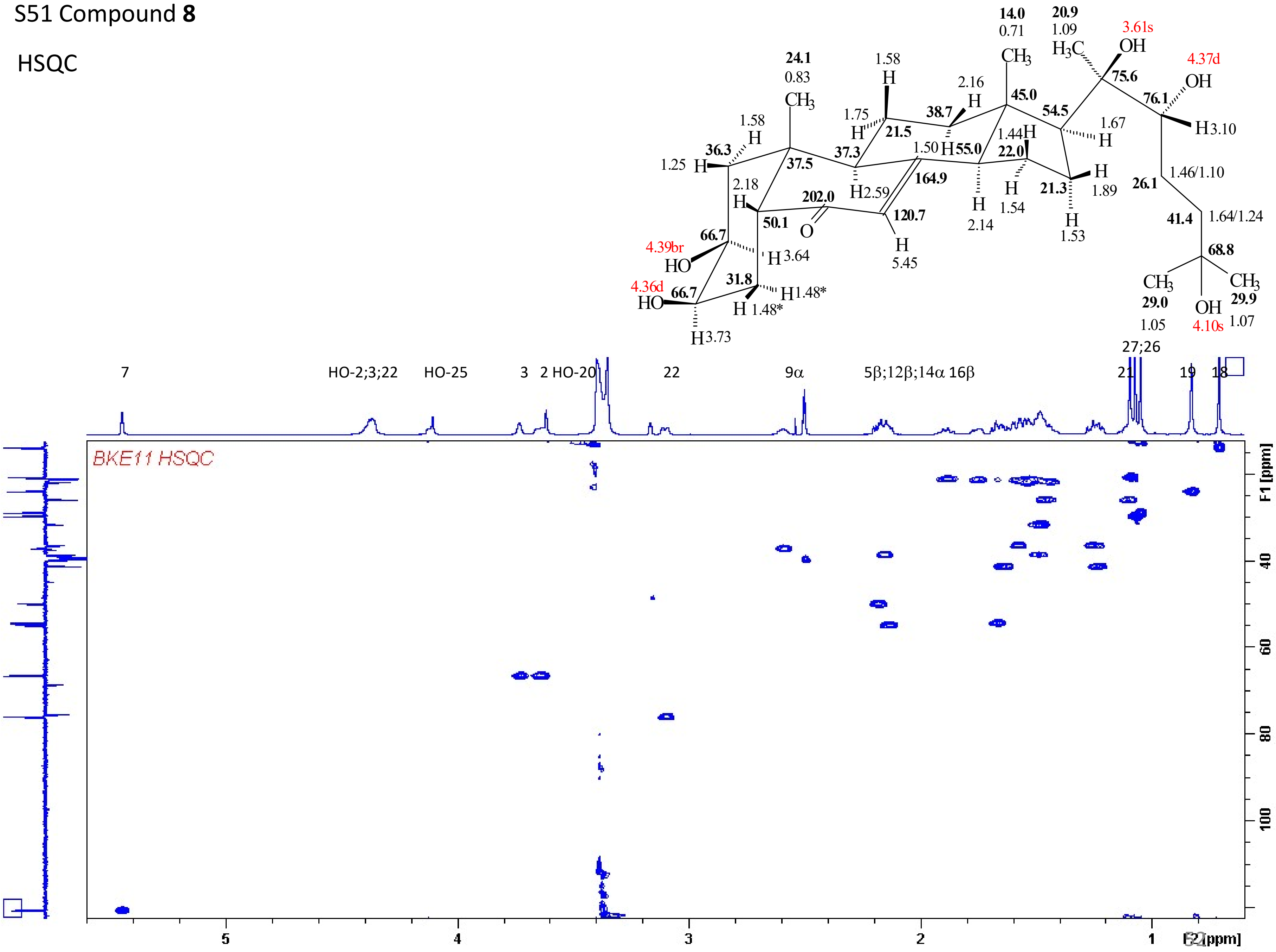

S52 Compound **8**

edHSQC + selHSQC

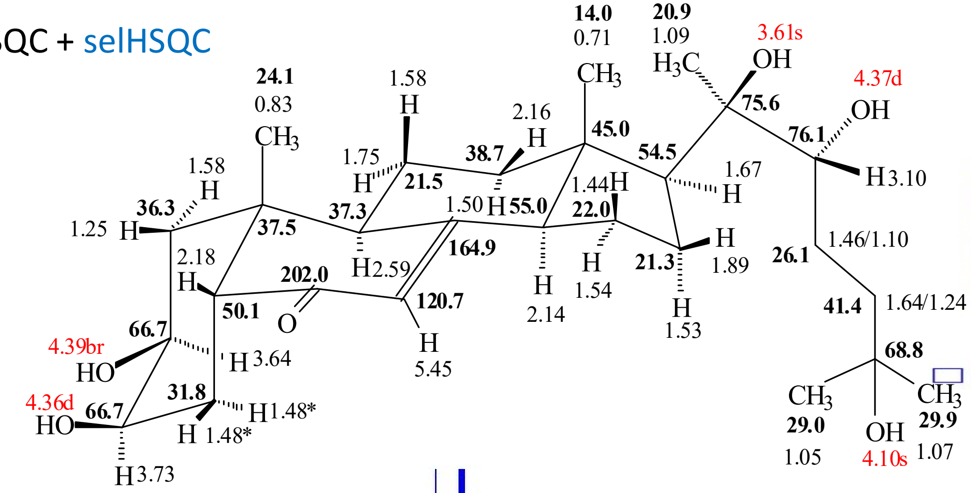

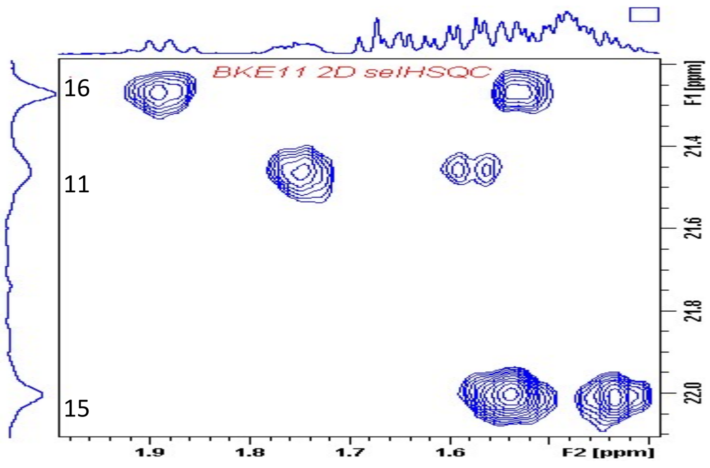

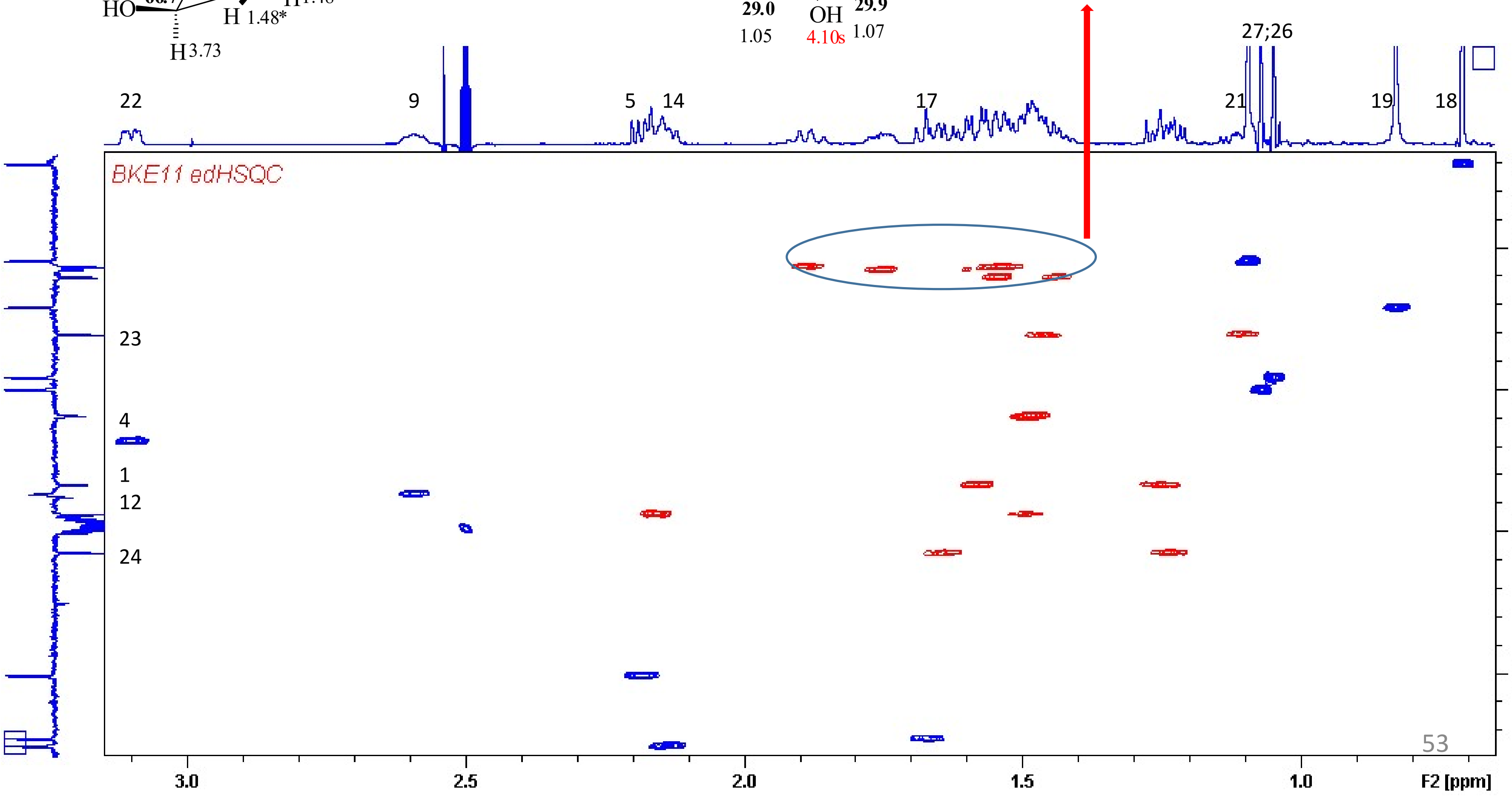

S53 Compound **8**

HMBC

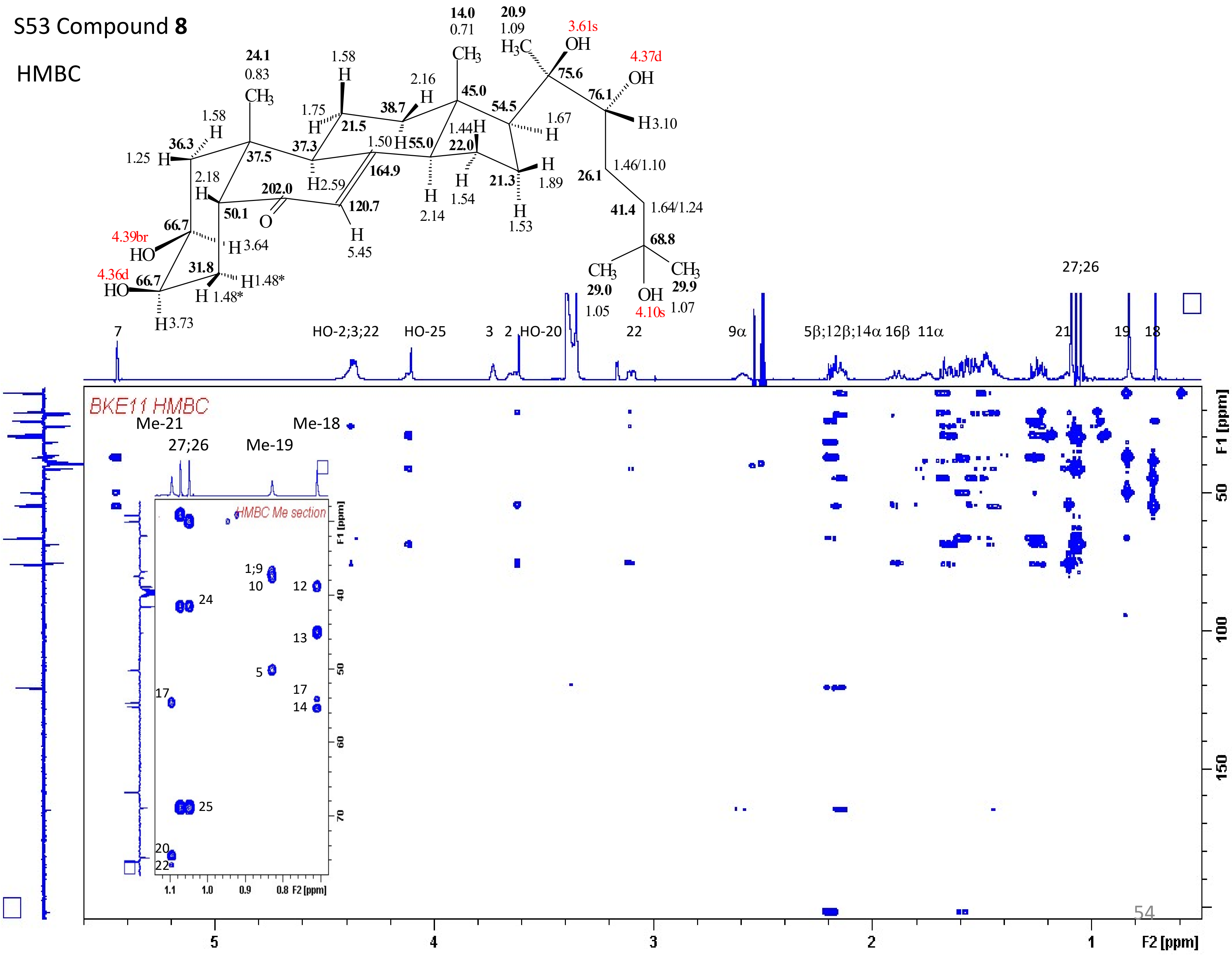

S54 Compound **9**

^{1}H 500 MHz

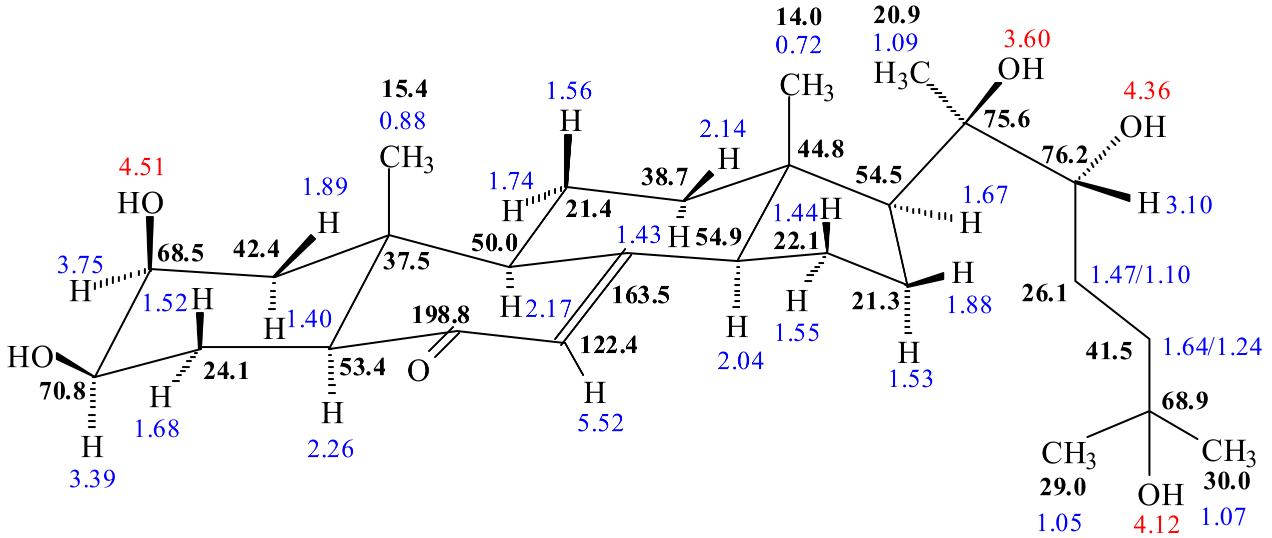

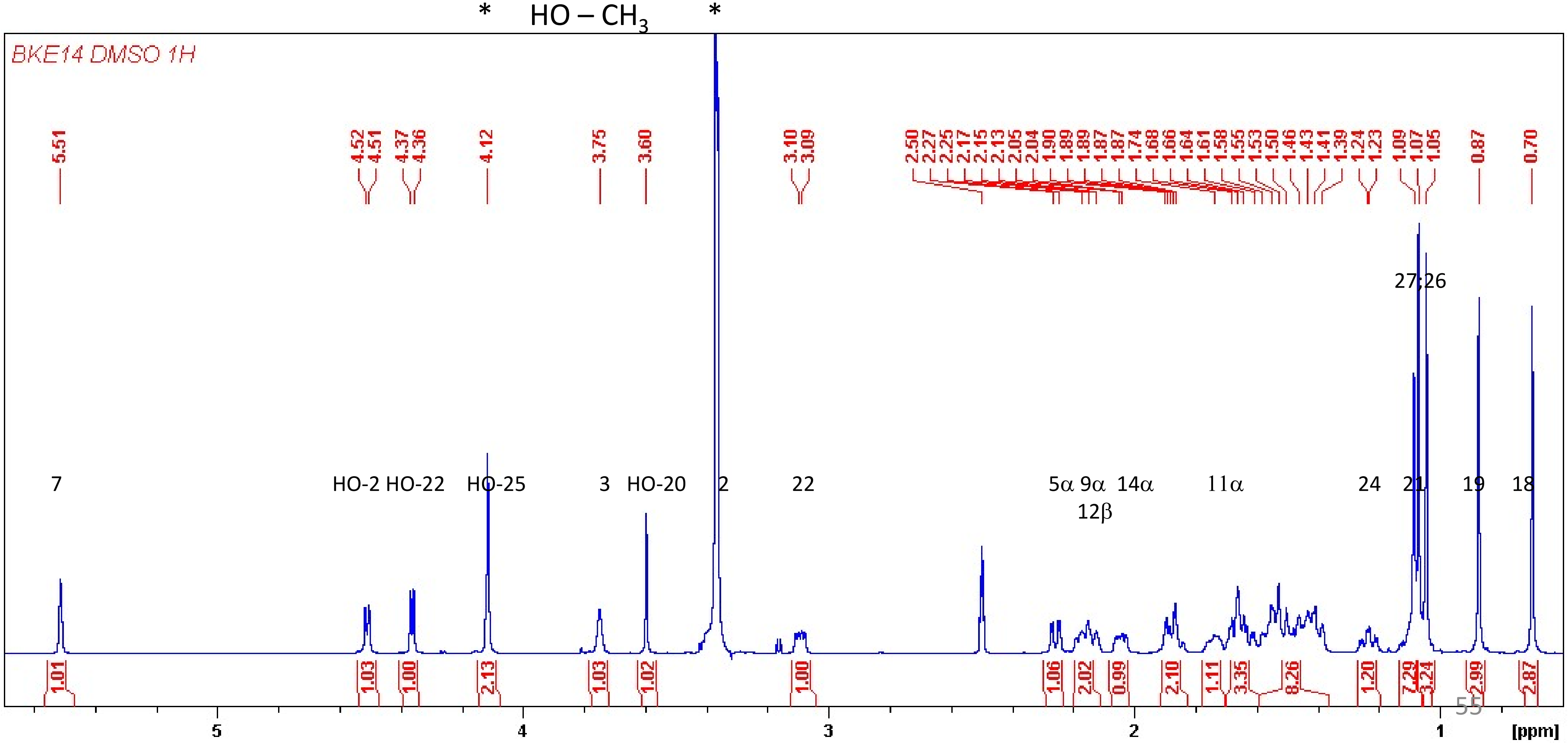

S55 Compound **9**

DEPTQ 125 MHz

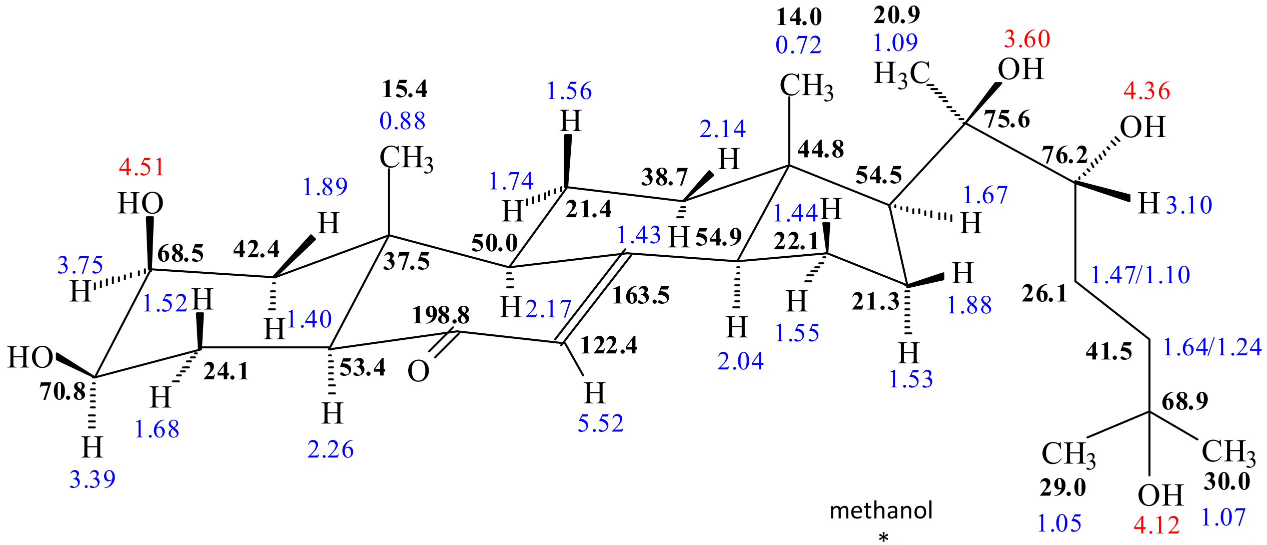

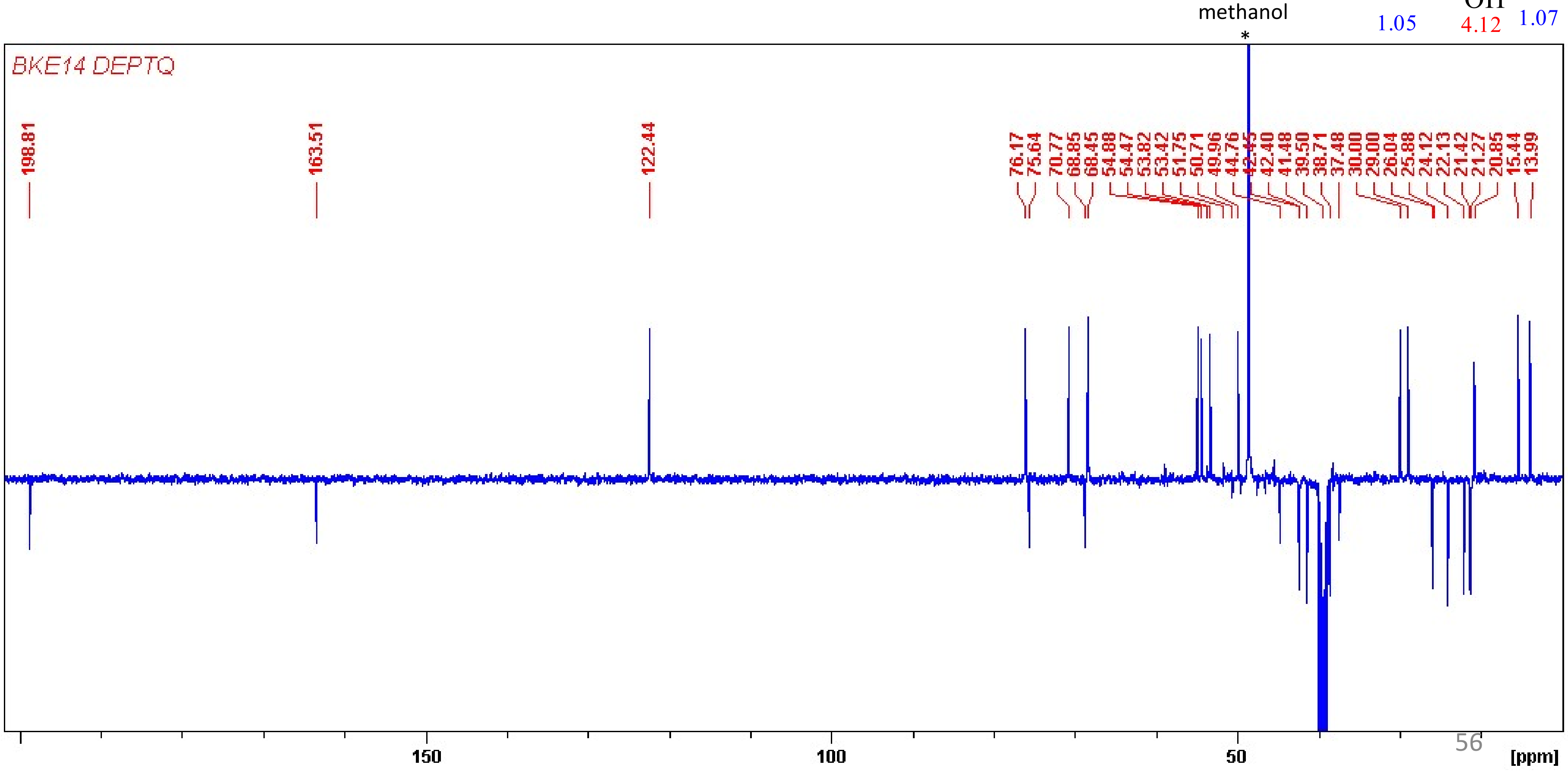

S56 Compound **9**

HSQC

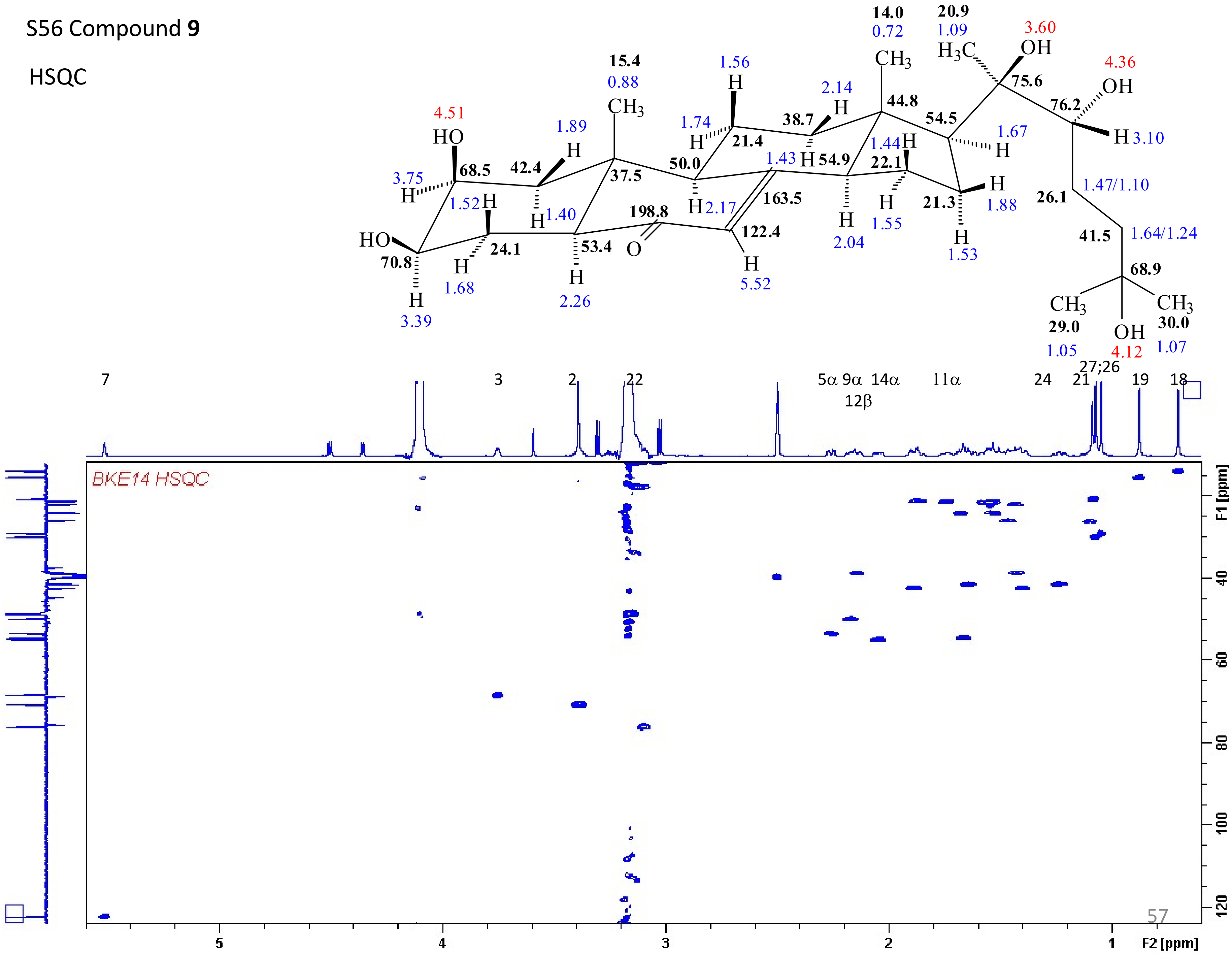

S57 Compound **9**

edHSQC

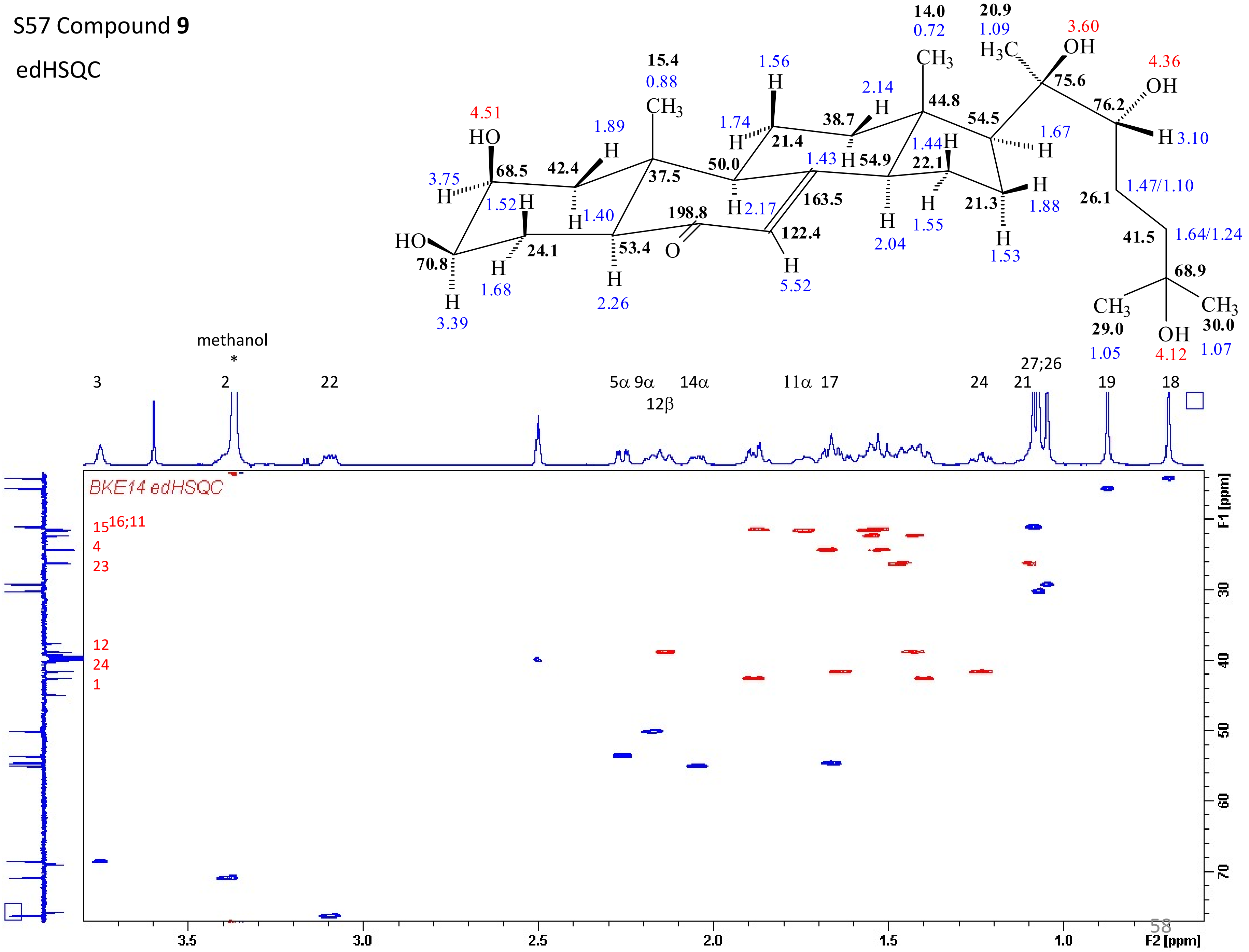

S58 Compound **9**

Band-selective HSQC + inserted selTOCSY on Hα-14 with 10ms and 40ms mixing time

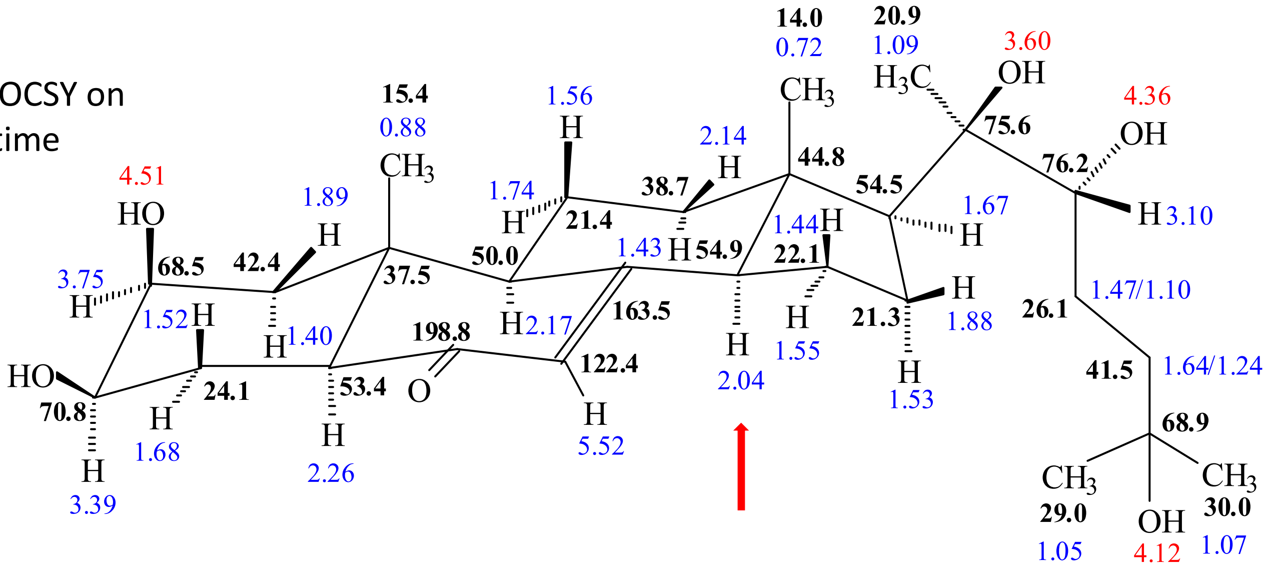

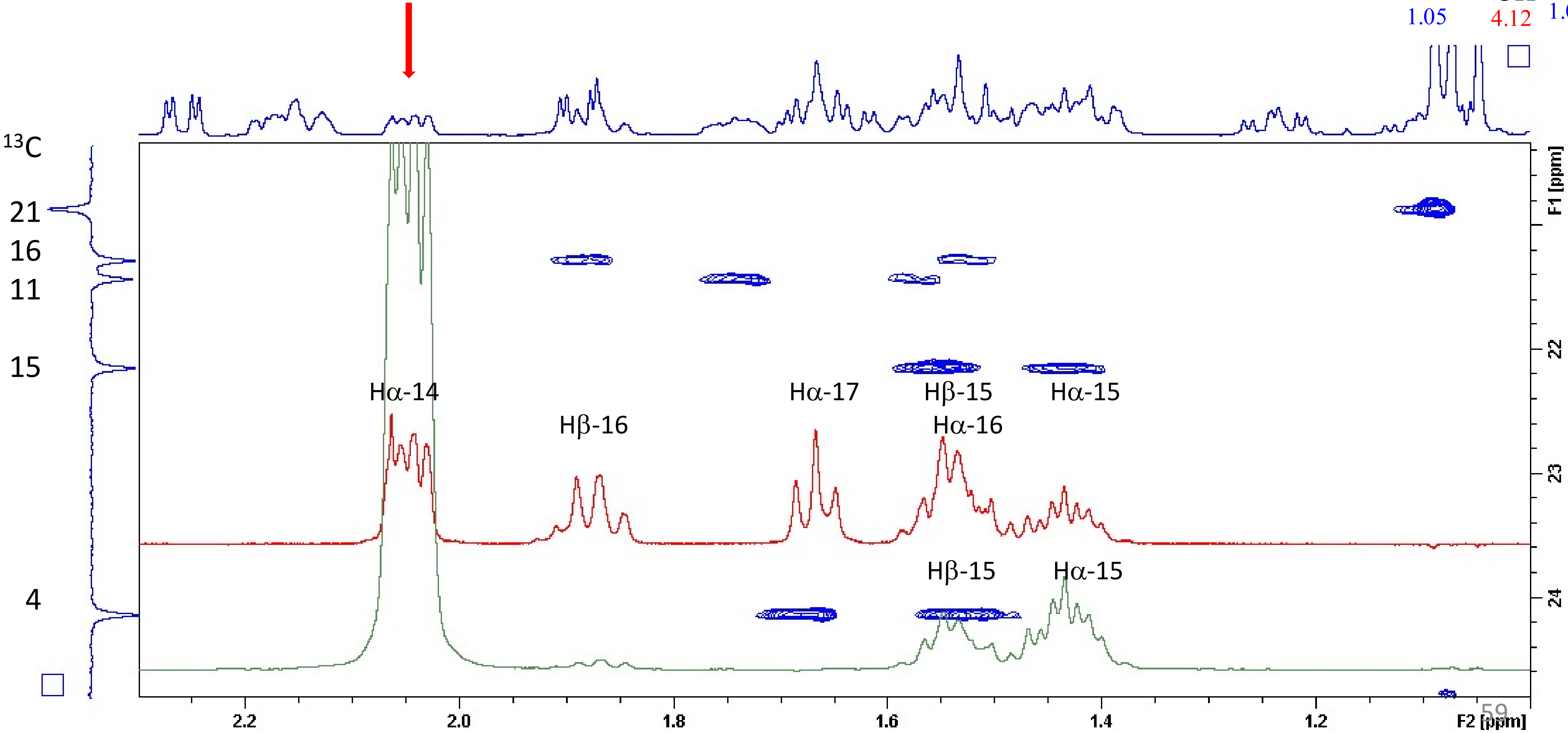

S59 Compound **9**

selROE on CH_3-18, CH_3-19 and Hα-14
mixing time = 300 ms

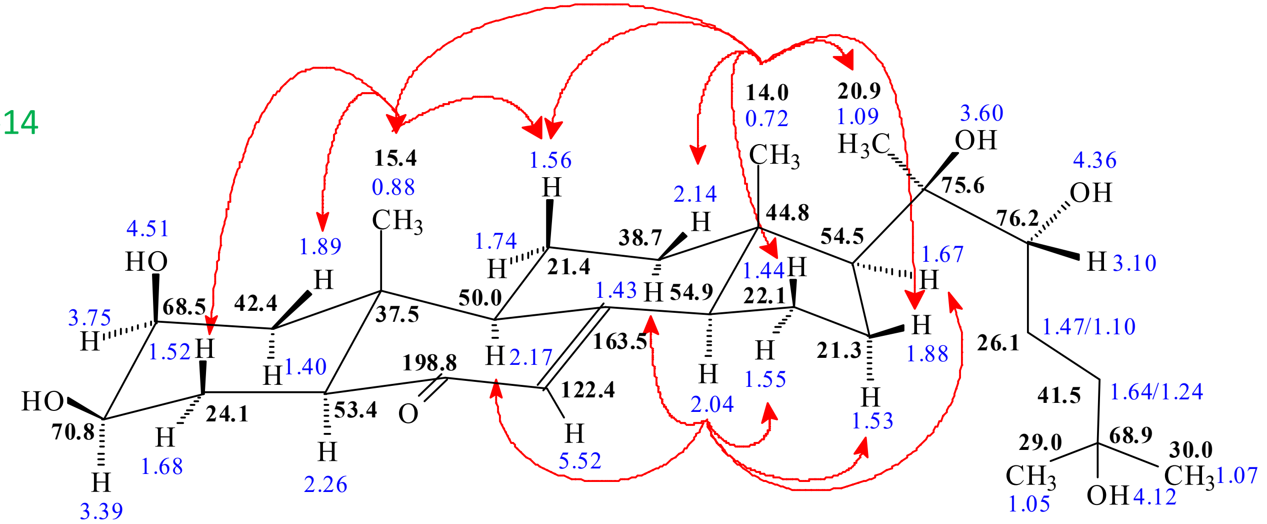

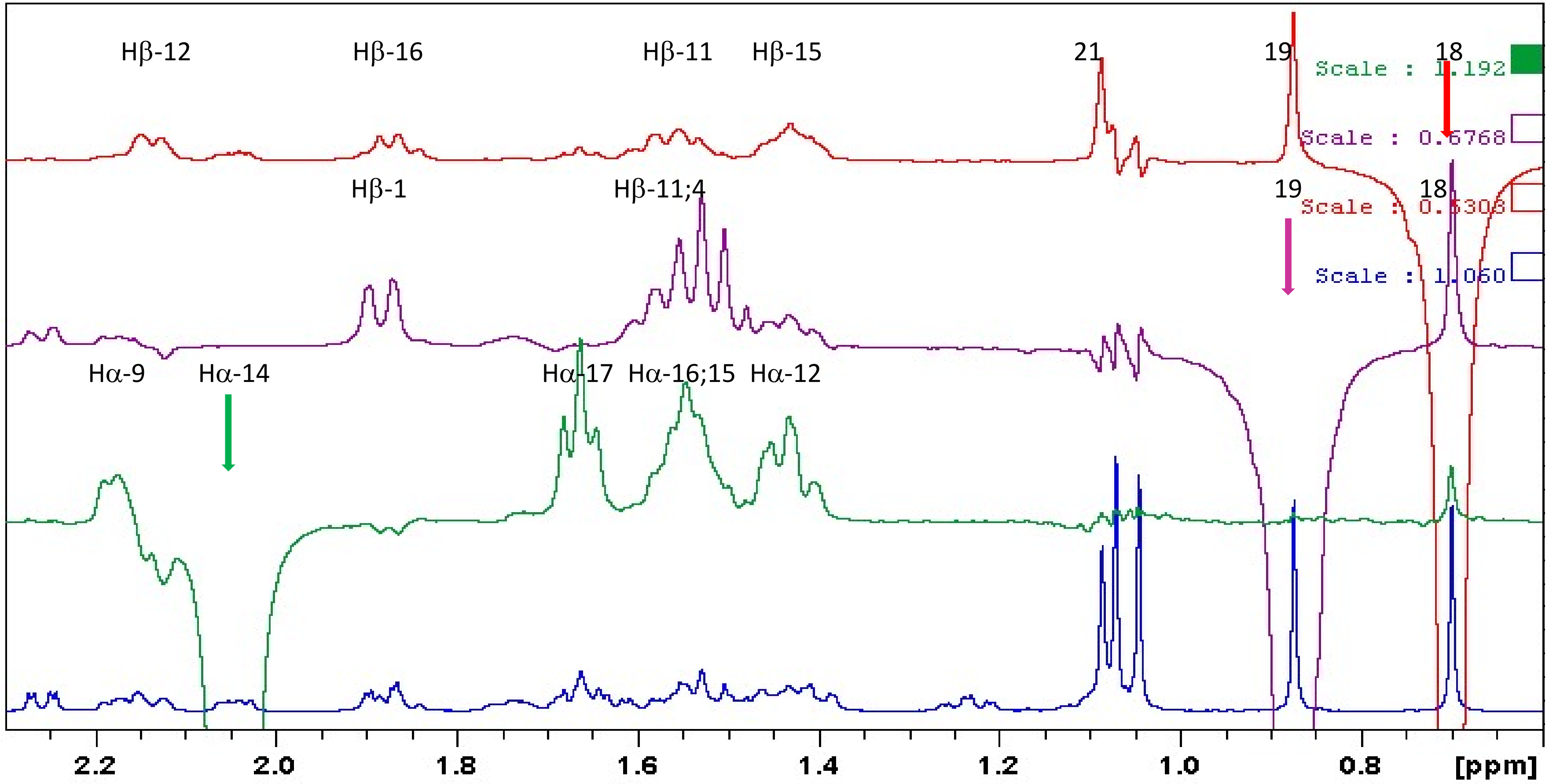

S60 Compound **9**

edHSQC + inserted selROE and selTOCSY on Hα-14

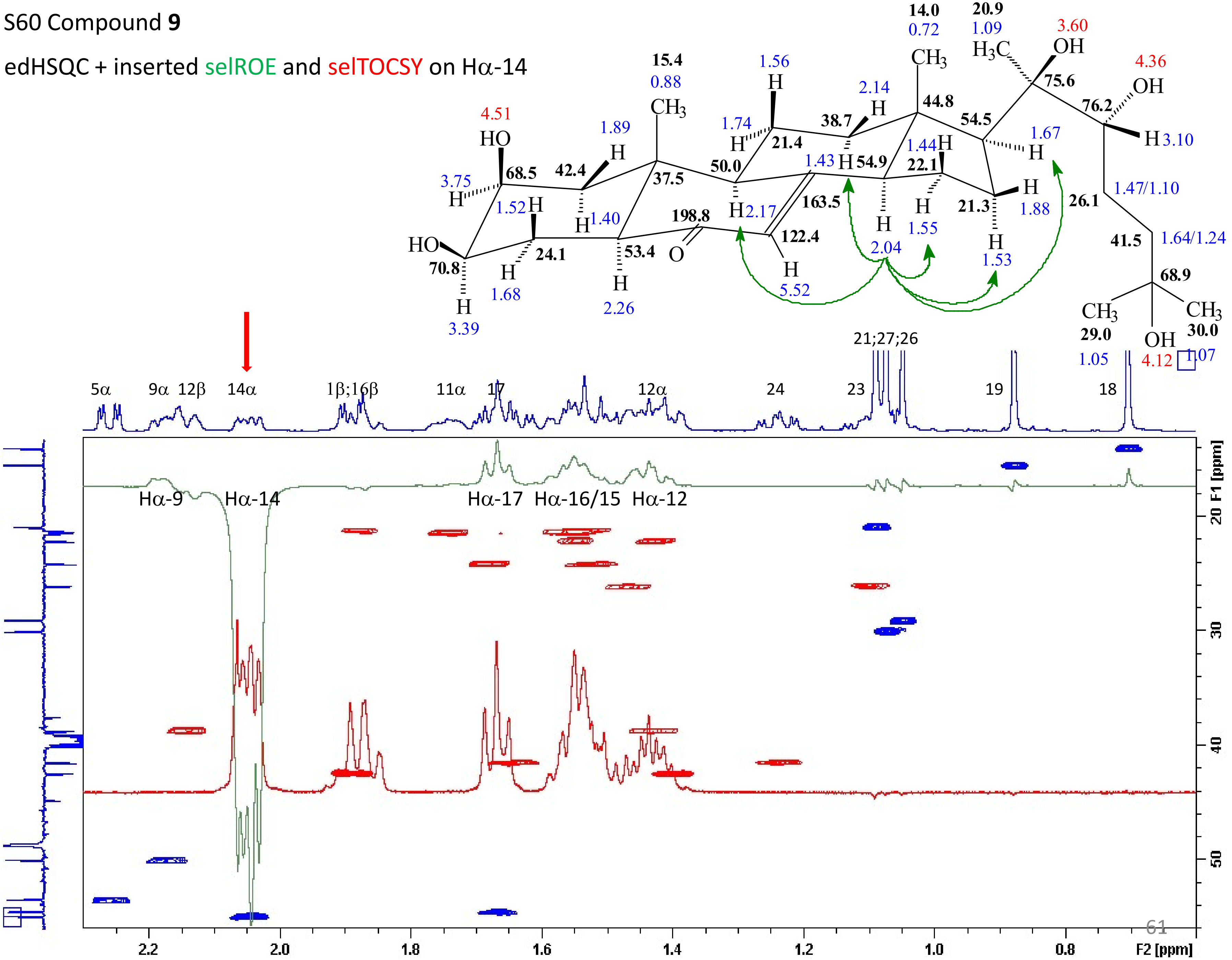

S61 Compound **9**

edHSQC + inserted selROE on Hα-14 and CH_3-18, mixing time = 300 ms

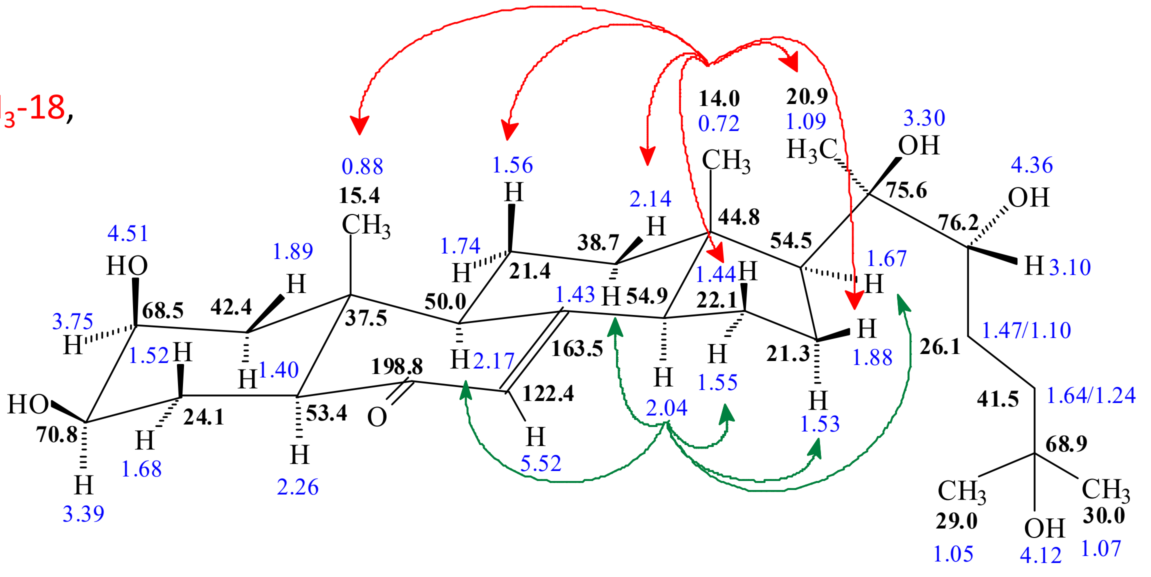

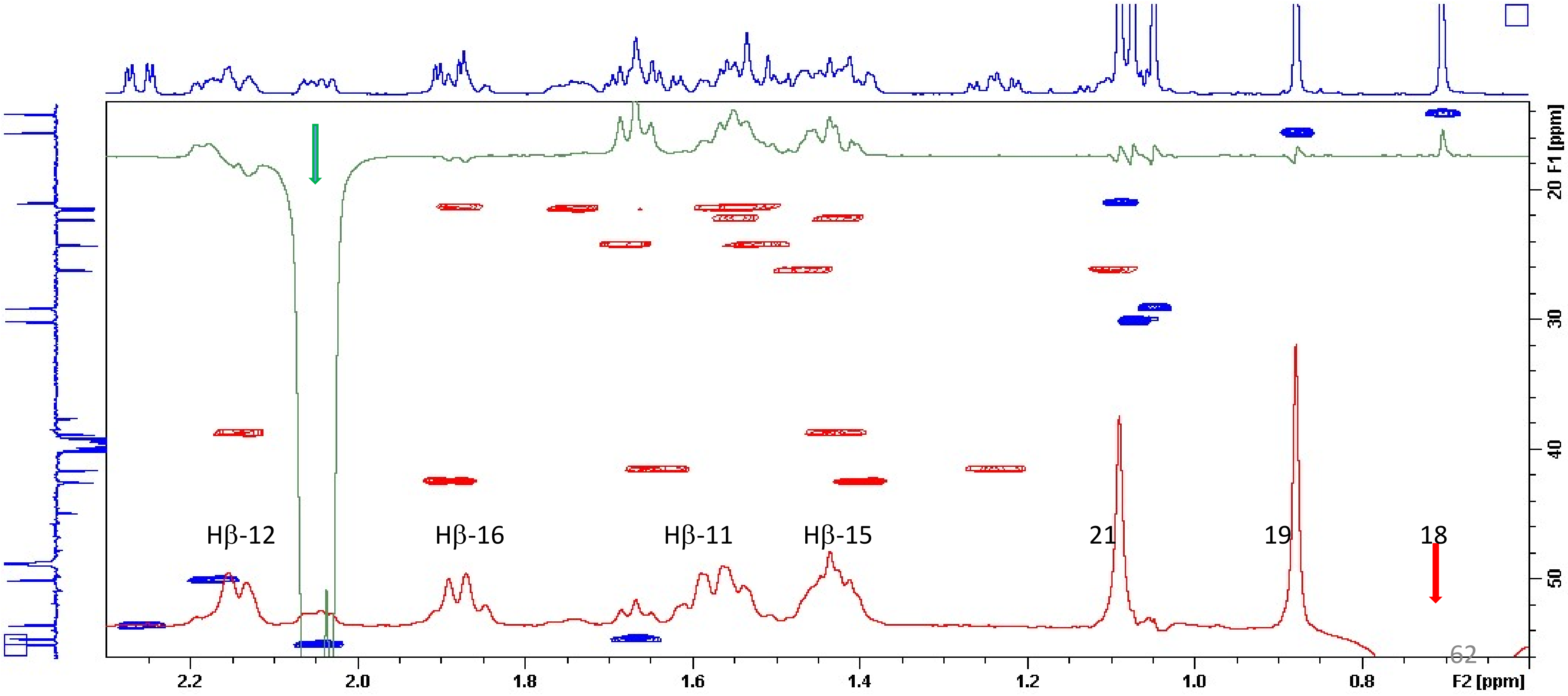

S62 Compound **9**

HMBC

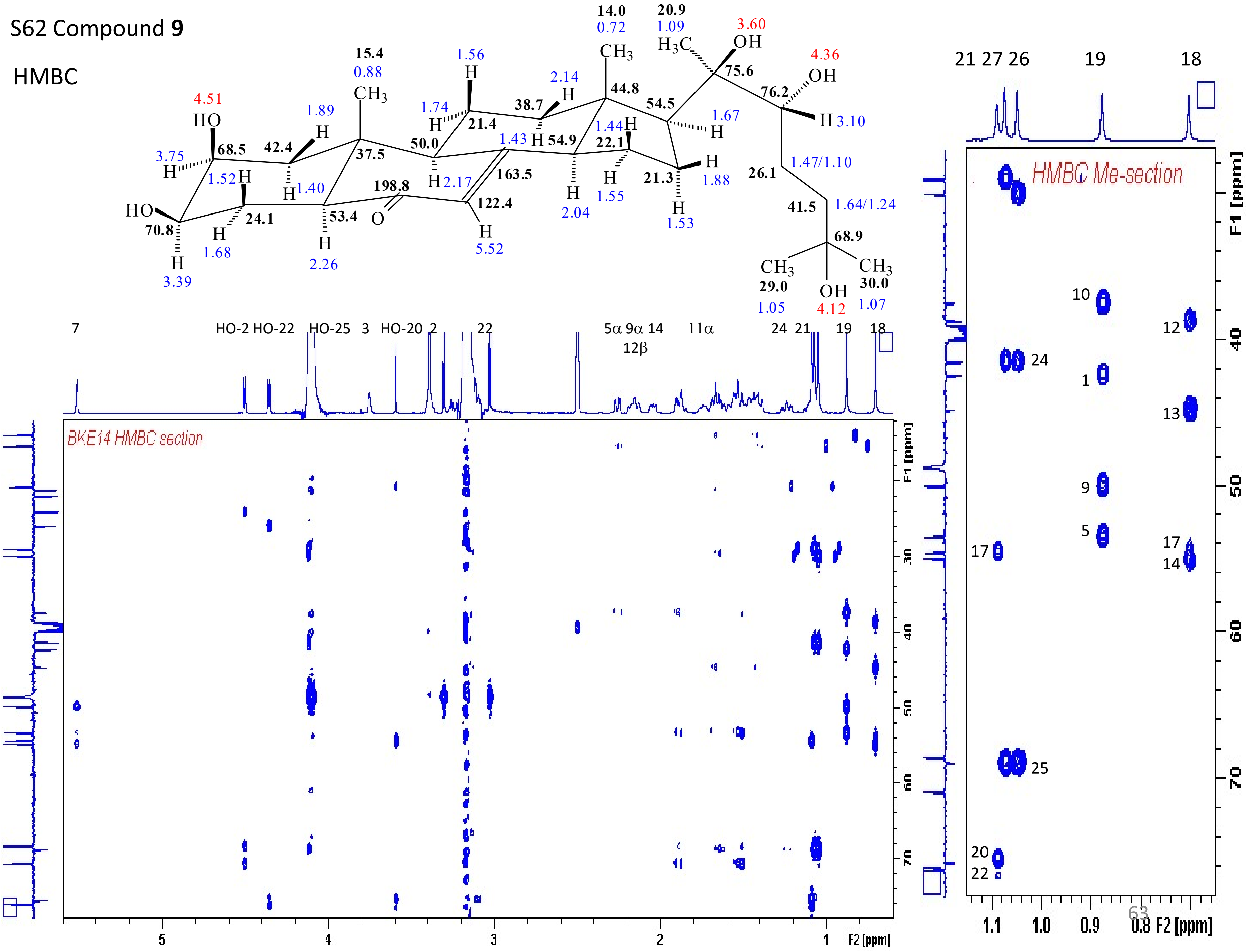

S63 Compound **10**

^{1}H 500 MHz

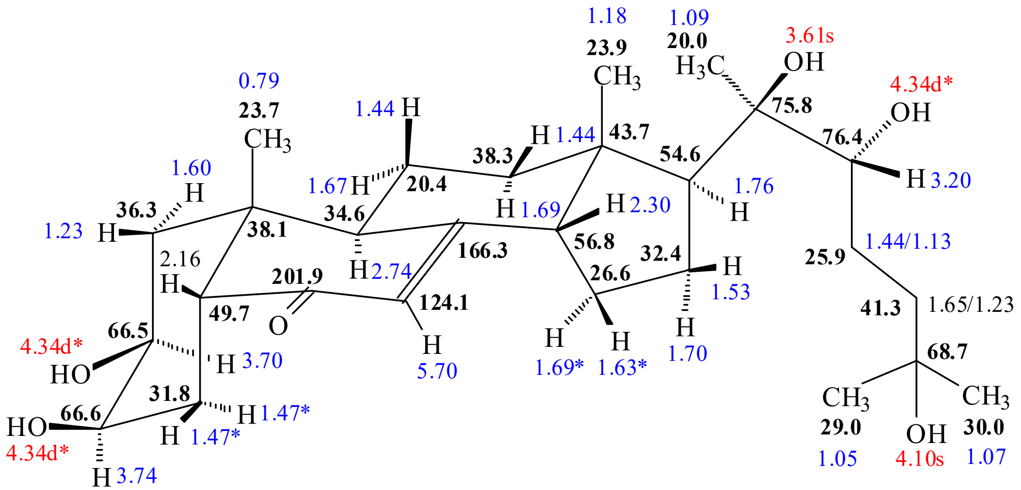

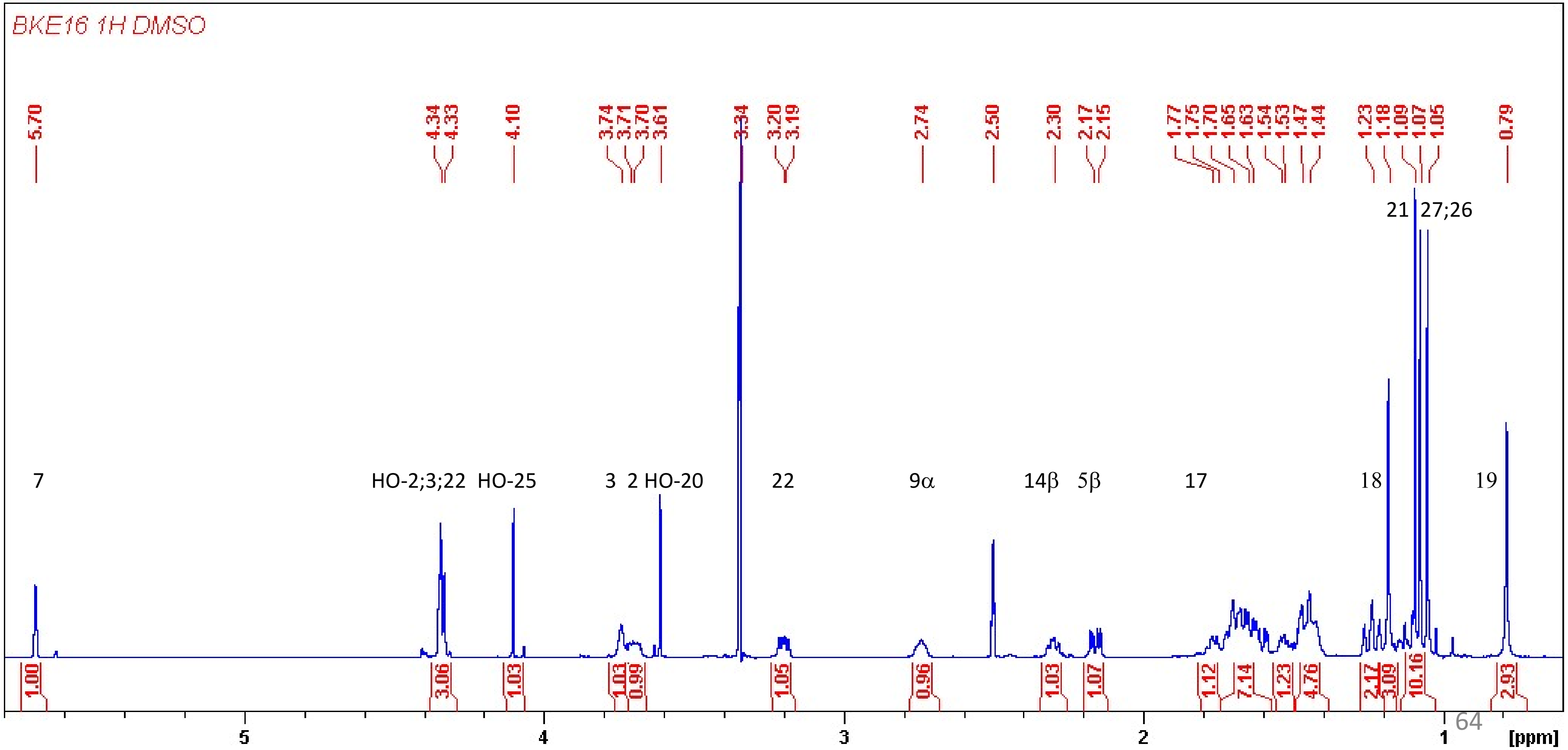

S64 Compound **10**

Supporting of Hβ-14 configuration by selROE

Identification of spin-systems by selTOCSY on Hβ-14 and Hα-9

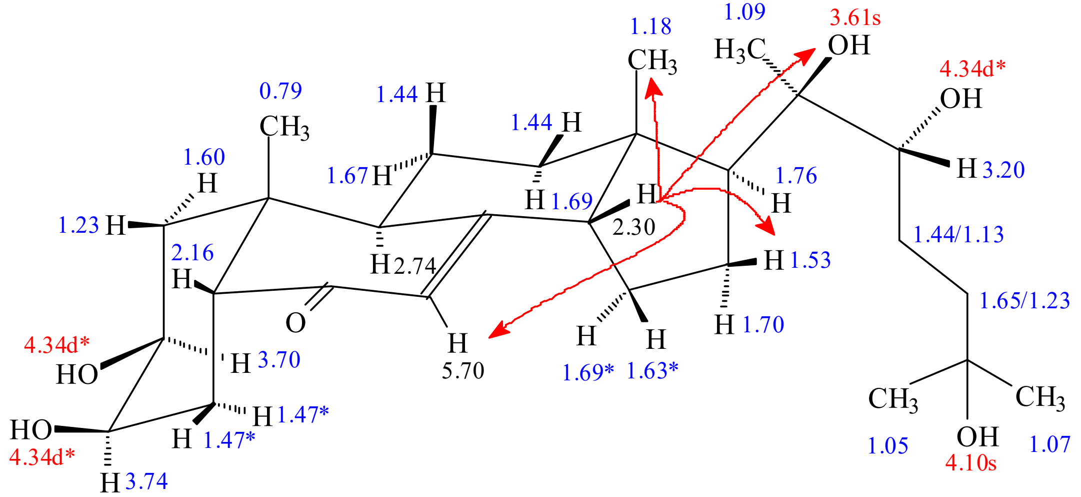

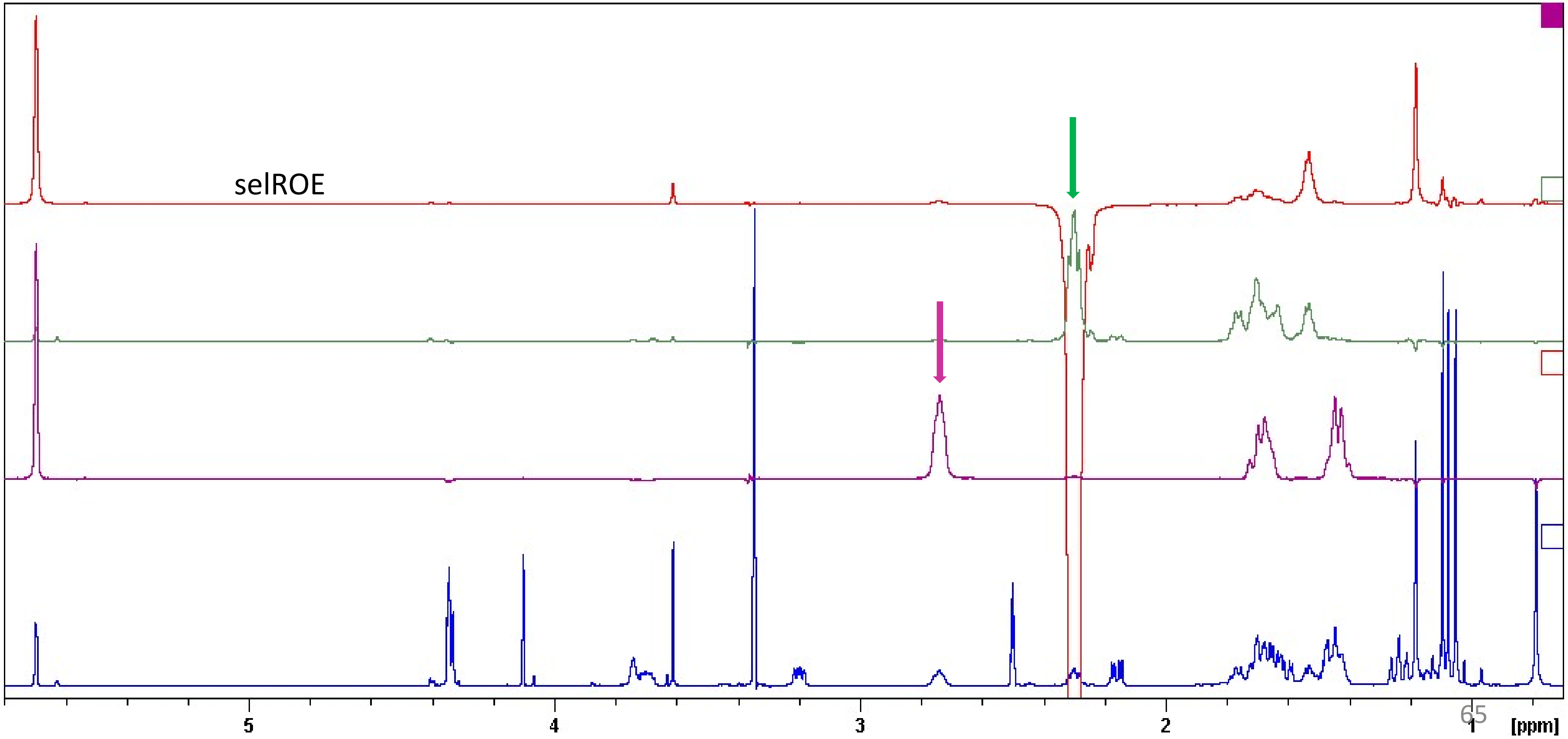

S65 Compound **10**

Steric proximities detected by selROE on Me-18 and Me-19

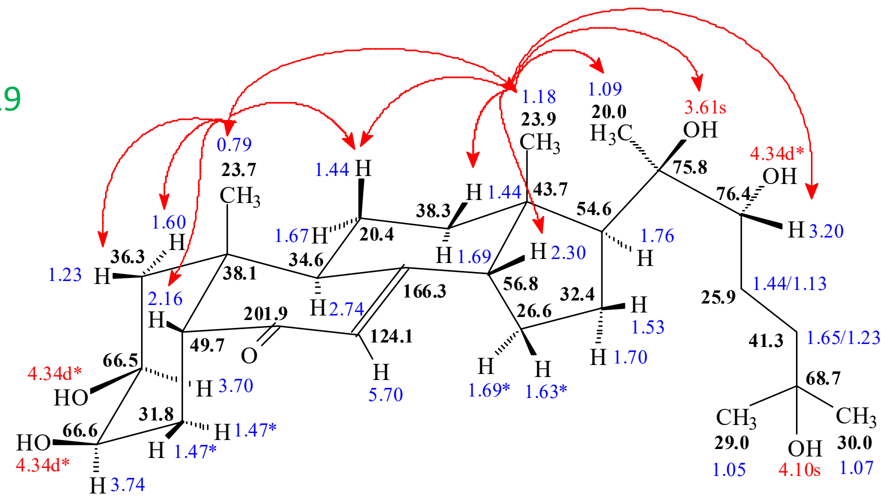

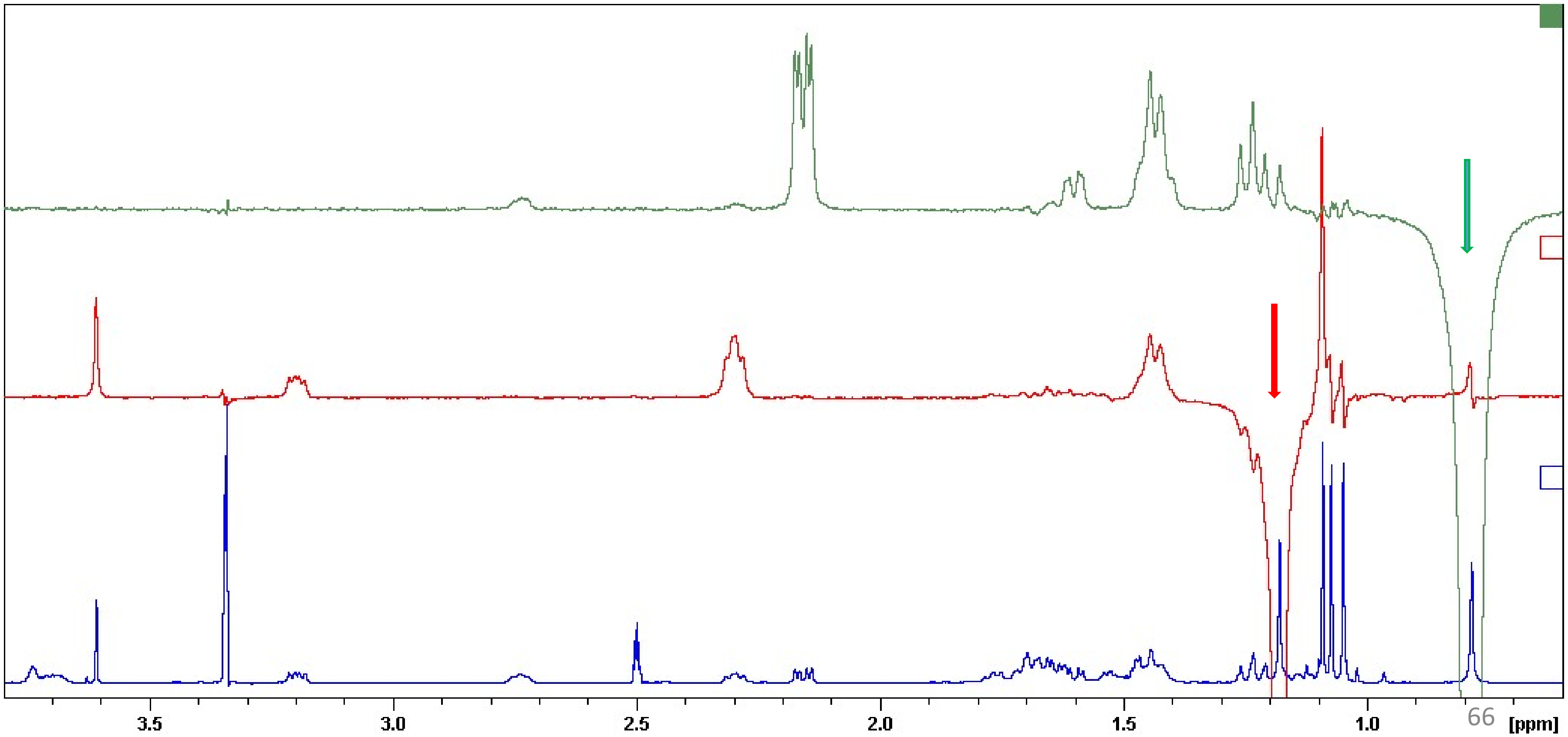

S66 Compound **10**

DEPTQ 125 MHz

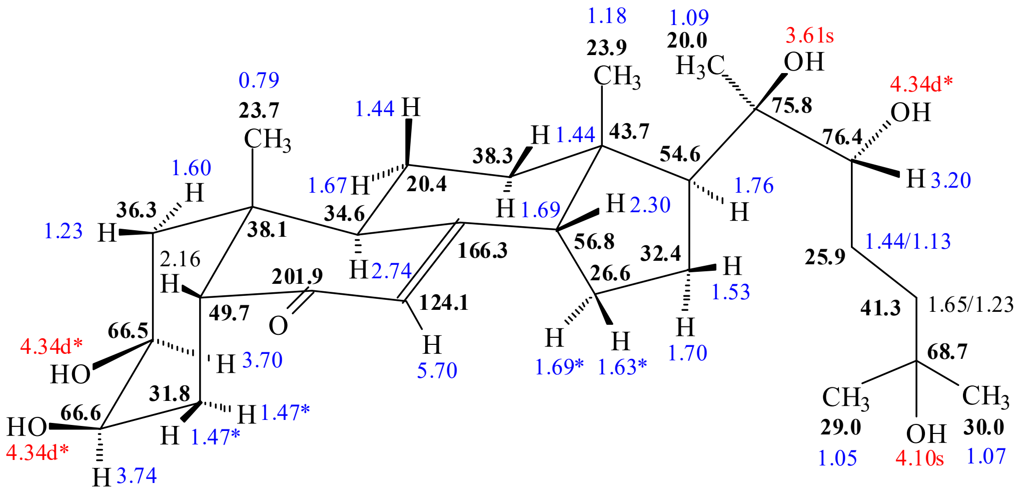

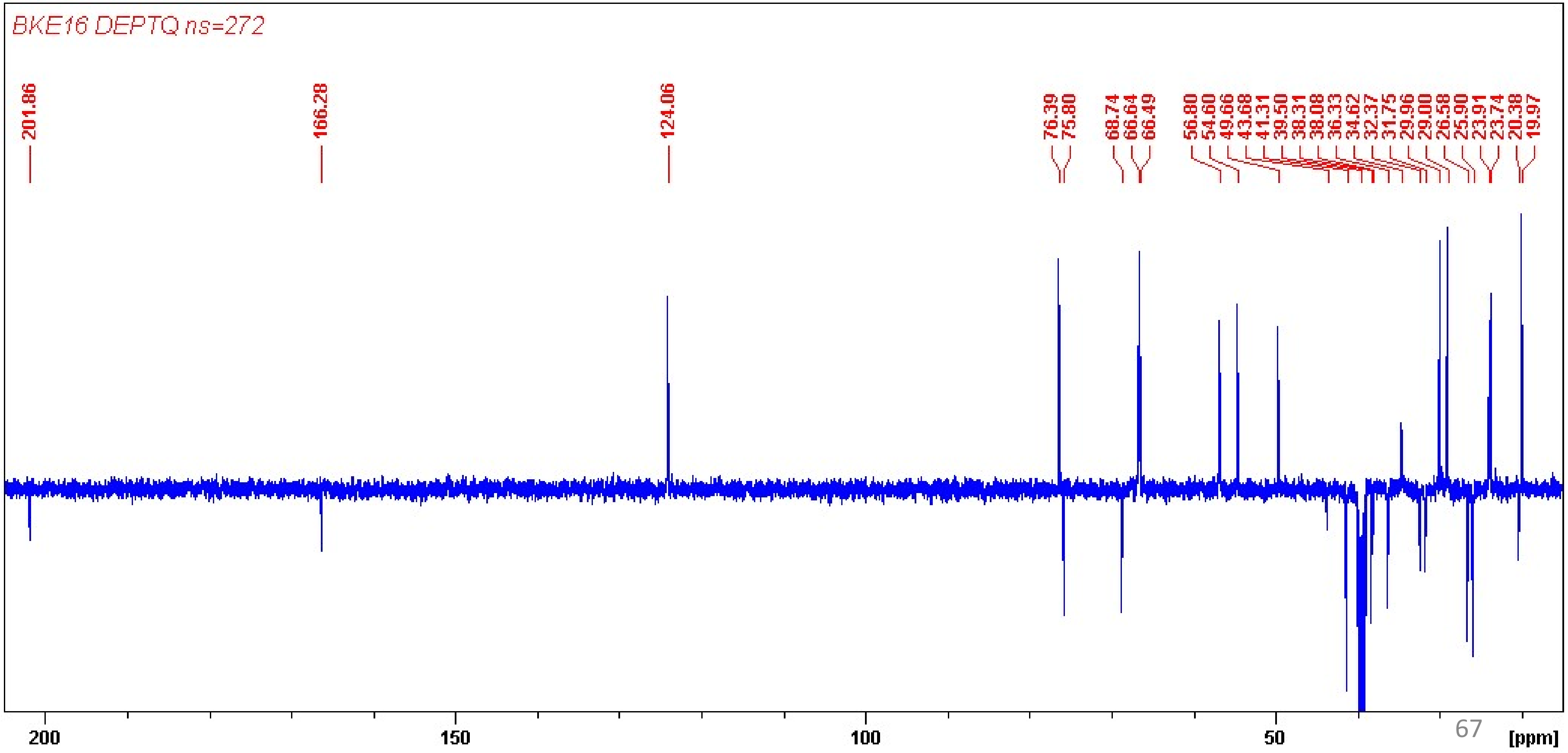

S67 Compound **10**

HSQC

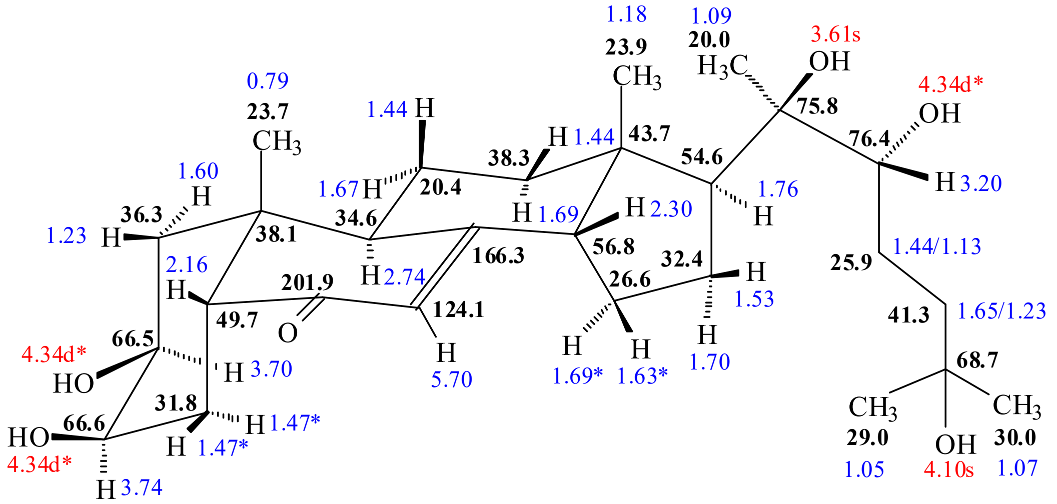

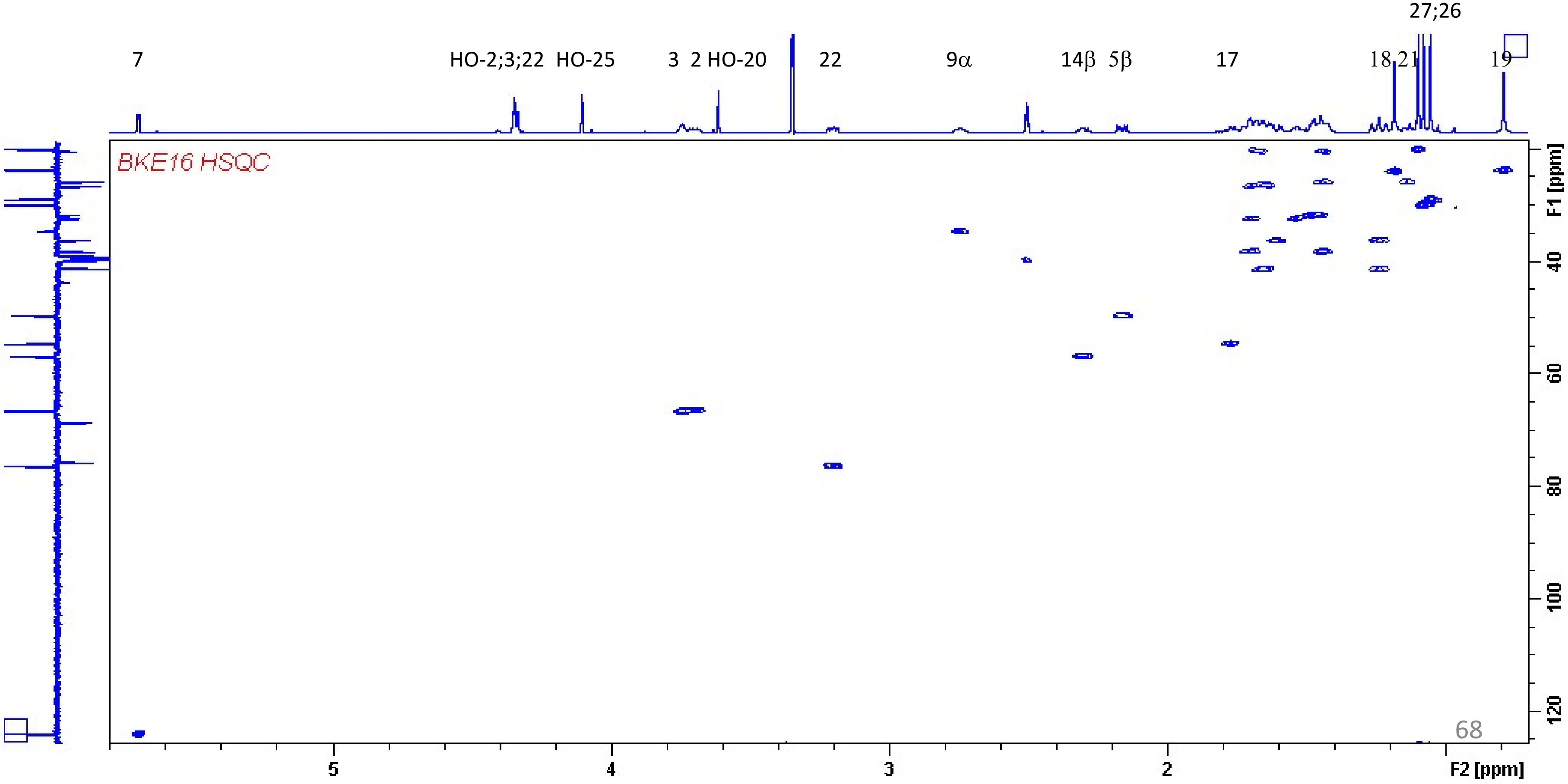

S68 Compound **10**

edHSQC CH_2 section

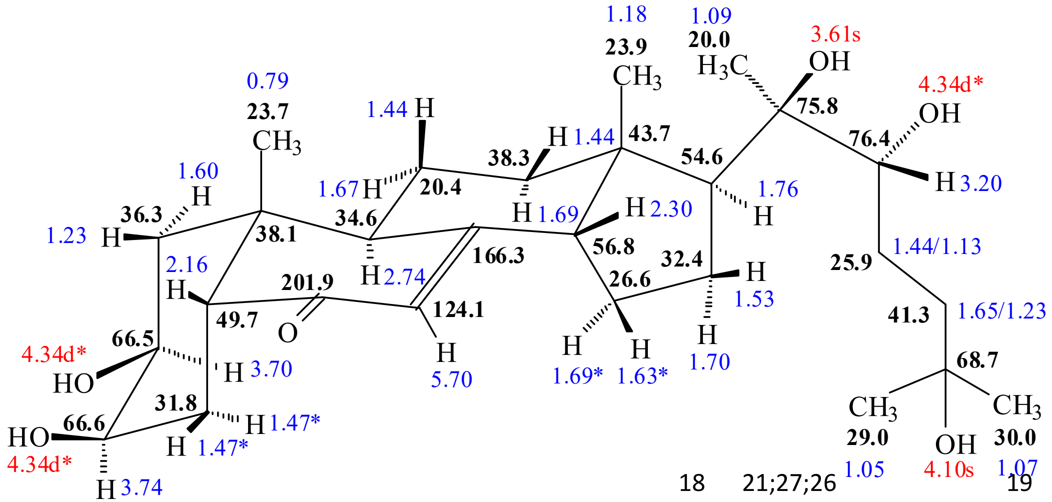

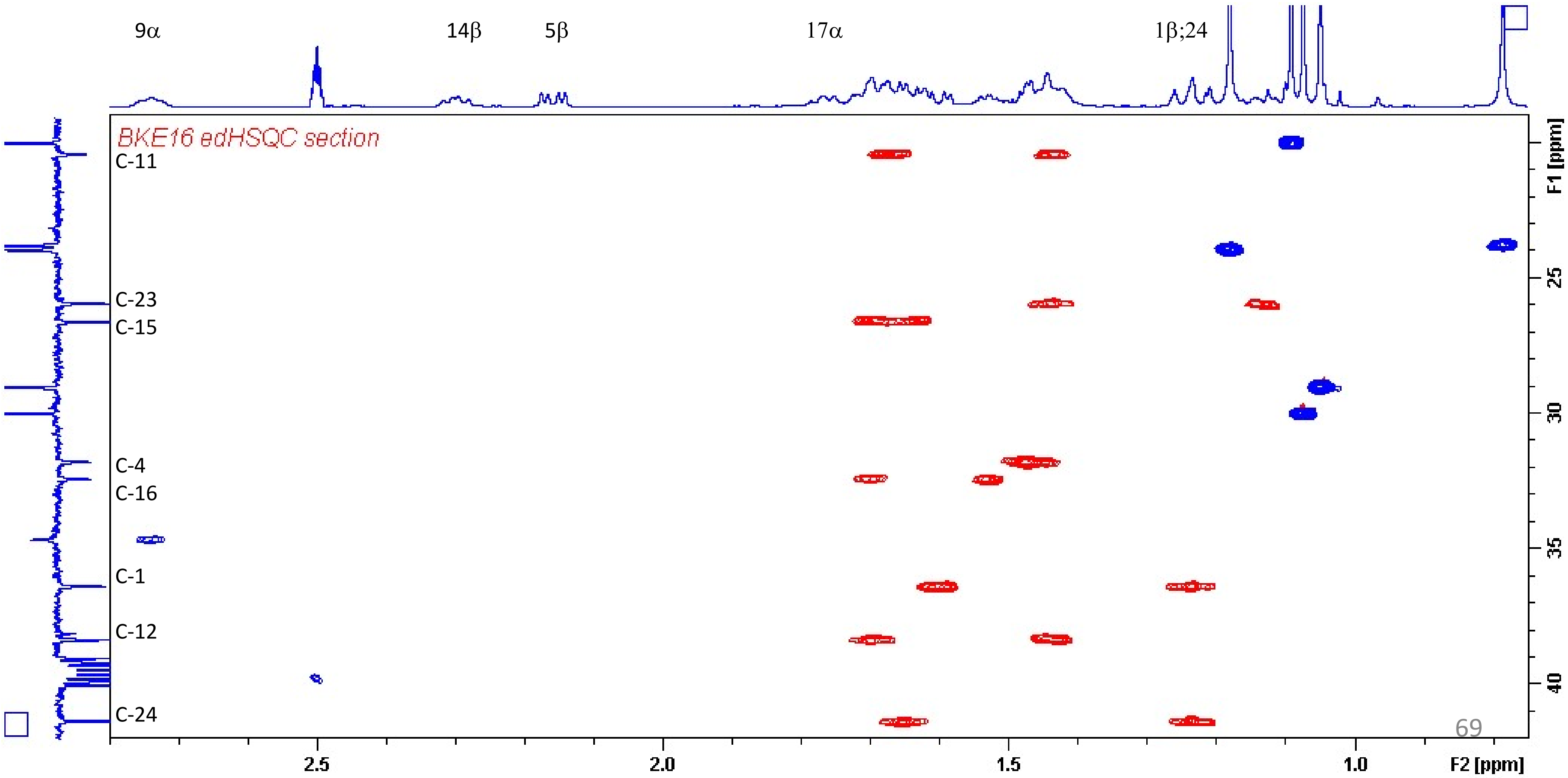

S69 Compound **10**

HMBC

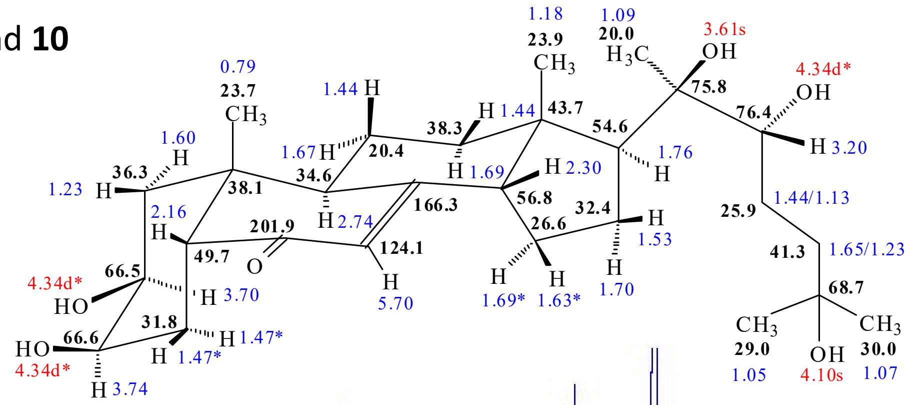

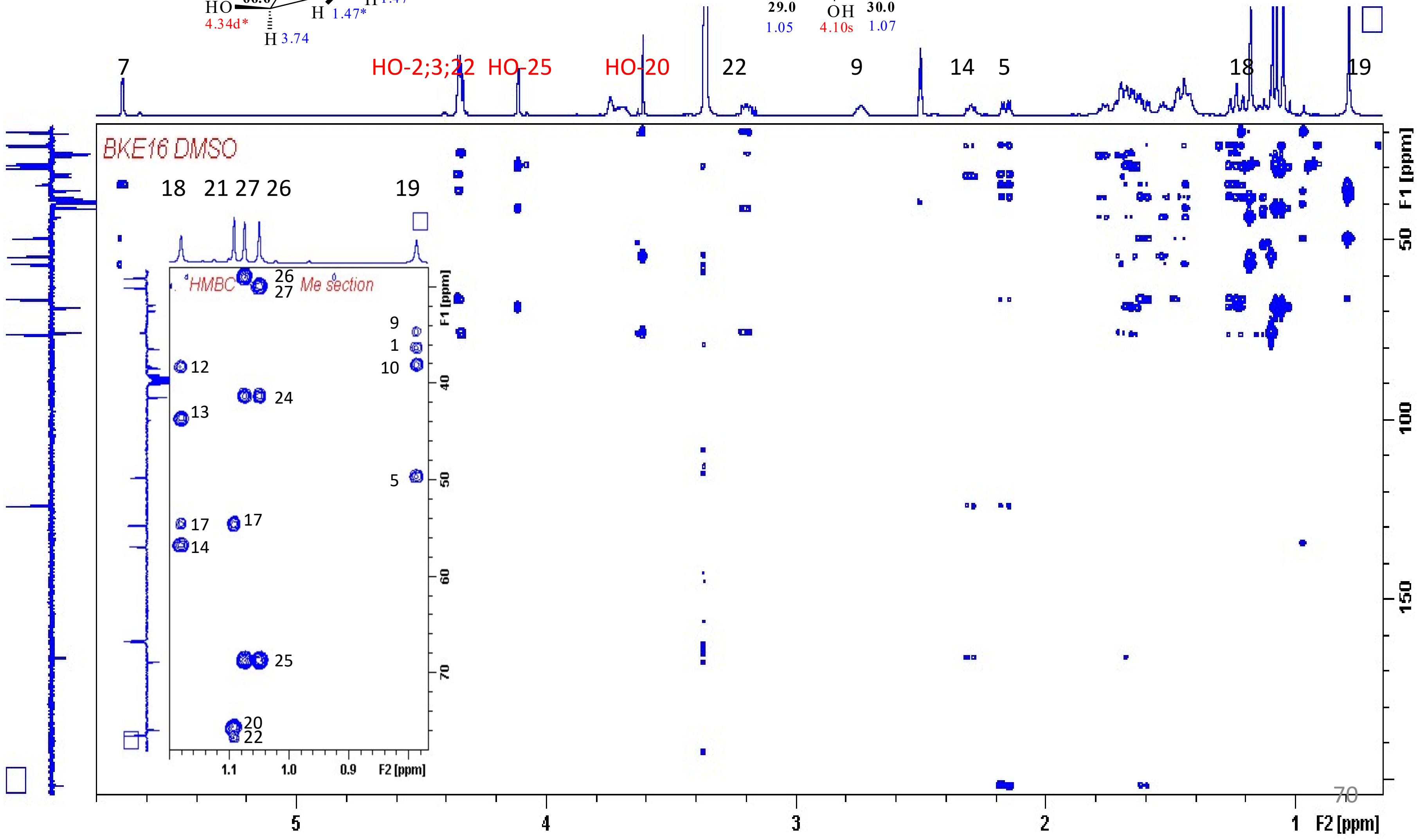

Supplement: Supplementary file 2 — np0c01274_si_002.pdf [file np0c01274_si_002.pdf]
